# Supplementary material for: Transparent artificial intelligence-enabled interpretable and interactive sleep apnea assessment across flexible monitoring scenarios
Source: Nat Commun. 2025 Aug 14;16:7548. doi: 10.1038/s41467-025-62864-x (PMC12354915; doi:10.1038/s41467-025-62864-x)
Supplement: Supplementary file 1 — Supplementary Information [file 41467_2025_62864_MOESM1_ESM.pdf]

SUPPLEMENTARY INFORMATION: TRANSPARENT ARTIFICIAL INTELLIGENCE-ENABLED INTERPRETABLE AND INTERACTIVE SLEEP APNEA ASSESSMENT ACROSS FLEXIBLE MONITORING SCENARIOS

Contents

**Table**	**Table S1**	Statistical analysis of subject characteristics across different SA severity groups
	**Table S2**	Performance of the All-Sub model (trained on *n*=5,255 subjects) in four-level SA analysis
	**Table S3**	Performance of the Non-overlap model (trained on *n*=2,789 subjects) in four-level SA analysis
	**Table S4**	Performance metrics summary for temporal stability analysis across SHHS1 and SHHS2 cohorts
	**Table S5**	Proportional distribution of ethnicities in different cohorts
	**Table S6**	Comparative performance of AIX across ethnicities in different channel configuration scenarios
	**Table S7**	Cross-test cohort performance of AIX in different channel configuration scenarios (AHI cutoff of 5)
	**Table S8**	Cross-test cohort performance of AIX in different channel configuration scenarios (AHI cutoff of 15)
	**Table S9**	Cross-test cohort performance of AIX in different channel configuration scenarios (AHI cutoff of 30)
	**Table S10**	Statistical analysis of AIX interactivity in real-world scenarios
	**Table S11**	Validation of AIX interaction performance in real-world scenarios
	**Table S12**	Parameter description of the fine-grained prediction model TSD-Net
	**Table S13**	Quantitative comparison of interpretability between TSD-Net stages and Grad-CAM (IoU scores)
**Figure**	**Fig. S1**	Data partition of the Non-overlap SHHS model for performance evaluation and temporal stability analysis
	**Fig. S2**	Performance of the Non-overlap SHHS model on AHI regression and SA severity classification
	**Fig. S3**	Comparison of Non-overlap SHHS and All-Sub SHHS models on four-level SA classification performance
	**Fig. S4**	Performance comparison of channel configurations for SA severity classification across test cohorts
	**Fig. S5**	AIX performance in four-level SA classification and AHI regression across test cohorts (Flow)
	**Fig. S6**	AIX performance in four-level SA classification and AHI regression across test cohorts (Chest)
	**Fig. S7**	AIX performance in four-level SA classification and AHI regression across test cohorts (SpO_2)
	**Fig. S8**	Temporal performance comparison of four-level SA analysis between SHHS1 and SHHS2 cohorts
	**Fig. S9**	Performance of Macro F1 scores under different sampling rates and channel configurations
	**Fig. S10**	Statistical significance of Macro F1 scores using two-tailed paired t-tests
	**Fig. S11**	Workflow description of AIX for different channel types (single/multiple)
	**Fig. S12**	Detailed structure description of the fine-grained prediction model TSD-Net
	**Fig. S13**	Detailed structure description of the AHI regression model
	**Fig. S14**	ROC curve evaluation of the granularity prediction model TSD-Net for binary respiratory events
	**Fig. S15**	Comparison of parameters and computational efficiency of different deep learning models
	**Fig. S16**	Comparison of different machine learning models for binary respiratory event classification
	**Fig. S17**	Feature-level SHAP distribution analysis for different channel respiratory signals
	**Fig. S18**	Quantitative SHAP magnitude assessment across respiratory channels
	**Fig. S19**	Examples of overnight AIX assessments using real-world single-channel SpO_2 signals
	**Fig. S20**	Visualization of AIX's transparent scale diffusion mechanism (Flow signal)
	**Fig. S21**	Visualization of AIX's transparent scale diffusion mechanism for (SpO_2 signal)

	**Fig. S22**	Comparison of TSD-Net with Grad-CAM for transparent visualization (Flow signal)
	**Fig. S23**	Comparison of TSD-Net with Grad-CAM for transparent visualization (SpO_2 signal)
	**Fig. S24**	Quantitative evaluation of interpretability alignment between model attention and expert annotations
	**Fig. S25**	Reliability analysis of AIX assessments using single-channel SpO_2 signals
	**Fig. S26**	Correlation analysis between AIX-generated SA risk coefficients and AHI in different test cohorts
	**Fig. S27**	Significance analysis of AIX-generated SA risk coefficients across different test cohorts
	**Fig. S28**	Performance analysis of AIX for AHI prediction in the FDU-HSH retrospective cohort
	**Fig. S29**	Distribution and correlation analysis of SA-related risk features under different gender conditions
	**Fig. S30**	Application process of the AIX system across flexible monitoring settings
	**Fig. S31**	Description of the AIX system interface and functionalities
	**Fig. S32**	Example of an AI sleep report automatically generated by the AIX system
	**Fig. S33**	Visualization and analysis of hyperparameter optimization process
	**Fig. S34**	Cost and operational comparison between Lab-PSG, HSAT and AIX system
	**Fig. S35**	Comprehensive validation results on FDU-HSH prospective cohort
	**Fig. S36**	Architecture overview of demographic-assisted AHI regression framework
	**Fig. S37**	Comparison of AHI regression approaches using Gold channel configuration
	**Fig. S38**	Comparison of AHI regression approaches using SpO_2 channel configuration
	**Fig. S39**	Architecture of CLIP-inspired alignment framework
	**Fig. S40**	Performance evaluation of CLIP-inspired alignment framework

Supplementary Table 1 | Clinical baseline characteristics of subjects across four SA severity levels in seven cohorts. Eight features are documented: gender, age, BMI, systolic blood pressure (SBP), diastolic blood pressure (DBP), sleep duration, hourly arousal index, and smoking status. For continuous variables, mean ± SD are reported. For gender, the proportion of males is provided. For smoking status, the proportions of former smoker, non-smoker, and smoker are presented. *p*-values from Kruskal-Wallis tests assess differences across severity groups. Missing data are indicated by dashes.

Cohort	Subject distribution	Gender	Age	BMI	SBP	DBP	Sleep duration	Arousals	Smoking status
**SHHS1 (*n*=5,255)**	Healthy (*n*=927)	24.49%	58.37±11.23	25.87±4.13	122.86±18.82	72.84±10.28	6.36±0.87	12.29±5.72	36.57%/49.51%/13.92%
	Mild (*n*=2,029)	40.12%	62.20±11.06	27.55±4.73	125.48±18.69	72.95±11.15	6.20±0.89	15.86±6.69	40.76%/48.74%/10.50%
	Moderate (*n*=1,419)	60.54%	64.72±10.68	28.94±4.89	129.09±19.18	73.99±11.79	6.05±0.89	20.34±7.95	48.27%/44.54%/7.19%
	Severe (*n*=880)	69.20%	65.34±10.42	30.71±5.52	130.32±18.90	75.26±12.74	5.95±0.89	30.32±14.39	47.73%/44.89%/7.39%
	p-value	$P=7.67\times10^{-109}$	$P=1.21\times10^{-51}$	$P=1.02\times10^{-107}$	$P=1.04\times10^{-23}$	$P=3.64\times10^{-6}$	$P=1.42\times10^{-24}$	$P=0.00$	$P=0.67$
**SHHS2 (*n*=2,522)**	Healthy (*n*=437)	22.88%	63.75±10.48	26.28±4.20	-	-	6.52±0.93	12.33±6.05	36.61%/53.09%/10.30%
	Mild (*n*=941)	39.53%	66.67±10.12	27.65±4.57	-	-	6.47±0.99	15.46±6.82	41.45%/50.90%/7.65%
	Moderate (*n*=687)	55.31%	68.74±9.92	29.23±5.03	-	-	6.27±0.95	18.32±7.32	43.23%/51.53%/5.24%
	Severe (*n*=457)	66.74%	69.86±9.79	30.56±5.66	-	-	6.16±1.04	28.79±12.78	44.64%/50.77%/4.60%
	p-value	$P=6.66\times10^{-46}$	$P=1.04\times10^{-20}$	$P=1.46\times10^{-43}$	-	-	$P=7.44\times10^{-10}$	$P=3.36\times10^{-151}$	$P=0.92$
**MESA (*n*=1,846)**	Healthy (*n*=187)	26.20%	66.55±9.03	-	-	-	6.55±1.14	12.80±5.84	-
	Mild (*n*=571)	33.27%	68.46±8.94	-	-	-	6.43±1.04	16.45±6.59	-
	Moderate (*n*=559)	45.97%	70.29±8.87	-	-	-	6.26±1.02	21.24±8.28	-
	Severe (*n*=529)	62.57%	69.69±8.93	-	-	-	6.09±1.12	31.84±13.39	-
	p-value	$P=7.80\times10^{-27}$	$P=1.98\times10^{-7}$	-	-	-	$P=2.82\times10^{-8}$	$P=7.30\times10^{-138}$	-
**MROS (*n*=3,522)**	Healthy (*n*=388)	100.00%	75.92±5.55	25.84±3.18	125.39±15.75	66.80±9.28	6.09±0.97	15.84±8.09	57.19%/40.13%/2.68%
	Mild (*n*=1,168)	100.00%	76.17±5.53	26.54±3.59	126.43±16.03	67.42±9.29	6.17±0.93	18.66±8.02	58.98%/39.32%/1.69%
	Moderate (*n*=1,103)	100.00%	75.86±5.29	27.37±3.66	127.46±16.07	67.96±9.38	6.13±0.95	23.09±8.50	58.62%/39.56%/1.82%
	Severe (*n*=863)	100.00%	77.09±5.41	28.33±4.05	127.26±16.82	68.01±9.47	6.02±0.94	33.13±12.94	57.97%/40.78%/1.25%
	p-value	-	$P=4.90\times10^{-5}$	$P=1.39\times10^{-26}$	$P=0.40$	$P=0.23$	$P=0.02$	$P=2.85\times10^{-166}$	$P=0.92$
**CFS (*n*=576)**	Healthy (*n*=232)	33.19%	39.63±15.23	31.22±9.32	119.97±14.42	72.37±8.63	6.47±0.97	11.73±5.46	15.95%/57.33%/26.72%
	Mild (*n*=161)	42.86%	48.47±15.59	34.92±8.84	126.81±15.53	75.34±8.59	6.28±1.07	14.18±5.63	31.06%/44.10%/24.84%
	Moderate (*n*=101)	53.47%	52.82±14.59	34.75±7.31	126.41±14.73	76.22±8.44	6.12±1.13	18.91±8.24	35.64%/40.59%/23.76%
	Severe (*n*=82)	65.85%	51.85±14.81	39.80±8.63	133.12±17.38	78.33±10.51	6.02±0.92	31.34±12.68	24.39%/42.68%/32.93%
	p-value	$P=1.04\times10^{-6}$	$P=9.39\times10^{-15}$	$P=1.54\times10^{-15}$	$P=8.95\times10^{-12}$	$P=7.67\times10^{-7}$	$P=5.47\times10^{-4}$	$P=1.86\times10^{-43}$	$P=0.11$
**FDU-HSH (R) (*n*=327)**	Healthy (*n*=50)	28.00%	46.22±13.30	20.88±2.20	-	-	6.44±0.97	0.48±0.62	-
	Mild (*n*=116)	38.21%	53.45±11.25	23.52±2.85	-	-	6.19±0.96	3.95±1.82	-
	Moderate (*n*=68)	61.33%	56.25±11.64	24.47±2.62	-	-	6.18±1.02	9.38±3.86	-
	Severe (*n*=93)	83.33%	52.14±12.17	27.03±4.47	-	-	6.19±0.96	30.73±19.97	-
	p-value	$P=1.47\times10^{-13}$	$P=9.33\times10^{-5}$	$P=2.36\times10^{-22}$	-	-	$P=0.55$	$P=2.85\times10^{-58}$	-
**FDU-HSH (P) (*n*=265)**	Healthy (*n*=46)	47.83%	40.22±12.43	22.02±3.02	-	-	6.37±0.94	0.76±0.91	-
	Mild (*n*=75)	53.33%	51.77±12.40	23.56±3.05	-	-	6.26±0.83	4.47±2.16	-
	Moderate (*n*=68)	57.35%	55.97±12.29	24.51±3.16	-	-	6.19±0.90	9.10±3.90	-
	Severe (*n*=76)	81.58%	52.93±11.60	27.75±6.16	-	-	5.98±1.01	30.47±18.05	-
	p-value	$P=2.60\times10^{-4}$	$P=1.74\times10^{-8}$	$P=1.32\times10^{-15}$	-	-	$P=0.13$	$P=1.84\times10^{-44}$	-

Supplementary Table 2 | Performance of the All-Sub SHHS model (trained on *n*=5,255 subjects) is evaluated across five external test cohorts using various channel configurations (Flow, Chest, SpO_2, and Gold). For each channel, the mean absolute error (MAE, reported as mean ± SD) is provided for both four-level overall predictions and specific SA severity groups (healthy: AHI <5, mild: 5 ≤AHI <15, moderate: 15 ≤AHI <30, severe: AHI ≥30). Furthermore, overall accuracy (ACC), specificity (SPE), and the F1 score for each severity class, along with the Macro F1 score ($F1_{Macro}$), are presented to assess classification performance. The "Gold" configuration represents the integration of Flow, Chest, and SpO_2 channels.

Cohort	Subject distribution	Flow			Chest			SpO_2			Gold		
		SPE	F1	MAE	SPE	F1	MAE	SPE	F1	MAE	SPE	F1	MAE
**MESA (*n*=1,846)**	Healthy (*n*=187)	0.998	0.109	10.44±6.44	0.866	0.457	2.64±2.79	0.947	0.694	1.52±1.22	0.920	0.703	1.11±0.95
	Mild (*n*=571)	0.861	0.351	11.13±8.20	0.667	0.513	4.29±3.27	0.837	0.736	2.65±2.29	0.849	0.703	2.85±2.24
	Moderate (*n*=559)	0.660	0.398	10.72±7.95	0.786	0.388	8.95±5.75	0.888	0.698	4.60±3.55	0.890	0.681	4.57±3.52
	Severe (*n*=529)	0.743	0.704	11.37±10.00	0.976	0.580	19.33±14.56	0.985	0.854	7.65±7.34	0.986	0.860	8.31±8.24
	Overall (*n*=1,846)	ACC=0.482 $F1_{Macro}$=0.390		11.00±8.53	ACC=0.484 $F1_{Macro}$=0.485		9.84±10.75	ACC=0.751 $F1_{Macro}$=0.746		4.56±5.08	ACC=0.738 $F1_{Macro}$=0.737		4.76±5.56
**MROS (*n*=3,522)**	Healthy (*n*=388)	0.985	0.317	5.47±4.75	0.957	0.507	3.69±3.71	0.980	0.648	2.18±1.64	0.963	0.752	1.38±1.30
	Mild (*n*=1,168)	0.779	0.577	5.27±4.50	0.808	0.611	4.58±4.22	0.859	0.772	2.40±2.18	0.898	0.786	2.23±1.93
	Moderate (*n*=1,103)	0.744	0.552	6.55±5.14	0.793	0.565	6.75±5.59	0.885	0.767	3.30±2.89	0.905	0.776	3.32±2.74
	Severe (*n*=863)	0.908	0.749	10.07±8.79	0.913	0.764	9.37±8.52	0.968	0.883	5.28±4.87	0.962	0.891	5.28±4.73
	Overall (*n*=3,522)	ACC=0.593 $F1_{Macro}$=0.549		6.87±6.32	ACC=0.625 $F1_{Macro}$=0.612		6.34±6.25	ACC=0.785 $F1_{Macro}$=0.768		3.36±3.41	ACC=0.805 $F1_{Macro}$=0.801		3.23±3.32
**CFS (*n*=576)**	Healthy (*n*=232)	0.965	0.368	5.37±3.52	0.849	0.667	3.29±3.42	0.956	0.846	1.75±1.14	0.907	0.884	0.98±0.91
	Mild (*n*=161)	0.492	0.500	4.44±4.71	0.648	0.484	4.25±3.87	0.848	0.682	2.71±2.99	0.887	0.724	2.38±2.42
	Moderate (*n*=101)	0.893	0.510	7.77±6.54	0.895	0.385	9.51±6.83	0.918	0.710	4.47±6.95	0.949	0.705	4.97±6.06
	Severe (*n*=82)	0.964	0.693	17.43±11.55	0.976	0.597	21.46±14.78	0.974	0.862	9.02±8.90	0.980	0.850	10.57±8.29
	Overall (*n*=576)	ACC=0.493 $F1_{Macro}$=0.518		7.25±7.47	ACC=0.549 $F1_{Macro}$=0.533		7.24±9.28	ACC=0.774 $F1_{Macro}$=0.775		3.53±5.33	ACC=0.804 $F1_{Macro}$=0.791		3.44±5.34
**FDU-HSH (Retrospective) (*n*=327)**	Healthy (*n*=50)	-	-	-	-	-	-	0.895	0.746	1.40±0.74	-	-	-
	Mild (*n*=116)	-	-	-	-	-	-	0.910	0.750	2.51±2.37	-	-	-
	Moderate (*n*=68)	-	-	-	-	-	-	0.927	0.711	3.76±3.17	-	-	-
	Severe (*n*=93)	-	-	-	-	-	-	0.983	0.904	8.36±7.57	-	-	-
	Overall (*n*=327)	-		-	-		-	ACC = 0.783 $F1_{Macro}$=0.778		4.27±5.24	-		-
**FDU-HSH (Prospective) (*n*=265)**	Healthy (*n*=46)	-	-	-	-	-	-	0.895	0.800	1.47±0.53	-	-	-
	Mild (*n*=75)	-	-	-	-	-	-	0.874	0.697	2.86±2.15	-	-	-
	Moderate (*n*=68)	-	-	-	-	-	-	0.959	0.733	5.14±4.09	-	-	-
	Severe (*n*=76)	-	-	-	-	-	-	0.995	0.923	12.0±10.1	-	-	-
	Overall (*n*=265)	-		-	-		-	ACC = 0.789 $F1_{Macro}$=0.788		5.83±7.16	-		-

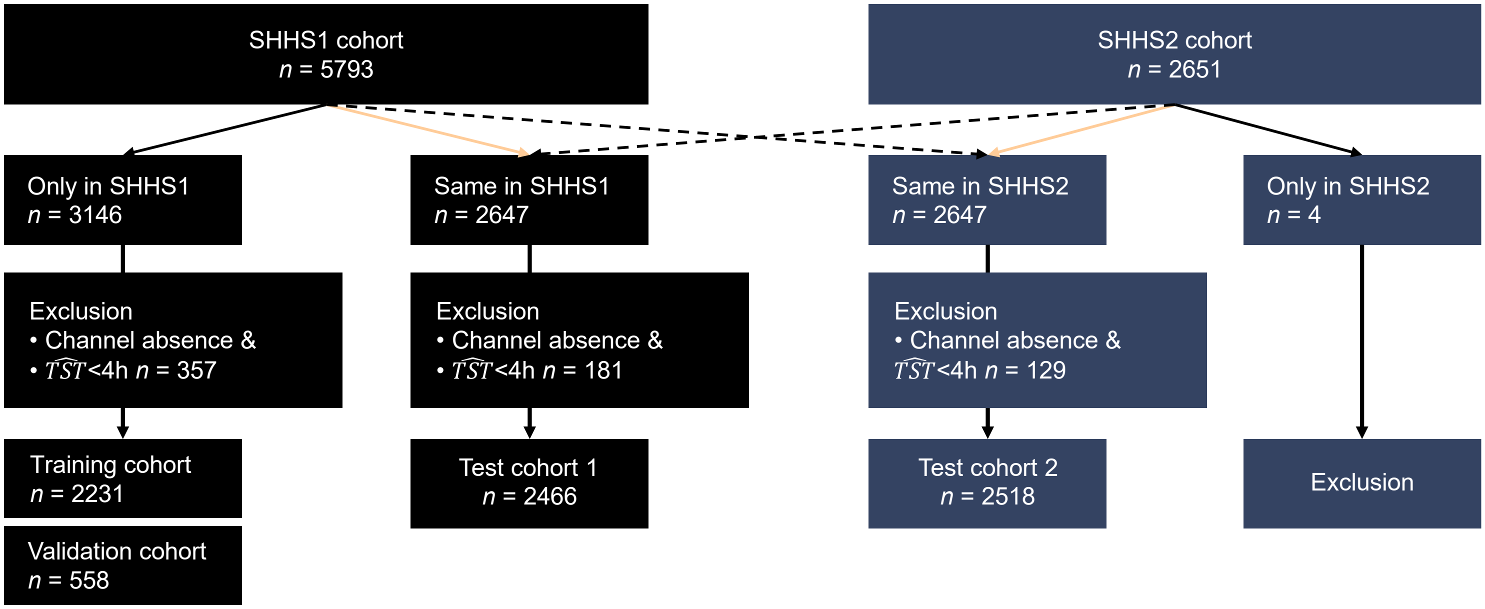

SHHS1 train cohort	SHHS1 test cohort 1	SHHS2 test cohort 2
n = 2789	*n* = 2466	*n* = 2518
Gender	Gender	Gender
Male: 1371<br>Female: 1418	Male: 1138<br>Female: 1328	Male: 1156<br>Female: 1362
Age	Age	Age
Mean: 63.3<br>Med: 63.0<br>High: 90.0<br>Low: 39.0	Mean: 62.1<br>Med: 62.0<br>High: 90.0<br>Low: 44.0	Mean: 67.3<br>Med: 67.0<br>High: 90.0<br>Low: 44.0
BMI	BMI	BMI
Mean: 28.1<br>Med: 27.4<br>High: 50.0<br>Low: 18.0	Mean: 28.2<br>Med: 27.6<br>High: 50.0<br>Low: 18.0	Mean: 28.4<br>Med: 27.8<br>High: 50.0<br>Low: 18.0
AHI	AHI	AHI
Mean: 18.7<br>Med: 13.3<br>High: 121.3<br>Low: 0.0	Mean: 16.8<br>Med: 12.6<br>High: 126.0<br>Low: 0.2	Mean: 18.3<br>Med: 13.6<br>High: 110.3<br>Low: 0.0
Sleep duration (*h*)	Sleep duration (*h*)	Sleep duration (*h*)
Mean: 6.05	Mean: 6.25	Mean: 6.37

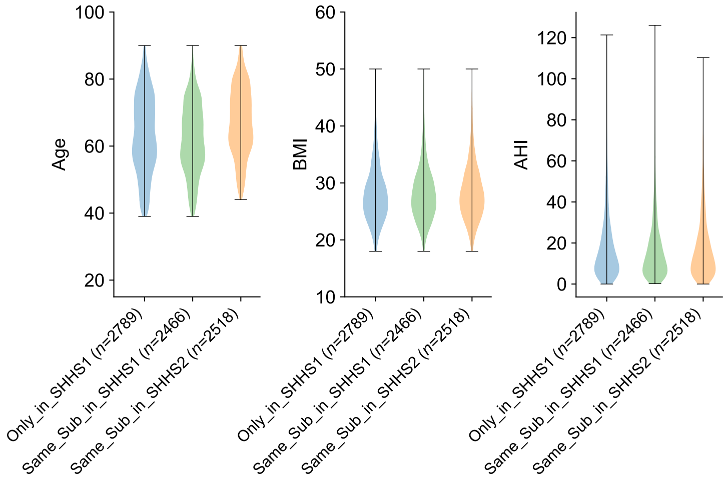

Supplementary Fig. 1 | Data partition scheme and cohort characteristics for temporal stability analysis based on the Non-overlap SHHS model (trained on *n*=2,789 subjects). (top) Flow diagram illustrating the data partition strategy for SHHS1 (*n*=5,793) and SHHS2 (*n*=2,651) cohorts. SHHS1 was split into training (*n*=2,231), validation (*n*=558), and test cohort 1 (*n*=2,466) after applying exclusion criteria. SHHS2 formed test cohort 2 (*n*=2,518) after applying exclusion criteria. The overlapping subjects between SHHS1 and SHHS2 (*n*=2,647) were excluded to ensure no overlap and were instead used for temporal stability assessment. (bottom) Demographic and clinical characteristics of the training cohort, test cohort 1, and test cohort 2, including gender distribution, age, BMI, AHI, and sleep duration statistics. The violin plots display the age, BMI, and AHI distributions across the three cohorts.

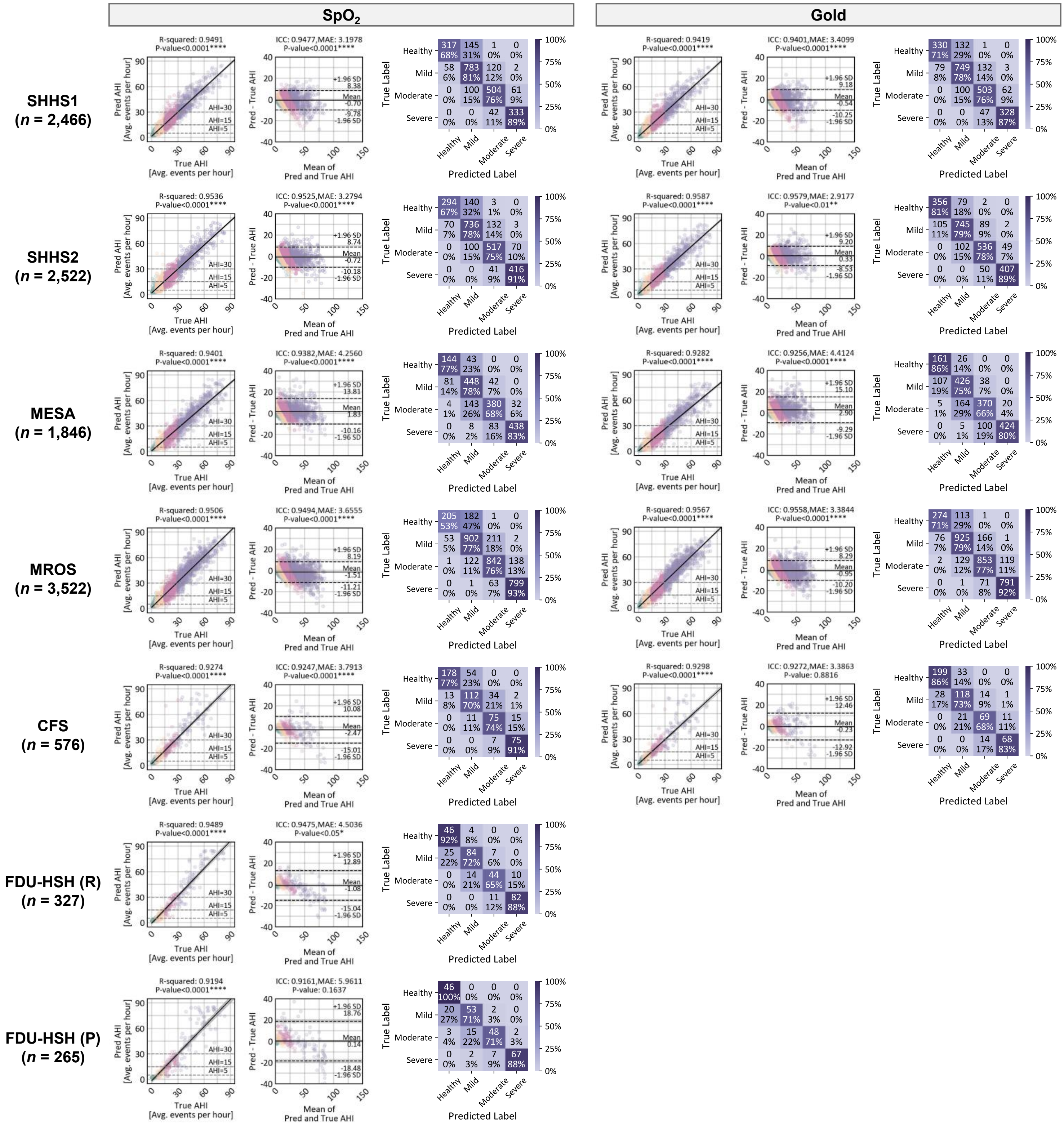

Supplementary Fig. 2 | Performance of the Non-overlap SHHS model (trained on *n*=2,789 subjects) on AHI regression and four-level SA severity classification across test cohorts using SpO_2 and Gold channels. The figure illustrates the model's performance across seven test cohorts for SpO_2, including SHHS1, SHHS2, MESA, MROS, CFS, FDU-HSH (R), and FDU-HSH (P) and five test cohorts for Gold, including SHHS1, SHHS2, MESA, MROS, and CFS. For each cohort and channel, the results are presented as scatter plots, Bland-Altman plots, and confusion matrices.

Supplementary Table 3 | Performance of the Non-overlap SHHS model (trained on *n*=2,789 subjects) for AHI regression and four-level SA severity classification across test cohorts using various channel configurations (Flow, Chest, SpO_2, and Gold). Metrics include SEN, SPE, PPV, F1 score, $F1_{Macro}$ and MAE (mean ± SD).

Cohort	Subject distribution	Flow			Chest			SpO_2			Gold		
		SPE	F1	MAE	SPE	F1	MAE	SPE	F1	MAE	SPE	F1	MAE
**SHHS1 (*n*=2,466)**	Healthy (*n*=463)	0.971	0.426	3.83±2.82	0.956	0.479	4.22±4.31	0.971	0.757	1.81±1.58	0.961	0.757	1.67±1.71
	Mild (*n*=963)	0.667	0.647	3.78±3.35	0.683	0.652	3.84±3.29	0.837	0.787	2.43±2.40	0.846	0.771	2.67±2.60
	Moderate (*n*=665)	0.829	0.613	5.54±4.41	0.830	0.598	5.74±4.57	0.909	0.757	3.73±3.53	0.900	0.746	4.05±3.67
	Severe (*n*=375)	0.967	0.741	10.17±8.53	0.971	0.728	10.64±8.73	0.970	0.864	5.96±5.00	0.969	0.854	6.32±5.27
	Overall (*n*=2,466)	ACC=0.621<br>$F1_{Macro}$=0.607		5.23±5.21	ACC=0.622<br>$F1_{Macro}$=0.614		5.46±5.51	ACC=0.785<br>$F1_{Macro}$=0.791		3.20±3.42	ACC=0.775<br>$F1_{Macro}$=0.782		3.41±3.64
**SHHS2 (*n*=2,522)**	Healthy (*n*=437)	0.959	0.428	4.03±3.03	0.948	0.492	4.04±3.84	0.966	0.734	2.02±2.00	0.950	0.793	1.30±1.54
	Mild (*n*=941)	0.673	0.642	3.69±3.23	0.715	0.625	4.05±3.38	0.848	0.768	2.66±3.07	0.886	0.798	2.25±2.39
	Moderate (*n*=687)	0.829	0.575	5.94±4.55	0.802	0.544	6.17±4.89	0.904	0.749	3.48±3.02	0.923	0.786	2.97±2.50
	Severe (*n*=457)	0.969	0.741	11.46±9.62	0.952	0.696	12.04±10.83	0.965	0.879	5.46±5.29	0.975	0.890	5.75±5.63
	Overall (*n*=2,522)	ACC=0.611<br>$F1_{Macro}$=0.596		5.77±5.99	ACC=0.595<br>$F1_{Macro}$=0.589		6.07±6.58	ACC=0.778<br>$F1_{Macro}$=0.783		3.28±3.61	ACC=0.810<br>$F1_{Macro}$=0.817		2.92±3.47
**MESA (*n*=1,846)**	Healthy (*n*=187)	0.999	0.091	10.10±6.08	0.861	0.460	2.49±2.67	0.949	0.692	1.58±1.24	0.932	0.700	1.05±0.94
	Mild (*n*=571)	0.849	0.373	10.62±8.06	0.658	0.510	4.21±3.17	0.848	0.739	2.68±2.41	0.847	0.715	2.67±2.17
	Moderate (*n*=559)	0.664	0.404	10.41±7.85	0.794	0.395	9.14±5.73	0.903	0.714	4.42±3.59	0.893	0.694	4.40±3.41
	Severe (*n*=529)	0.758	0.708	11.57±10.13	0.983	0.569	19.86±14.66	0.976	0.877	6.73±6.87	0.985	0.872	7.49±7.88
	Overall (*n*=1,846)	ACC=0.488<br>$F1_{Macro}$=0.394		10.78±8.50	ACC=0.483<br>$F1_{Macro}$=0.484		10.01±10.94	ACC=0.764<br>$F1_{Macro}$=0.756		4.26±4.76	ACC=0.748<br>$F1_{Macro}$=0.745		4.41±5.26
**MROS (*n*=3,522)**	Healthy (*n*=388)	0.988	0.290	5.37±4.49	0.954	0.518	3.52±3.59	0.983	0.634	2.28±1.69	0.975	0.741	1.58±1.47
	Mild (*n*=1,168)	0.763	0.592	4.92±4.24	0.801	0.624	4.35±3.97	0.870	0.760	2.63±2.37	0.897	0.792	2.31±2.00
	Moderate (*n*=1,103)	0.752	0.562	6.44±5.06	0.800	0.579	6.54±5.40	0.886	0.759	3.63±3.22	0.902	0.778	3.43±2.89
	Severe (*n*=863)	0.920	0.754	10.34±8.91	0.927	0.769	9.33±8.61	0.947	0.887	5.70±5.11	0.955	0.892	5.59±4.77
	Overall (*n*=3,522)	ACC=0.601<br>$F1_{Macro}$=0.550		6.77±6.34	ACC=0.634<br>$F1_{Macro}$=0.623		6.17±6.19	ACC=0.780<br>$F1_{Macro}$=0.760		3.66±3.66	ACC=0.807<br>$F1_{Macro}$=0.800		3.38±3.42
**CFS (*n*=576)**	Healthy (*n*=232)	0.980	0.311	5.33±3.25	0.954	0.518	3.15±3.22	0.962	0.842	1.84±1.16	0.919	0.867	1.03±0.96
	Mild (*n*=161)	0.465	0.510	4.22±4.52	0.801	0.624	4.15±3.59	0.843	0.663	2.95±3.26	0.870	0.709	2.31±2.43
	Moderate (*n*=101)	0.891	0.507	7.75±6.55	0.800	0.579	9.48±6.69	0.914	0.691	4.89±7.36	0.941	0.697	4.90±6.45
	Severe (*n*=82)	0.968	0.693	17.94±11.95	0.927	0.769	21.96±14.95	0.966	0.862	9.61±9.49	0.976	0.840	10.28±8.94
	Overall (*n*=576)	ACC=0.484<br>$F1_{Macro}$=0.505		7.24±7.63	ACC=0.552<br>$F1_{Macro}$=0.532		7.22±9.41	ACC=0.764<br>$F1_{Macro}$=0.764		3.79±5.71	ACC=0.788<br>$F1_{Macro}$=0.778		3.39±5.52
**FDU-HSH (R) (*n*=327)**	Healthy (*n*=50)	-	-	-	-	-	-	0.910	0.760	1.63±0.79	-	-	-
	Mild (*n*=116)	-	-	-	-	-	-	0.915	0.771	2.41±2.37	-	-	-
	Moderate (*n*=68)	-	-	-	-	-	-	0.931	0.677	3.89±3.14	-	-	-
	Severe (*n*=93)	-	-	-	-	-	-	0.957	0.886	9.11±8.04	-	-	-
	Overall (*n*=327)	-		-	-		-	ACC=0.783<br>$F1_{Macro}$=0.774		3.28±3.62	-		-
**FDU-HSH (P) (*n*=265)**	Healthy (*n*=46)	-	-	-	-	-	-	0.895	0.800	1.60±0.57	-	-	-
	Mild (*n*=75)	-	-	-	-	-	-	0.911	0.731	2.61±2.14	-	-	-

	Moderate (*n*=68)	-	-	-	-	-	-	0.954	0.768	4.84±3.95	-	-	-
	Severe (*n*=76)	-	-	-	-	-	-	0.989	0.924	12.91±9.95	-	-	-
	Overall (*n*=265)	-		-	-		-	ACC=0.808<br>$F1_{Macro}$=0.806		5.96±7.37	-		-

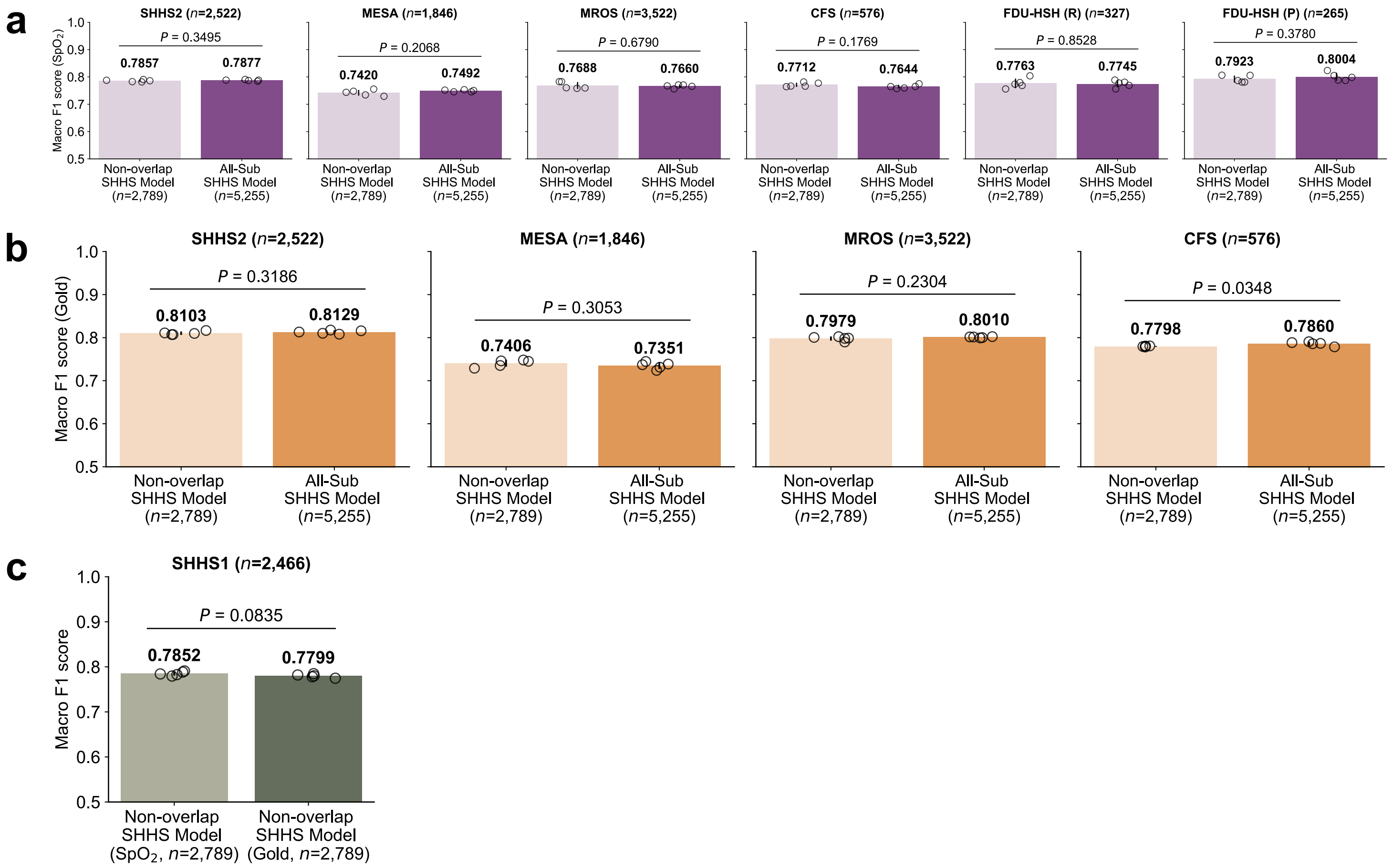

Supplementary Fig. 3 | Comparison of model performance between Non-overlap SHHS and All-Sub SHHS models using SpO_2 and Gold channels across test cohorts. (a) Macro F1 scores for the SpO_2 channel across six test cohorts (SHHS2, MESA, MROS, CFS, FDU-HSH (R), and FDU-HSH (P)). Performance is compared between the Non-overlap SHHS model (*n*=2,789 subjects) and the All-Sub SHHS model (*n*=5,255 subjects) with statistical significance assessed using a two-sided t-test. (b) Macro F1 scores for the Gold channel across four test cohorts (SHHS2, MESA, MROS, and CFS). Statistical significance is evaluated similarly. (c) Comparison of Macro F1 scores for SpO_2 and Gold channels on SHHS1, highlighting the differences in performance between the two channels using the Non-overlap SHHS model. For all panels, the mean ± SD are shown, with statistical significance indicated by *p*-values. No significant differences are observed in most cohorts, except for CFS (*P*=0.0348 in panel b).

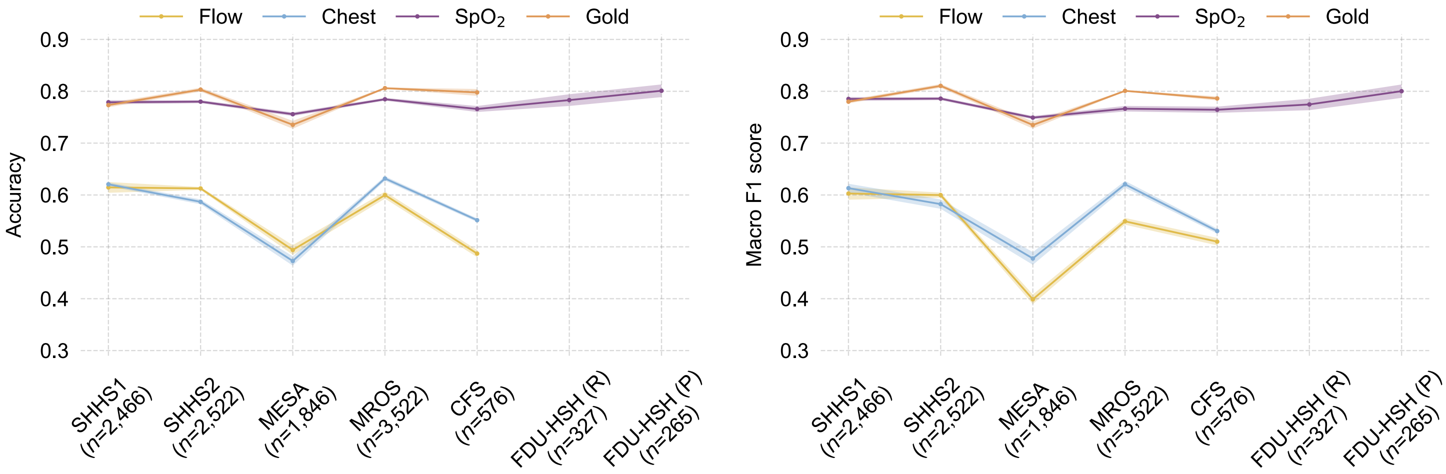

Channel	Metric (Mean, 95%CI)	SHHS1 (*n*=2,466)	SHHS2 (*n*=2,522)	MESA (*n*=2,522)	MROS (*n*=3,522)	CFS (*n*=576)	FDU-HSH (R) (*n*=327)	FDU-HSH (P) (*n*=265)
Flow	ACC	0.615 (0.604,0.625)	0.613 (0.610,0.615)	0.494 (0.485,0.503)	0.600 (0.593,0.607)	0.487 (0.480,0.494)	-	-
	$F1_{Macro}$	0.603 (0.591,0.615)	0.600 (0.595,0.605)	0.399 (0.389,0.408)	0.549 (0.543,0.556)	0.510 (0.503,0.517)	-	-
Chest	ACC	0.621 (0.617,0.624)	0.587 (0.582,0.591)	0.473 (0.464,0.482)	0.632 (0.628,0.636)	0.551 (0.548,0.555)	-	-
	$F1_{Macro}$	0.613 (0.605,0.622)	0.582 (0.573,0.591)	0.478 (0.465,0.490)	0.621 (0.614,0.627)	0.530 (0.526,0.535)	-	-
SpO_2	ACC	0.779 (0.775,0.783)	0.780 (0.777,0.783)	0.756 (0.752,0.759)	0.785 (0.782,0.787)	0.766 (0.760,0.772)	0.783 (0.772,0.794)	0.801 (0.789,0.813)
	$F1_{Macro}$	0.785 (0.781,0.789)	0.786 (0.783,0.789)	0.749 (0.746,0.752)	0.766 (0.761,0.772)	0.764 (0.758,0.770)	0.775 (0.764,0.786)	0.800 (0.787,0.813)
Gold	ACC	0.773 (0.769,0.778)	0.803 (0.800,0.807)	0.735 (0.728,0.743)	0.806 (0.805,0.807)	0.798 (0.791,0.804)	-	-
	$F1_{Macro}$	0.780 (0.777,0.783)	0.810 (0.807,0.814)	0.735 (0.728,0.742)	0.801 (0.800,0.802)	0.786 (0.782,0.790)	-	-

Supplementary Fig. 4 | Performance comparison of different channel configurations for four-level SA severity classification across test cohorts (with 95% CI). The left panel shows classification accuracy and the right panel shows Macro F1 scores across different cohorts. SpO_2 and Gold configurations consistently demonstrate superior performance in both classification accuracy (Mean accuracy: SpO_2 0.756-0.785, Gold 0.735-0.806) and Macro F1 scores (Mean Macro F1 scores: SpO_2 0.749-0.786, Gold 0.735-0.810) compared to Flow and Chest configurations. To avoid bias, results for SHHS1 (*n*=2,466 subjects) and SHHS2 (*n*=2,522 subjects) are reported based on the Non-overlap SHHS model, which excludes these cohorts during training. In contrast, results for MESA (*n*=1,846 subjects), MROS (*n*=3,522 subjects), CFS (*n*=576 subjects), FDU-HSH retrospective (R) cohort (*n*=327 subjects), and FDU-HSH prospective (P) cohort (*n*=265 subjects) are reported based on the All-Sub SHHS model, which fully utilizes the available training data. While SpO_2 and Gold configurations exhibit strong and consistent performance across most cohorts, performance shows a slight decline on MESA. Nevertheless, single-channel SpO_2 monitoring achieves comparable performance to the Gold standard configuration, suggesting its potential for a simplified home-based setting.

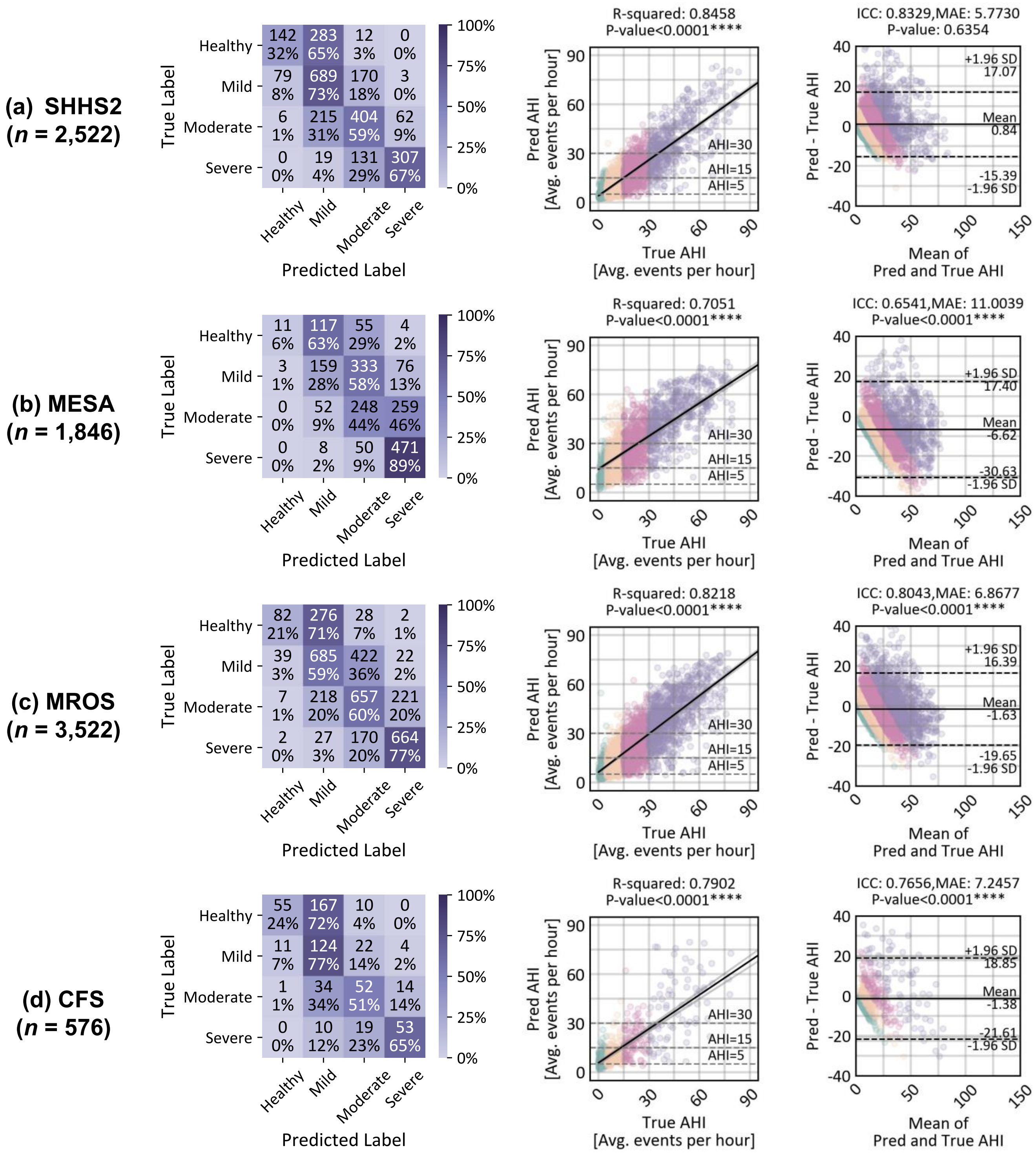

Supplementary Fig. 5 | Performance evaluation of the AIX system under single-channel (Flow) workflow on four external test cohorts: SHHS2 (a) using the Non-overlap model, and MESA (b), MROS (c), CFS (d) using the All-Sub SHHS model. Each test cohort presents the four-level SA classification confusion matrix, correlation scatter plots for AHI regression, and Bland-Altman plots. The R^2 values are provided along with the diagonal line representing the linear regression model and the two-sided t-test *p*-values corresponding to the 95% CIs. The Bland-Altman plots show the comparison between manually measured AHI and predicted AHI, with error lines positioned at ±1.96 SD. *p*-values are calculated using the two-sided Wilcoxon signed-rank test, and ICCs are provided, along with the MAE of predicted AHI for all subjects. Green, yellow, pink, and purple scatter points represent healthy individuals, mild SA, moderate SA, and severe SA patients, respectively.

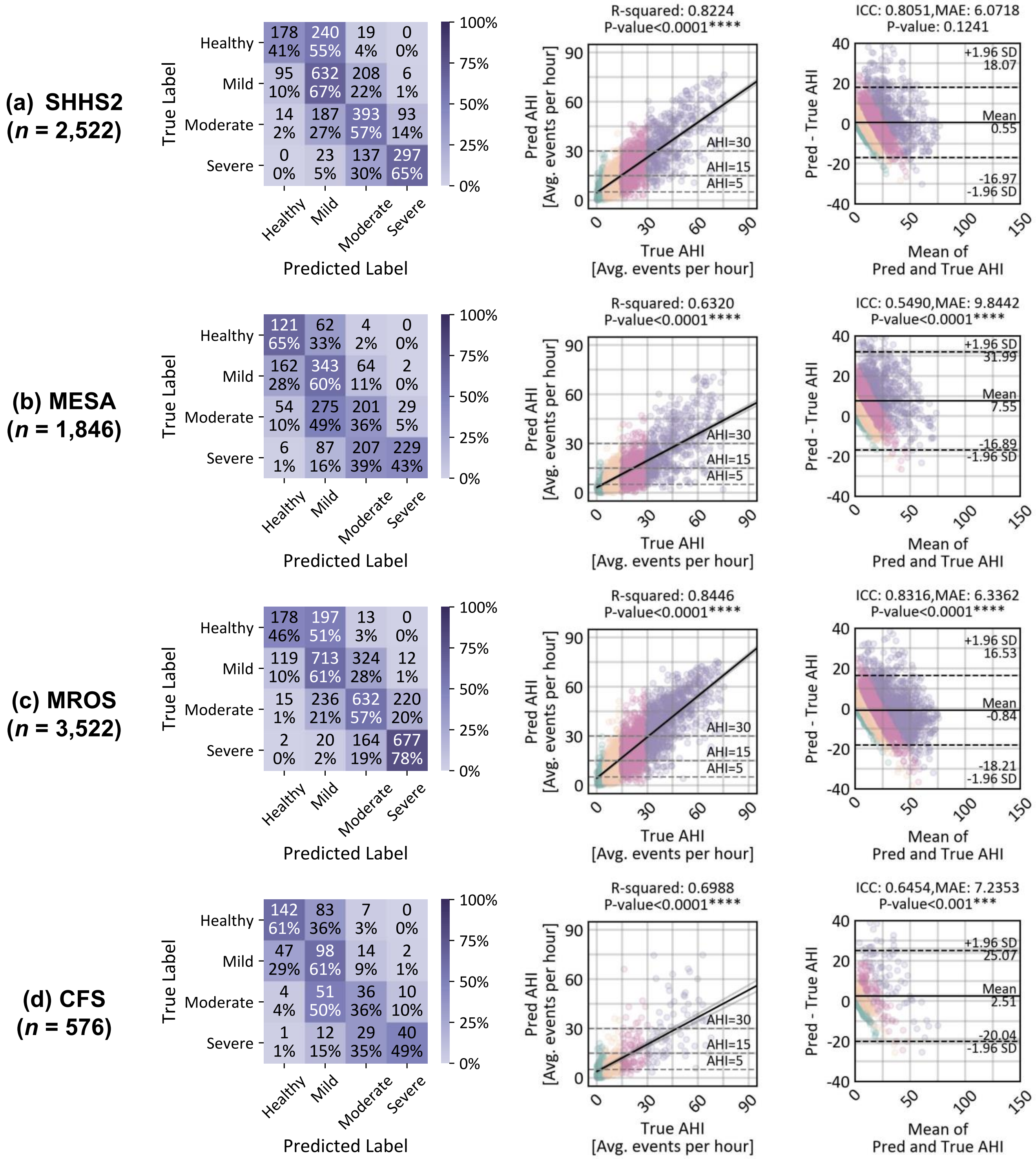

Supplementary Fig. 6 | Performance evaluation of the AIX system under single-channel (Chest) workflow on four external test cohorts: SHHS2 (a) using the Non-overlap model, and MESA (b), MROS (c), CFS (d) using the All-Sub SHHS model. Each test cohort presents the four-level SA classification confusion matrix, correlation scatter plots for AHI regression, and Bland-Altman plots. The R^2 values are provided along with the diagonal line representing the linear regression model and the two-sided t-test *p*-values corresponding to the 95% CIs. The Bland-Altman plots show the comparison between manually measured AHI and predicted AHI, with error lines positioned at ±1.96 SD. *p*-values are calculated using the two-sided Wilcoxon signed-rank test, and ICCs are provided, along with the MAE of predicted AHI for all patients. Green, yellow, pink, and purple scatter points represent healthy individuals, mild SA, moderate SA, and severe SA patients, respectively.

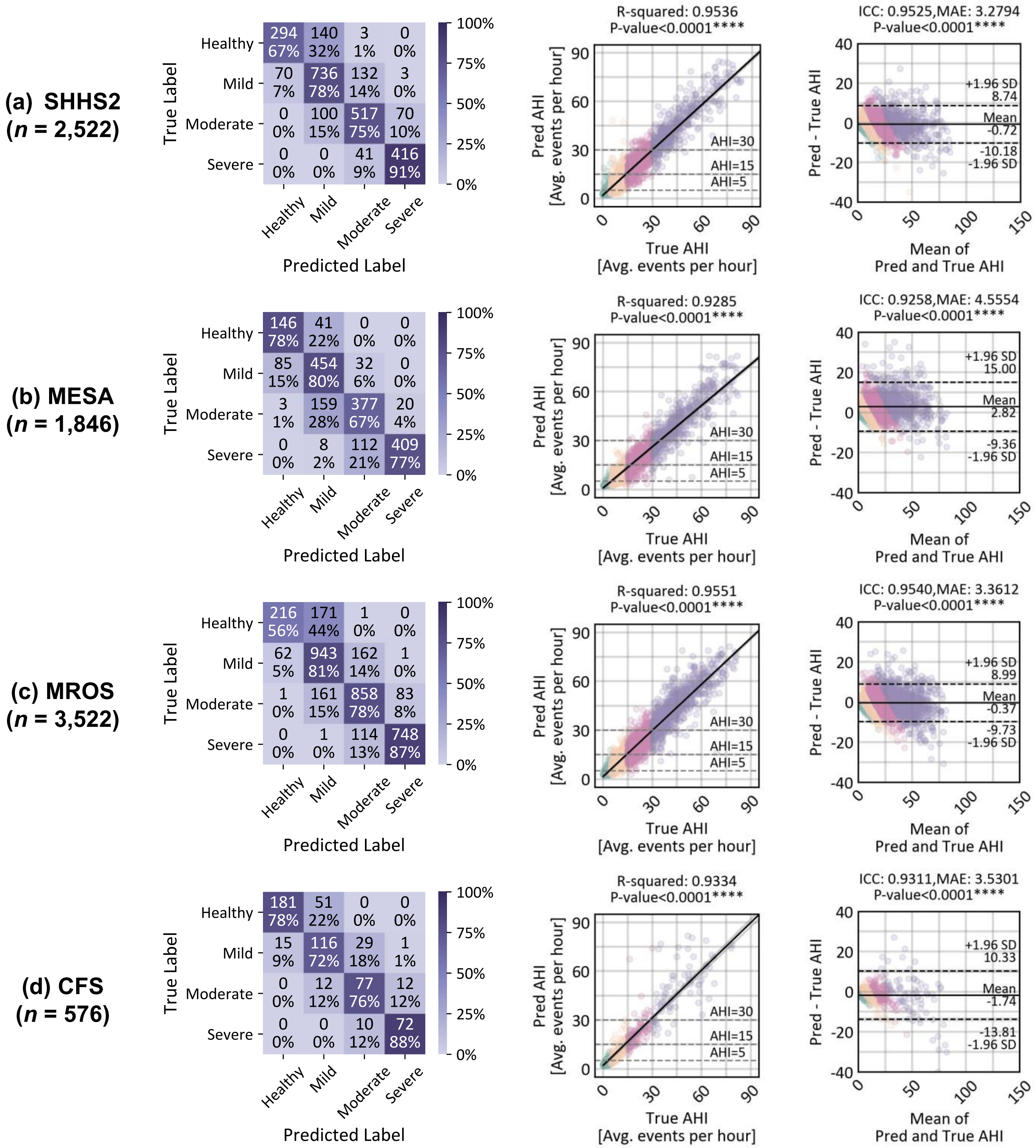

Supplementary Fig. 7 | Performance evaluation of the AIX system under single-channel (SpO_2) workflow on four external test cohorts: SHHS2 (a) using the Non-overlap model, and MESA (b), MROS (c), CFS (d) using the All-Sub SHHS model. Each test cohort presents the four-level SA classification confusion matrix, correlation scatter plots for AHI regression, and Bland-Altman plots. The R^2 values are provided along with the diagonal line representing the linear regression model and the two-sided t-test *p*-values corresponding to the 95% CIs. The Bland-Altman plots show the comparison between manually measured AHI and predicted AHI, with error lines positioned at ±1.96 SD. *p*-values are calculated using the two-sided Wilcoxon signed-rank test, and ICCs are provided, along with the MAE of predicted AHI for all patients. Green, yellow, pink, and purple scatter points represent healthy individuals, mild SA, moderate SA, and severe SA patients, respectively.

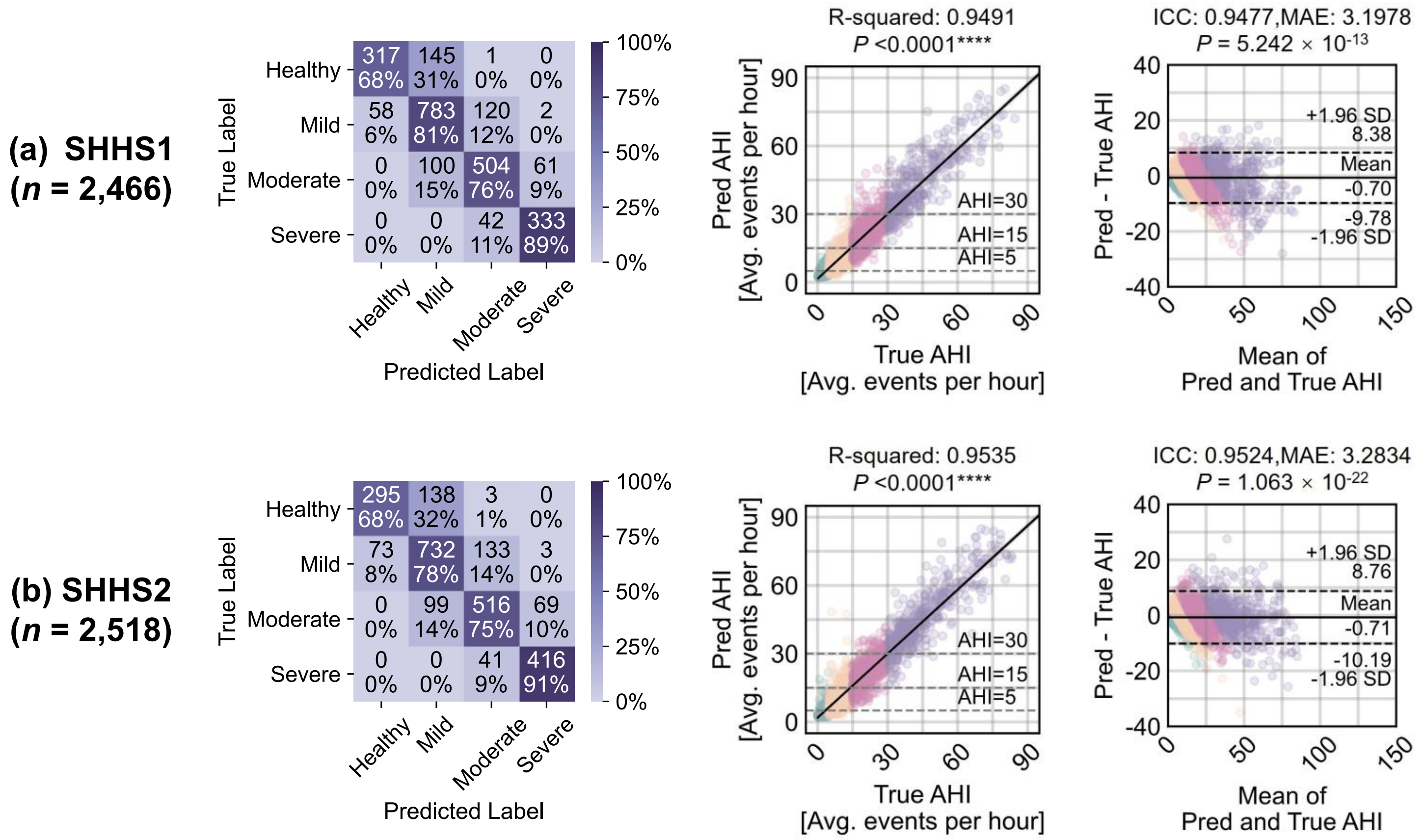

Supplementary Fig. 8 | Temporal performance comparison between SHHS1 and SHHS2 cohorts using single-channel SpO_2. (a) Model performance on SHHS1 test cohort (*n*=2,466 subjects). Left: confusion matrix showing the classification performance across four severity categories (numbers indicate absolute counts and percentages). Middle: scatter plot showing the correlation between predicted and true AHI values (R^2=0.9491, P<0.0001). Right: Bland-Altman plot showing the agreement between predicted and true AHI values (mean difference=-0.70, 95% limits of agreement: -9.78 to +8.38). (b) Model performance on SHHS2 test cohort (*n*=2,518 subjects). Left: confusion matrix for severity classification. Middle: scatter plot showing the correlation between predicted and true AHI values (R^2=0.9535, P<0.0001). Right: Bland-Altman analysis with mean difference=-0.71 and 95% limits of agreement from -10.19 to +8.76.

Supplementary Table 4 | Performance comparison between SHHS1 and SHHS2 cohorts (single-channel SpO_2). Detailed performance metrics (ACC, SEN, SPE, PPV, F1, $F1_{Macro}$, and MAE) across four severity categories and overall performance for SHHS1 (*n*=2,466 subjects) and SHHS2 (*n*=2,518 subjects) test cohorts. Data are presented as mean ± SD for MAE.

**Cohort**	**Patient distribution**	**Performance**				
		**SEN**	**SPE**	**PPV**	**F1**	**MAE**
**SHHS1 (*n*=2,466)**	Healthy (*n*=463)	0.685	0.971	0.845	0.757	1.81±1.58
	Mild (*n*=963)	0.813	0.837	0.762	0.787	2.43±2.40
	Moderate (*n*=665)	0.758	0.909	0.756	0.757	3.73±3.53
	Severe (*n*=375)	0.888	0.970	0.841	0.864	5.96±5.00
	Overall (*n*=2,466)	ACC=0.785, $F1_{Macro}$=0.791				3.20±3.42
**SHHS2 (*n*=2,518)**	Healthy (*n*=436)	0.677	0.965	0.802	0.734	2.02±2.00
	Mild (*n*=941)	0.778	0.850	0.755	0.766	2.66±3.07
	Moderate (*n*=684)	0.754	0.903	0.745	0.749	3.49±3.03
	Severe (*n*=457)	0.910	0.965	0.852	0.880	5.46±5.29
	Overall (*n*=2,518)	ACC=0.778, $F1_{Macro}$=0.783				3.28±3.62

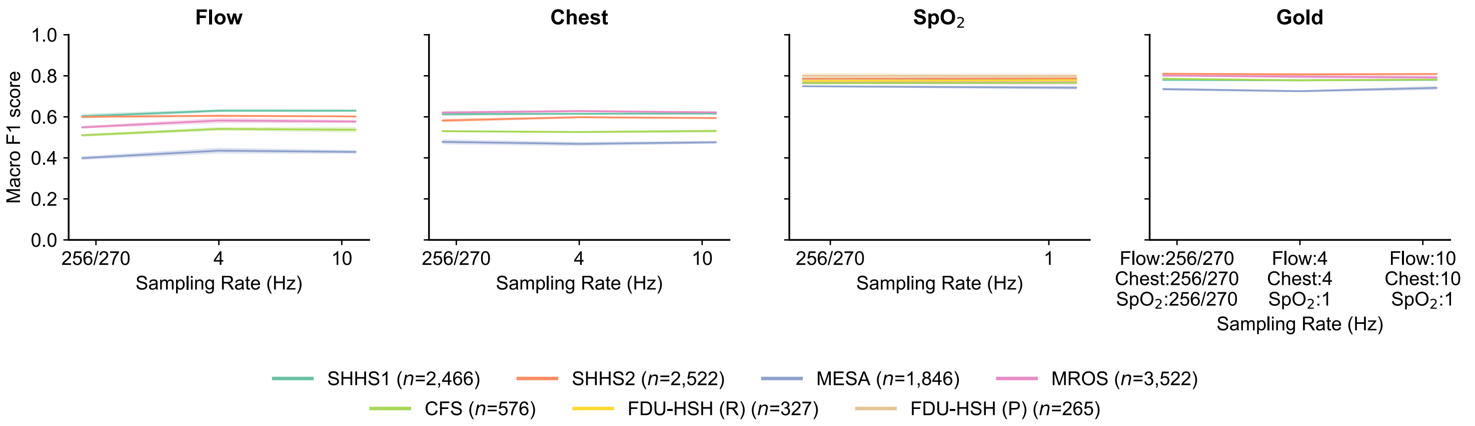

Channel	$F1_{Macro}$ (Mean, 95%CI)	SHHS1 (*n*=2,466)	SHHS2 (*n*=2,522)	MESA (*n*=2,522)	MROS (*n*=3,522)	CFS (*n*=576)	FDU-HSH (R) (*n*=327)	FDU-HSH (P) (*n*=265)
Flow	256/270 Hz	0.603 (0.591,0.615)	0.600 (0.595,0.605)	0.399 (0.389,0.408)	0.549 (0.543,0.556)	0.510 (0.503,0.517)	-	-
	4 Hz	0.630 (0.624,0.637)	0.605 (0.599,0.610)	0.435 (0.421,0.449)	0.582 (0.569,0.596)	0.541 (0.533,0.550)	-	-
	10 Hz	0.630 (0.624,0.636)	0.602 (0.600,0.605)	0.429 (0.420,0.438)	0.577 (0.569,0.584)	0.537 (0.524,0.550)	-	-
Chest	256/270 Hz	0.613 (0.605,0.622)	0.582 (0.573,0.592)	0.478 (0.465,0.490)	0.621 (0.614,0.627)	0.530 (0.526,0.535)	-	-
	4 Hz	0.615 (0.613,0.618)	0.598 (0.593,0.603)	0.468 (0.458,0.478)	0.628 (0.622,0.635)	0.526 (0.521,0.532)	-	-
	10 Hz	0.616 (0.611,0.621)	0.594 (0.591,0.598)	0.476 (0.469,0.484)	0.622 (0.620,0.624)	0.531 (0.524,0.538)	-	-
SpO_2	256/270 Hz	0.785 (0.781,0.789)	0.786 (0.783,0.789)	0.749 (0.746,0.752)	0.766 (0.761,0.772)	0.764 (0.758,0.770)	0.775 (0.764,0.786)	0.800 (0.787,0.813)
	1 Hz	0.786 (0.782,0.790)	0.787 (0.780,0.793)	0.742 (0.733,0.750)	0.768 (0.756,0.780)	0.766 (0.761,0.771)	0.779 (0.773,0.786)	0.799 (0.789,0.810)
Gold	256/270, 256/270, 256/270 Hz	0.780 (0.777,0.783)	0.810 (0.807,0.814)	0.735 (0.728,0.742)	0.801 (0.800,0.802)	0.786 (0.782,0.790)	-	-
	4,4,1 Hz	0.778 (0.775,0.781)	0.807 (0.801,0.812)	0.725 (0.720,0.731)	0.796 (0.787,0.805)	0.778 (0.772,0.784)	-	-
	10,10,1 Hz	0.780 (0.776,0.784)	0.809 (0.806,0.812)	0.741 (0.731,0.751)	0.792 (0.782,0.802)	0.782 (0.781,0.784)	-	-

Supplementary Fig. 9 | Performance evaluation of Macro F1 scores across different sampling rates and channel configurations. The figure includes results from four channels: Flow, Chest, SpO_2, and Gold. For each channel, Macro F1 scores are evaluated under multiple sampling rates (256/270 Hz, 4 Hz, 10 Hz for Flow and Chest; 256/270 Hz and 1 Hz for SpO_2; and combined configurations for Gold). The solid lines represent the mean Macro F1 scores, and the shaded regions indicate the 95% CI. The table below the plot provides the detailed mean Macro F1 scores and 95% CIs for each channel, sampling rate, and cohort. Results are shown for seven cohorts: SHHS1, SHHS2, MESA, MROS, CFS, FDU-HSH (R), and FDU-HSH (P).

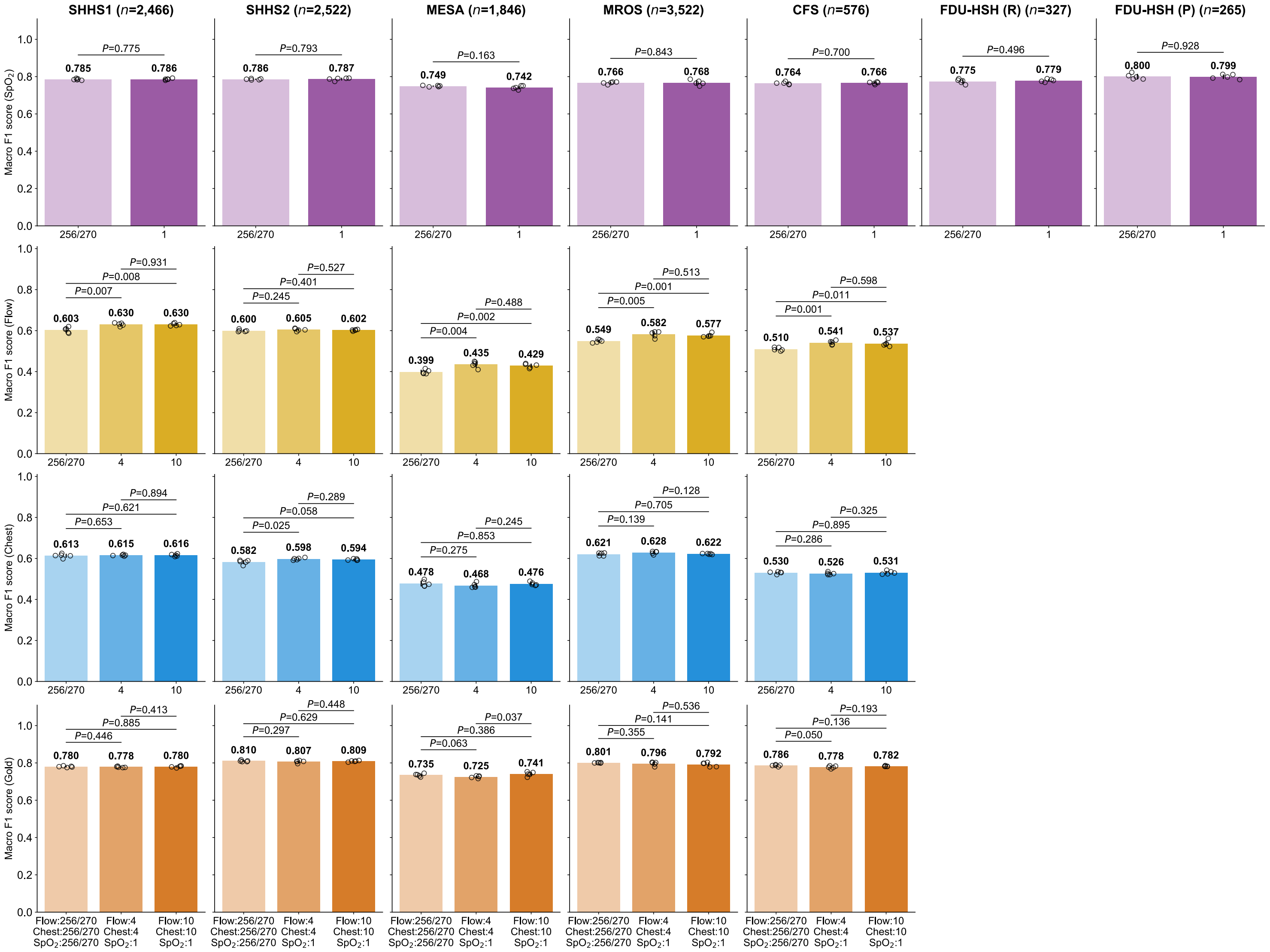

Supplementary Fig. 10 | Statistical evaluation of Macro F1 scores across different sampling rates and channel configurations. The bar plots present the mean Macro F1 scores under various sampling and channel configurations. The evaluated cohorts include SHHS1, SHHS2, MESA, MROS, CFS, FDU-HSH (R), and FDU-HSH (P). Error bars represent the SD obtained from five repeated experiments. The *p*-values, calculated using two-tailed paired t-tests, are displayed above the bars to assess statistical differences between configurations. Comparisons are made within each cohort for the same channel under different sampling rates.

Supplementary Table 5 | Ethnic composition across seven cohorts, including Asian, Black or African American, White, Hispanic, Multiple, and Other.

Cohort	Asian	Black or African American	White	Hispanic	Multiple	Other
**SHHS1 (*n*=5,255)**	-	8.45%	84.70%	-	-	6.85%
**SHHS2 (*n*=2,522)**	-	6.78%	86.99%	-	-	6.23%
**MESA (*n*=1,846)**	12.08%	26.60%	37.70%	23.62%	-	-
**MROS (*n*=3,522)**	2.95%	3.29%	90.60%	1.96%	-	1.21%
**CFS (*n*=576)**	-	55.38%	42.53%	-	2.08%	-
**FDU-HSH (R) (*n*=327)**	100.00%	-	-	-	-	-
**FDU-HSH (P) (*n*=265)**	100.00%	-	-	-	-	-

Supplementary Table 6 | AIX system performance is evaluated in both professional (Gold) and home-based (SpO2) settings, with four-level SA classification results analyzed across different ethnicities in the SHHS2, MESA, and CFS cohorts. Evaluation metrics include overall ACC, $F1_{Macro}$, R^2 value, and ICC.

Ethnicity (healthy/mild/moderate/severe)		SpO_2				Gold			
		ACC	$F1_{Macro}$	R^2	ICC	ACC	$F1_{Macro}$	R^2	ICC
SHHS2 (*n*=2,522)	Black or African American, *n*=171 (36/65/37/33)	0.760	0.759	0.951	0.949	0.801	0.805	0.962	0.961
	White, *n*=2,194 (362/820/605/407)	0.783	0.785	0.955	0.954	0.813	0.817	0.959	0.959
	Other, *n*=157 (39/56/45/17)	0.739	0.754	0.934	0.932	0.790	0.800	0.943	0.941
	Overall (437/941/687/457)	0.778	0.783	0.954	0.952	0.810	0.817	0.959	0.958
MESA (*n*=1,846)	Asian, *n*=223 (22/66/67/68)	0.731	0.727	0.950	0.949	0.722	0.719	0.949	0.948
	Black or African American, *n*=491 (60/161/142/128)	0.760	0.758	0.930	0.927	0.727	0.730	0.909	0.905
	White, *n*=696 (68/227/204/197)	0.741	0.736	0.916	0.912	0.740	0.739	0.905	0.900
	Hispanic, *n*=436 (37/117/146/136)	0.766	0.755	0.935	0.932	0.755	0.753	0.925	0.922
	Overall (187/571/559/529)	0.751	0.746	0.928	0.926	0.738	0.737	0.916	0.913
CFS (*n*=576)	Black or African American, *n*=319 (125/92/50/52)	0.781	0.779	0.948	0.946	0.803	0.781	0.946	0.944
	White, *n*=245 (100/65/51/29)	0.759	0.760	0.912	0.908	0.796	0.790	0.907	0.903
	Multiple, *n*=12 (7/4/0/1)	0.917	0.937	0.985	0.985	1.000	1.000	0.981	0.981
	Overall (232/161/101/82)	0.774	0.775	0.933	0.931	0.804	0.791	0.930	0.928

Supplementary Table 7 | Binary classification performance of the AIX system with AHI cutoff at 5, comparing AHI<5 versus AHI≥5 subjects in both professional (Gold) and home-based (SpO_2) settings. Performance metrics include ACC, $F1_{Macro}$, SEN, PPV, SPE across six external test cohorts.

Cohort	SpO_2					Gold				
	ACC	$F1_{Macro}$	SEN	PPV	SPE	ACC	$F1_{Macro}$	SEN	PPV	SPE
**SHHS2 (*n*=2,522)**	0.916	0.842	0.966	0.934	0.673	0.926	0.874	0.950	0.961	0.815
**MESA (*n*=1,846)**	0.930	0.827	0.947	0.975	0.781	0.921	0.829	0.920	0.991	0.925
**MROS (*n*=3,522)**	0.933	0.805	0.980	0.947	0.557	0.943	0.860	0.963	0.973	0.781
**CFS (*n*=576)**	0.885	0.877	0.956	0.866	0.780	0.905	0.901	0.907	0.931	0.901
**FDU-HSH (R) (*n*=327)**	0.902	0.843	0.895	0.988	0.940	-	-	-	-	-
**FDU-HSH (P) (*n*=265)**	0.913	0.872	0.895	1.000	1.000	-	-	-	-	-

Supplementary Table 8 | Binary classification performance of the AIX system with AHI cutoff at 15, comparing AHI<15 versus AHI≥15 subjects in both professional (Gold) and home-based (SpO_2) settings. Performance metrics include ACC, $F1_{Macro}$, SEN, PPV, SPE across six external test cohorts.

Cohort	SpO_2					Gold				
	ACC	$F1_{Macro}$	SEN	PPV	SPE	ACC	$F1_{Macro}$	SEN	PPV	SPE
**SHHS2 (*n*=2,522)**	0.906	0.905	0.913	0.883	0.900	0.923	0.922	0.911	0.918	0.933
**MESA (*n*=1,846)**	0.891	0.889	0.844	0.966	0.958	0.884	0.882	0.831	0.967	0.959
**MROS (*n*=3,522)**	0.907	0.906	0.917	0.917	0.895	0.914	0.913	0.919	0.927	0.908
**CFS (*n*=576)**	0.927	0.918	0.934	0.851	0.924	0.939	0.929	0.869	0.935	0.972
**FDU-HSH (R) (*n*=327)**	0.933	0.933	0.901	0.960	0.964	-	-	-	-	-
**FDU-HSH (P) (*n*=265)**	0.891	0.891	0.812	0.983	0.983	-	-	-	-	-

Supplementary Table 9 | Binary classification performance of the AIX system with AHI cutoff at 30, comparing AHI<30 versus AHI≥30 subjects in both professional (Gold) and home-based (SpO_2) settings. Performance metrics include ACC, $F1_{Macro}$, SEN, PPV, SPE across six external test cohorts.

Cohort	SpO_2					Gold				
	ACC	$F1_{Macro}$	SEN	PPV	SPE	ACC	$F1_{Macro}$	SEN	PPV	SPE
**SHHS2 (*n*=2,522)**	0.955	0.926	0.910	0.851	0.965	0.960	0.933	0.891	0.889	0.975
**MESA (*n*=1,846)**	0.924	0.901	0.773	0.953	0.985	0.927	0.906	0.781	0.958	0.986
**MROS (*n*=3,522)**	0.943	0.923	0.867	0.899	0.968	0.946	0.928	0.898	0.885	0.962
**CFS (*n*=576)**	0.960	0.919	0.878	0.847	0.974	0.958	0.913	0.829	0.872	0.980
**FDU-HSH (R) (*n*=327)**	0.948	0.934	0.860	0.952	0.983	-	-	-	-	-
**FDU-HSH (P) (*n*=265)**	0.958	0.947	0.868	0.985	0.995	-	-	-	-	-

Supplementary Table 10 | Statistical relationship between interaction threshold intervals, FP rate, FN rate, and sample ratio for binary event classification at a 30-second granularity in the AIX system across FDU-HSH (R) and FDU-HSH (P) cohorts.

Cohort	Detail	Interactive threshold interval									
		**0.0-0.1**	**0.1-0.2**	**0.2-0.3**	**0.3-0.4**	**0.4-0.5**	**0.5-0.6**	**0.6-0.7**	**0.7-0.8**	**0.8-0.9**	**0.9-1.0**
**FDU-HSH (R) (*n*=327)**	**FP rate**	3.57%	0.96%	0.57%	0.45%	0.37%	0.37%	0.38%	0.42%	0.54%	2.91%
	**FN rate**	0.83%	0.22%	0.15%	0.13%	0.12%	0.12%	0.15%	0.12%	0.07%	0.25%
	**Sample ratio**	10.53%	3.66%	2.64%	2.37%	2.42%	2.88%	3.83%	4.22%	3.50%	25.12%
**FDU-HSH (P) (*n*=265)**	**FP rate**	3.33%	0.87%	0.53%	0.39%	0.33%	0.30%	0.30%	0.37%	0.49%	2.58%
	**FN rate**	1.21%	0.36%	0.24%	0.20%	0.17%	0.22%	0.23%	0.20%	0.11%	0.41%
	**Sample ratio**	10.67%	3.77%	2.67%	2.40%	2.42%	2.99%	3.89%	4.35%	3.62%	25.03%

Supplementary Table 11 | Performance gains from manual review by setting different interaction thresholds in the AIX system are analyzed, and the relationships between manual review ratios (%) and ACC, SEN, and SPE for binary event classification at a 30-second granularity are reported.

**Cohort**	**Detail**	**Interactive threshold interval**																		
		**0.00**	**0.05**	**0.10**	**0.15**	**0.20**	**0.25**	**0.30**	**0.35**	**0.40**	**0.45**	**0.50**	**0.55**	**0.60**	**0.65**	**0.70**	**0.75**	**0.80**	**0.85**	**0.90**
**FDU-HSH (R) (*n*=327)**	**ACC**	0.867	0.900	0.911	0.918	0.923	0.927	0.930	0.934	0.936	0.939	0.941	0.944	0.946	0.949	0.951	0.954	0.957	0.959	0.963
	**SEN**	0.914	0.938	0.945	0.950	0.954	0.957	0.959	0.962	0.964	0.966	0.969	0.971	0.973	0.976	0.979	0.981	0.983	0.984	0.986
	**SPE**	0.851	0.887	0.899	0.907	0.912	0.917	0.920	0.923	0.926	0.929	0.931	0.934	0.936	0.939	0.941	0.944	0.947	0.950	0.954
	**Ratio**	0.00	7.43	10.53	12.57	14.18	15.58	16.83	18.04	19.19	20.40	21.62	22.98	24.50	26.31	28.33	30.51	32.55	34.38	36.05
**FDU-HSH (P) (*n*=265)**	**ACC**	0.866	0.900	0.911	0.919	0.924	0.928	0.931	0.935	0.937	0.940	0.942	0.945	0.948	0.950	0.953	0.956	0.958	0.961	0.965
	**SEN**	0.876	0.907	0.918	0.925	0.930	0.935	0.939	0.943	0.946	0.949	0.952	0.956	0.960	0.964	0.968	0.972	0.975	0.977	0.979
	**SPE**	0.862	0.897	0.909	0.916	0.921	0.925	0.928	0.931	0.934	0.936	0.938	0.940	0.943	0.945	0.947	0.949	0.952	0.955	0.959
	**Ratio**	0.00	7.52	10.67	12.80	14.45	15.85	17.12	18.38	19.52	20.66	21.94	23.35	24.92	26.75	28.81	31.01	33.16	35.02	36.78

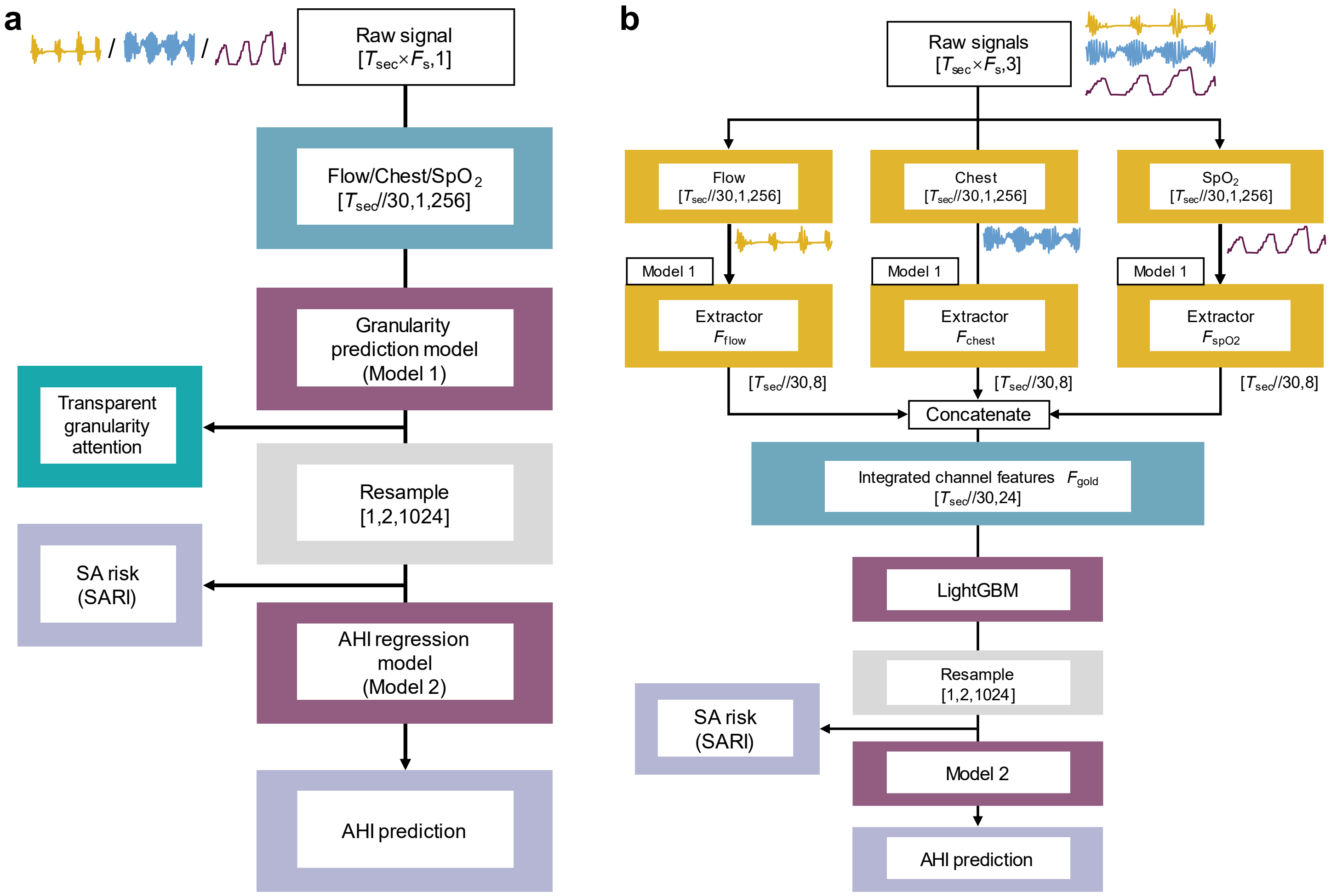

Supplementary Fig. 11 | AIX system operates within home (single-channel) and professional (multi-channel) workflows. (a) Single-channel signals for the entire night are segmented into 30-second granularity and fed into the granularity prediction model (Model 1). This model provides detailed 30-second AI analysis decision logic based on a transparent scale diffusion mechanism. Subsequently, the granular probabilities of normal and respiratory events for the entire night are resampled to a length of 1024 to report overnight SARI and train and predict with the AHI regression model (Model 2), resulting in the final AHI value. Two interpretability markers, 30-second transparent granularity attention and SARI, are obtained throughout the workflow. (b) Multi-channel signals for the entire night are split according to signal type, and a Model 1 is trained for each signal type. The trained Model 1 is used as a feature extractor to output 8 features per signal type for 30-second granularity. These features are concatenated to form 24 features. These features are trained using lightGBM to output multi-channel granular prediction probabilities. Finally, the resampled granular probabilities for the entire night are used for AHI regression with Model 2. Similar to the AIX single-channel workflow, the multi-channel workflow outputs the SARI and the 30-second transparent granularity attention for each signal type.

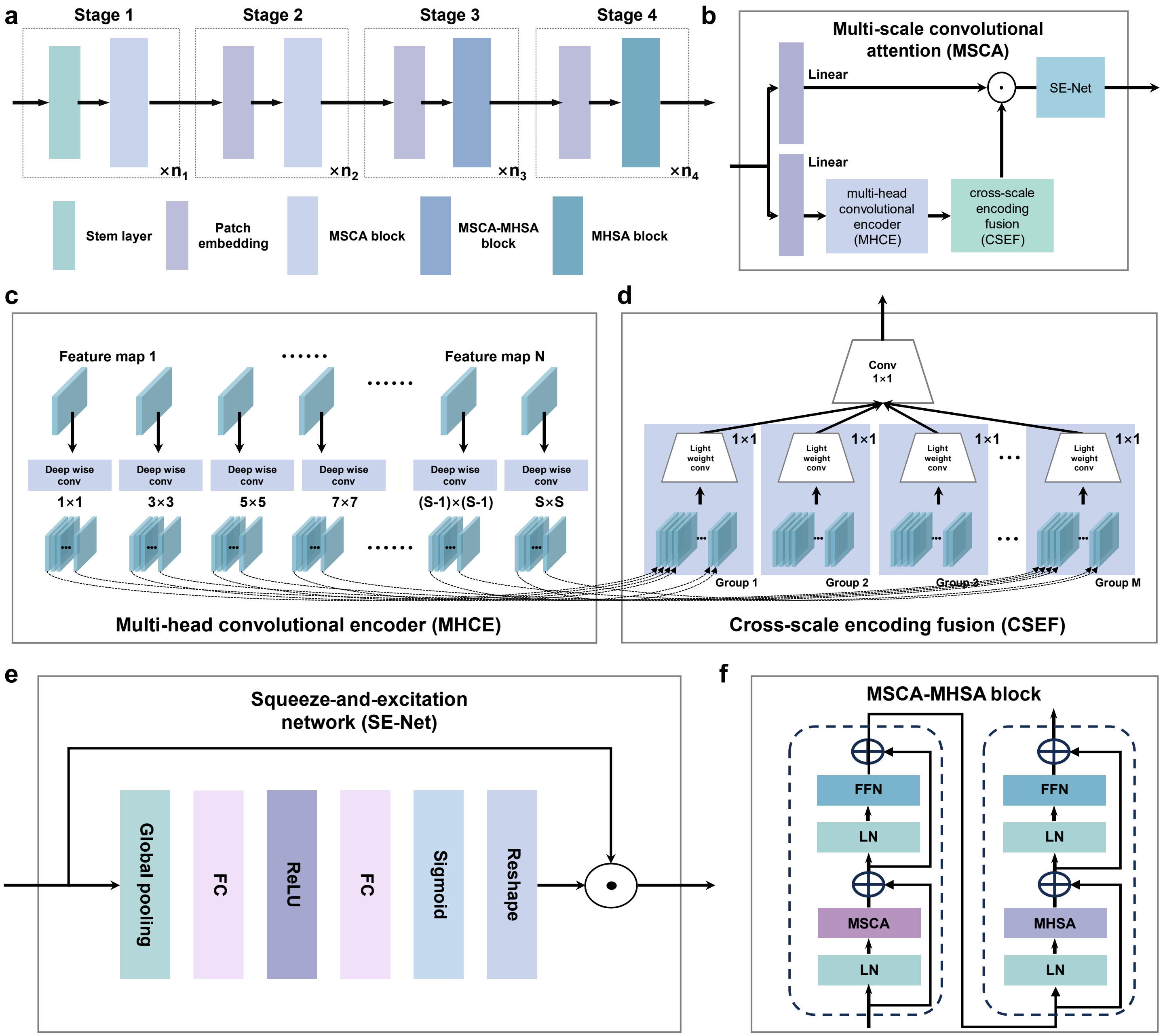

Supplementary Fig. 12 | Overall structure of the granularity prediction model with individual constituent blocks. (a) General structure of the granularity prediction model (TSD-Net). (b) Composition of MSCA block. (c) MHCE module. (d) CSEF module. (e) SE-Net. (f) Specific composition of MSCA-MHSA block.

Supplementary Table 12 | Details of the parameterization of TSD-Net. TSD-Net-T and TSD-Net-B represent the Tiny and Base versions of TSD-Net, respectively; "dim 64": An embedding dimension of 64; "sam.head 4": The MHCE module with 4 heads; "sam.ep.r 2": An expansion rate of 2 for the CSEF module; "msa.head 8": The MHSA module with 8 heads.

Layer name	Output size [B,C,L]	TSD-Net-T	TSD-Net-B
**Stem**	[B,64,64]	conv1d 2, 64-d, BN	conv1d 2, 64-d, BN
**Stage 1**	[B,64,64]	conv1d 2, 64-d, LN	conv1d 2, 64-d, LN
		[dim 64<br>sam.head 4<br>sam.ep.r 2] *1	[dim 64<br>sam.head 4<br>sam.ep.r 2] *2
**Stage 2**	[B,128,32]	conv1d 3, 128-d, LN	conv1d 3, 128-d, LN
		[dim 128<br>sam.head 4<br>sam.ep.r. 2] *1	[dim 128<br>sam.head 4<br>sam.ep.r. 2] *2
**Stage 3**	[B,256,16]	conv1d 3, 256-d, LN	conv1d 3, 256-d, LN
		[dim 256<br>sam.head 4<br>sam.ep.r 2<br>msa.head 8] *1	[dim 256<br>sam.head 4<br>sam.ep.r 2<br>msa.head 8] *8
**Stage 4**	[B,512,8]	conv1d 3, 512-d, LN	conv1d 3, 512-d, LN
		[dim 512<br>msa.head 16] *1	[dim 512<br>msa.head 16] *1
AdaptiveAvgPool1d, Flatten, 1-d, Sigmoid			

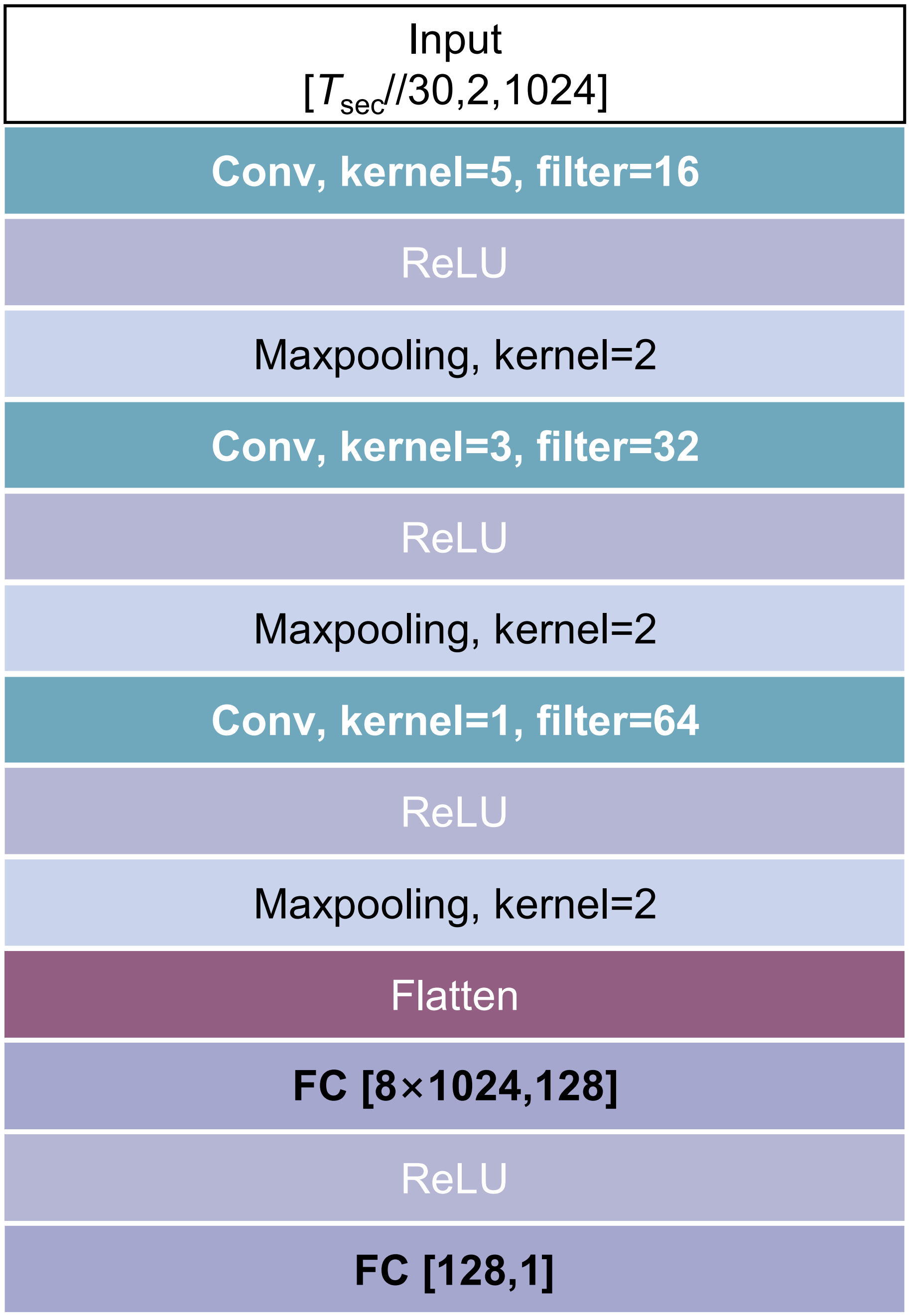

Supplementary Fig. 13 | Overall structure of the AHI regression model. The model consists of three 1D convolutional layers with kernel sizes of 5, 3, and 1 and filter quantities of 16, 32, and 64, respectively, followed by ReLU activations and MaxPooling layers. After flattening, two fully connected (FC) layers with ReLU activation complete the architecture. This network processes overnight granularity probability predictions to generate AHI estimates.

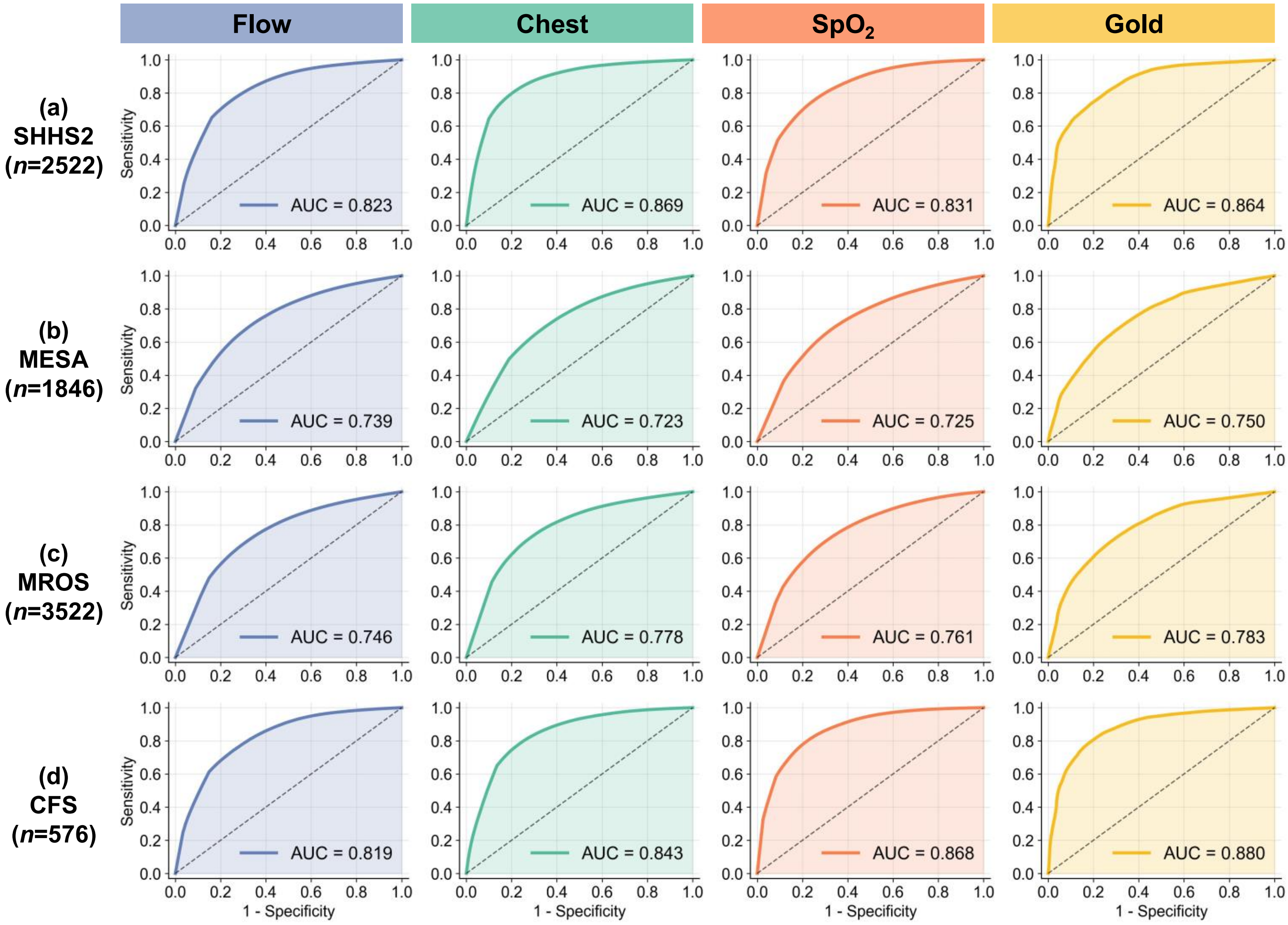

Supplementary Fig. 14 | Performance of the granularity prediction model in binary event detection (normal events versus respiratory events) based on different channel types across four external test cohorts (a. SHHS2, b. MESA, c. MROS, d. CFS) is demonstrated. Macro ROC curves are provided along with their corresponding AUC values.

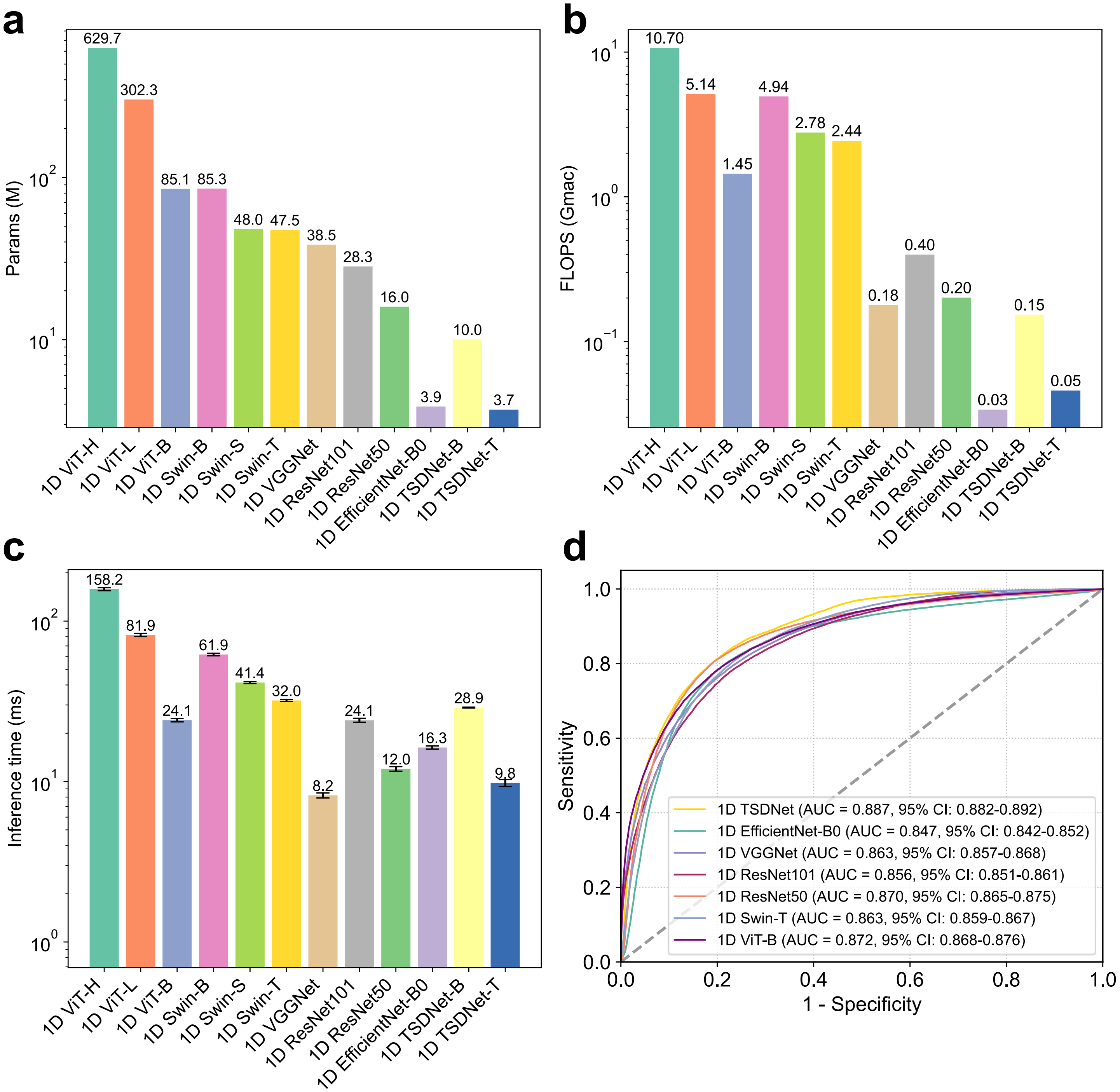

Supplementary Fig. 15 | Comparison of the proposed TSD-Net with other baseline models. (a) Comparison of model parameters. (b) Comparison of FLOPS. (c) Comparison of inference time for a single segment with shape [1,1,256]. (d) ROC curves showing the binary classification performance of different models. The models are trained and validated on SHHS1 cohort, and tested on four independent cohorts (SHHS2, MESA, MROS, and CFS) with 100,000 randomly sampled segments from each cohort (normal events *n*=311,557, respiratory events *n*=88,443). The test set is divided into 11 folds to demonstrate the error ranges. The plot shows the median ROC curves with their corresponding AUC values and 95% CIs.

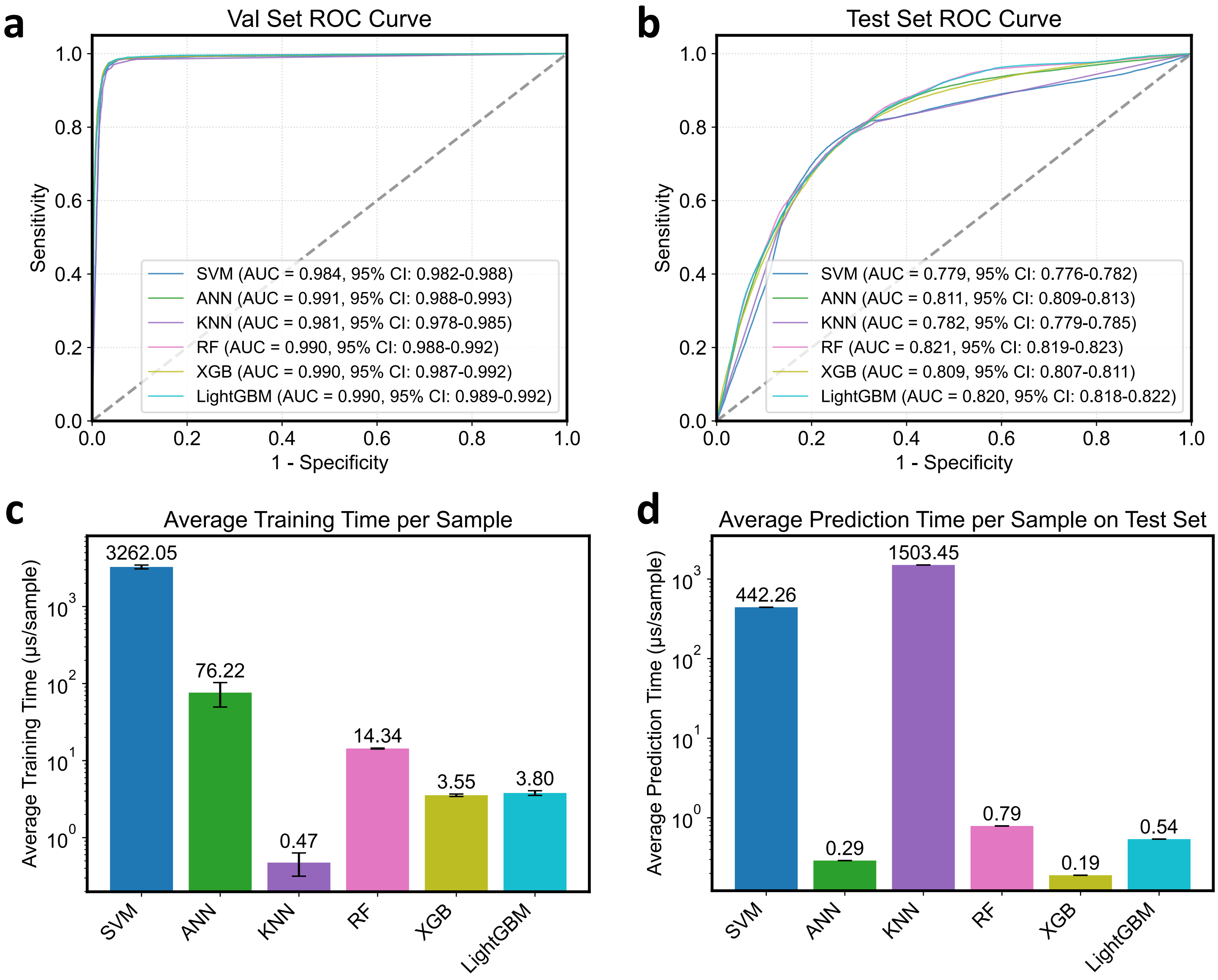

Supplementary Fig. 16 | Performance of various machine learning models for binary respiratory event classification in the Gold configuration is demonstrated. The study employs 11-fold cross-validation, utilizing the SHHS1 cohort as the training set. The test set comprises 100,000 samples (normal events *n*=78,446 and respiratory events *n*=21,554) randomly selected from four cohorts: SHHS2, MESA, MROS, and CFS (25,000 samples from each). The test set contains 78,446 normal events and 21,554 respiratory events. (a), (b) ROC curves for the validation and test sets, demonstrating the ROC curves and their corresponding AUC values for each model (median curve and 95% CI). (c), (d) Average training and inference times per sample (in microseconds) presented on a logarithmic scale.

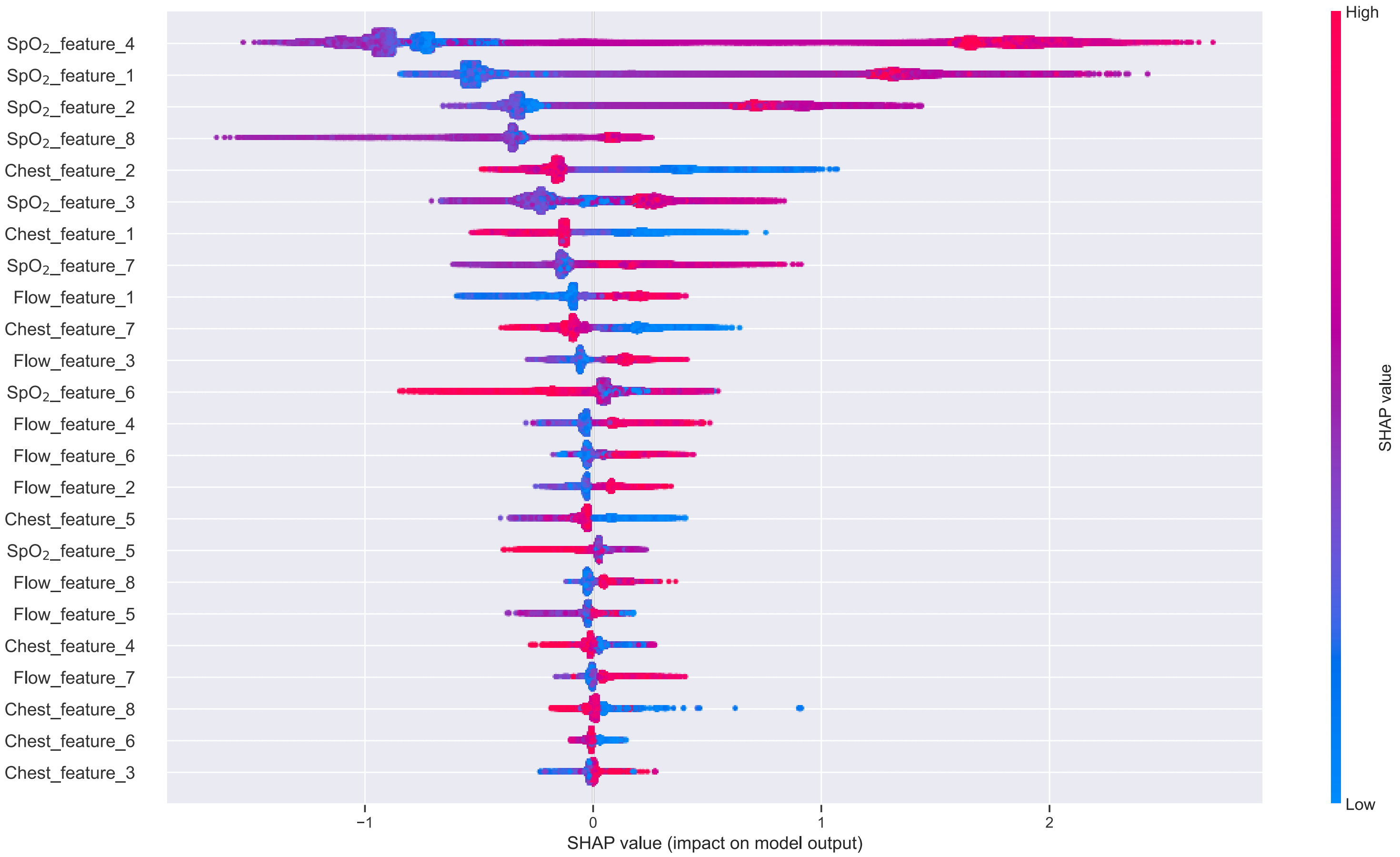

Supplementary Fig. 17 | Feature-level SHAP analysis of respiratory channels in model prediction. SHAP values showing the impact of individual features on model predictions, evaluated on 400,000 randomly sampled segments from four independent cohorts (SHHS2, MESA, MROS, and CFS with 100,000 segments each, normal events *n*=311,557, respiratory events *n*=88,443). The color represents the feature value (blue: low, red: high), and the horizontal position shows the SHAP value impact on model output. Features are ordered by their absolute SHAP values.

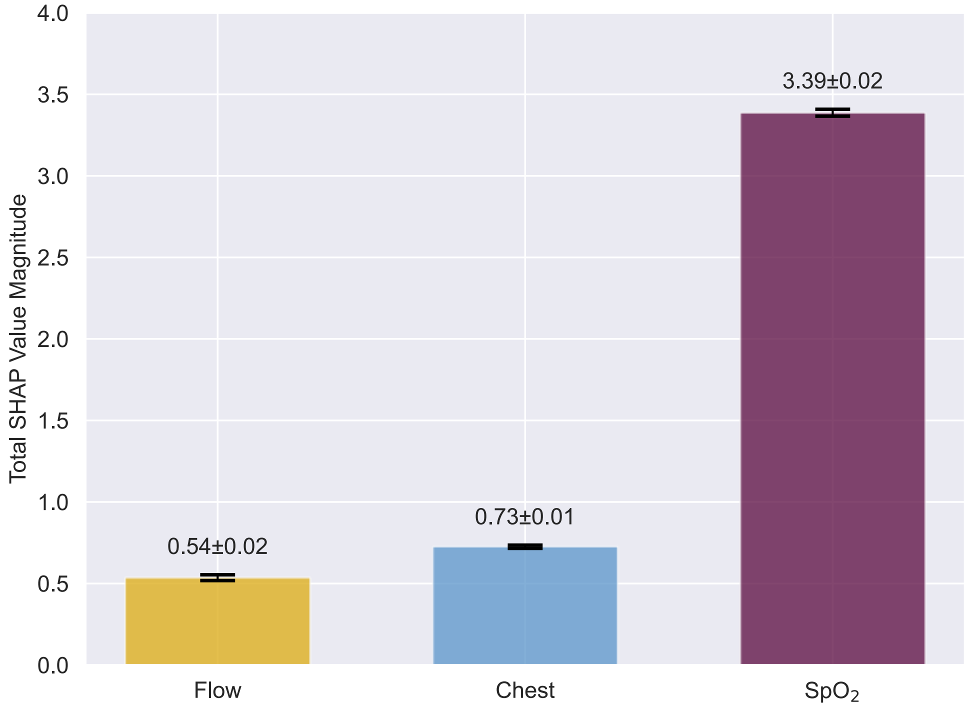

Supplementary Fig. 18 | Quantitative comparison of SHAP value magnitudes across respiratory channels. Comparison of total SHAP value magnitudes across different channels (Flow, Chest, and SpO_2). Values are presented as mean ± SD, demonstrating the relative importance of each channel type in the model's decision-making process.

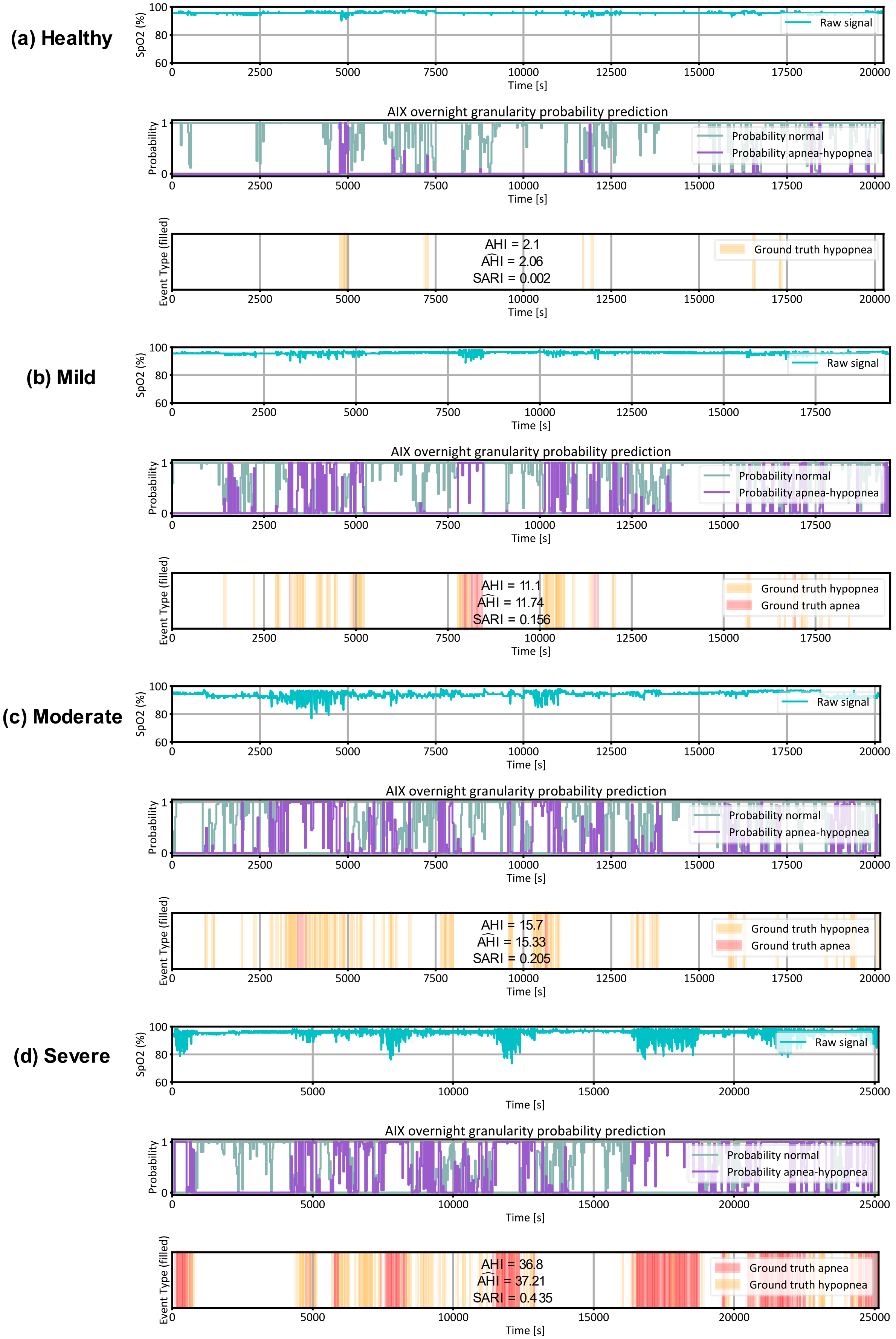

Supplementary Fig. 19 | Four different examples of SA severity levels from the real-world FDU-HSH test data are presented, based on single-channel SpO_2 information, showcasing overnight probability predictions along with manual and predicted AHI. (a) Healthy. (b) Mild. (c) Moderate (d) Severe.

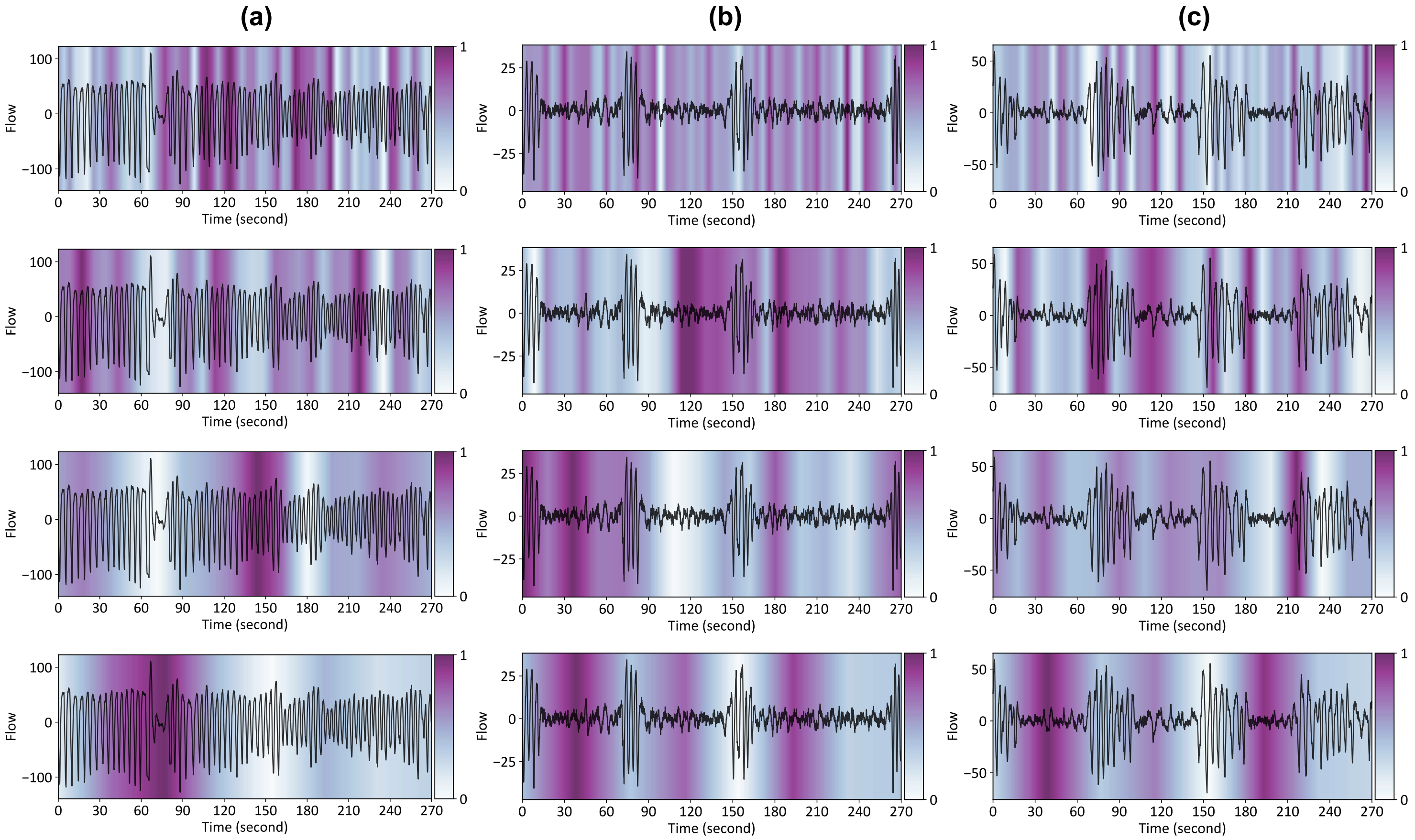

Supplementary Fig. 20 | Granular attention patterns in the AIX system enabled by transparent scale diffusion mechanisms. (a)-(c) showcase three typical examples with various transparent scales, where the model focuses on hypopnea/apnea patterns in Flow signals across four scales.

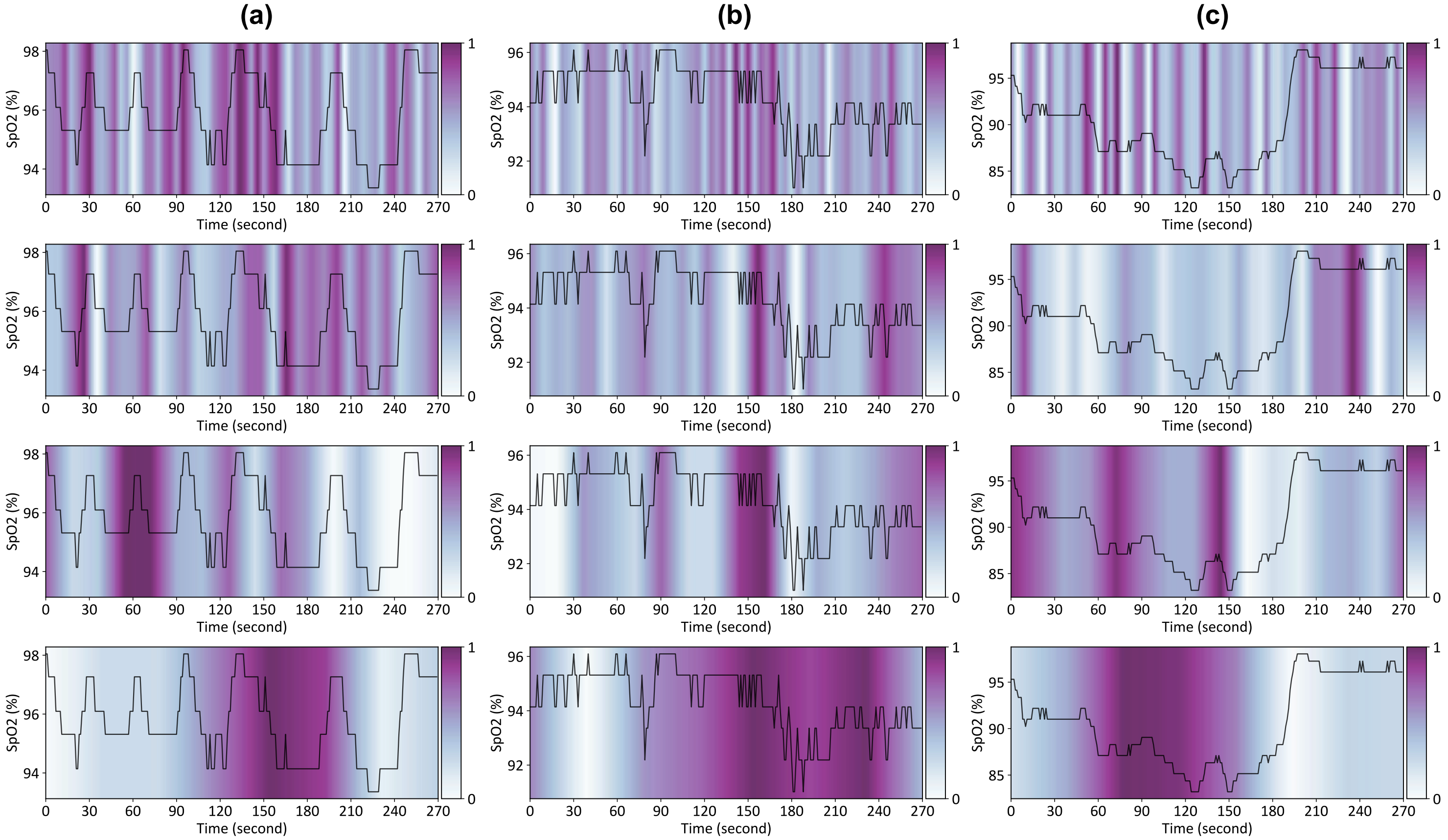

Supplementary Fig. 21 | Granular attention patterns in the AIX system enabled by transparent scale diffusion mechanisms. (a)-(c) showcase three typical examples with various transparent scales, where the model focuses on desaturation patterns in SpO_2 signals across four different scales.

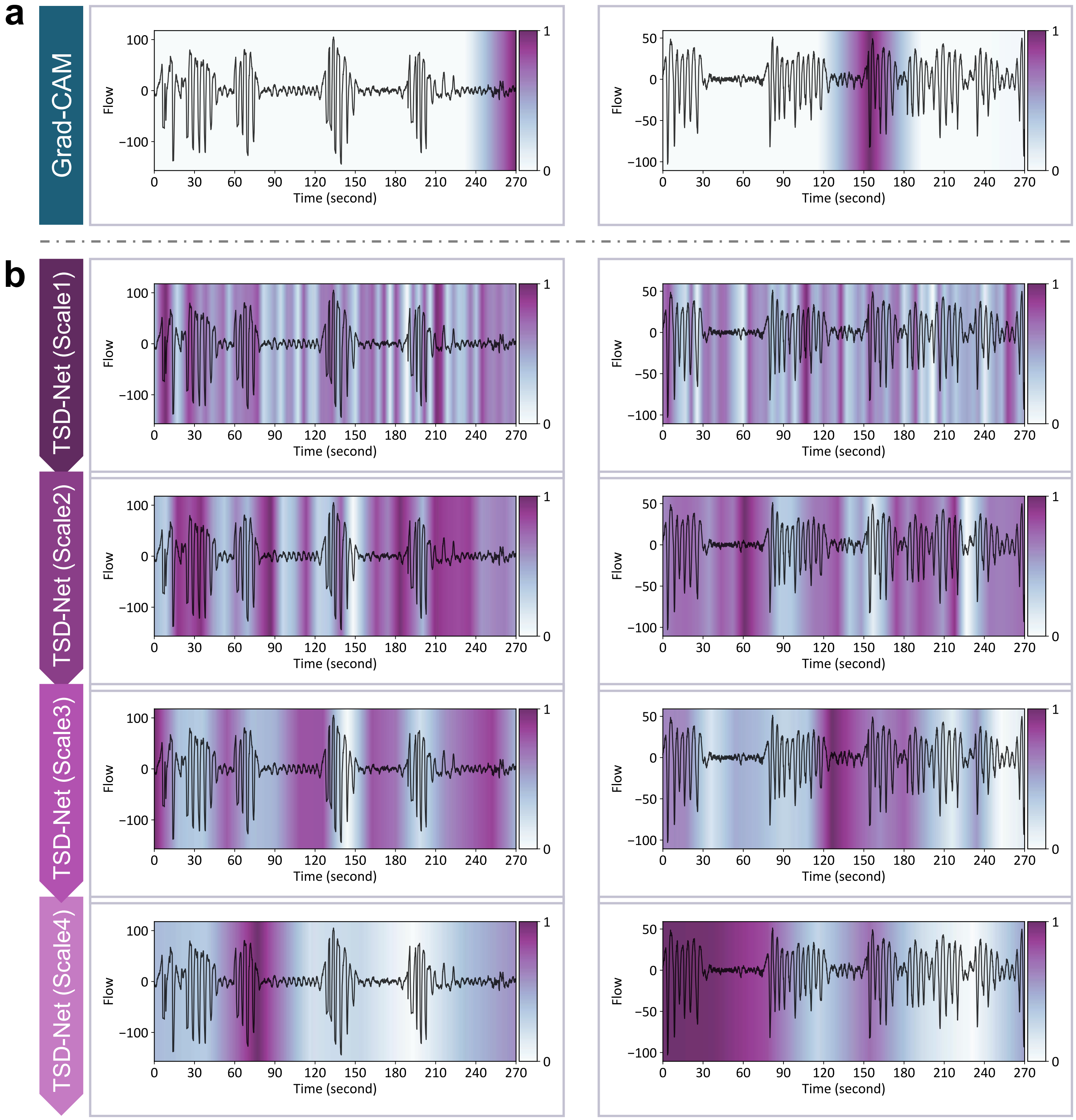

Supplementary Fig. 22 | Transparent visualization comparison of external test examples (Flow signals). (a) Grad-CAM results based on the last convolutional layer of ResNet50. (b) TSD-Net visualizations at four different scales (Scale 1-4).

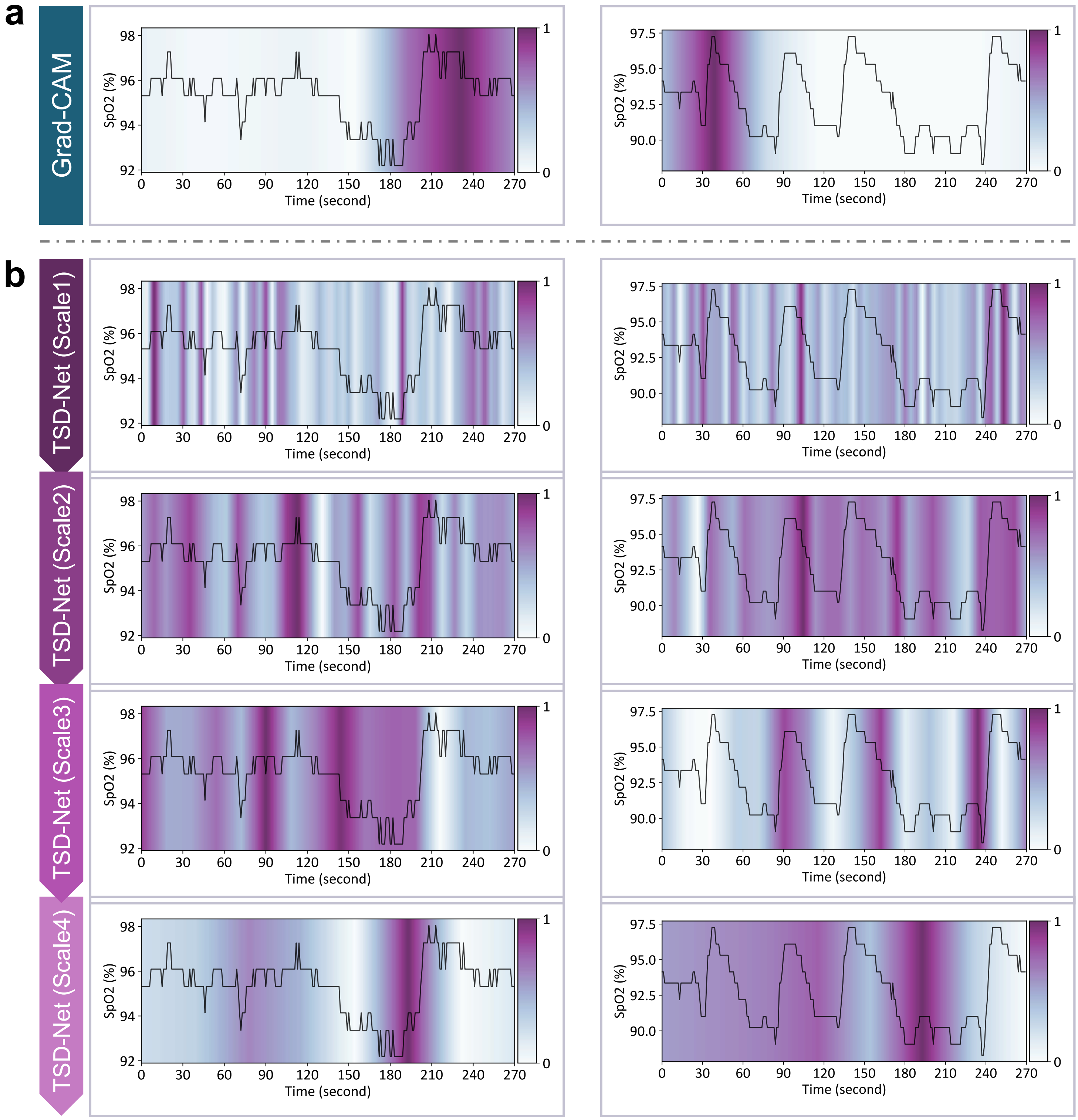

Supplementary Fig. 23 | Transparent visualization comparison of external test examples (SpO_2 signals). (a) Grad-CAM results based on the last convolutional layer of ResNet50. (b) TSD-Net visualizations at four different scales (Scale 1-4). The transparency scale diffusion mechanism of TSD-Net is shown to exhibit a hierarchical attention distribution from local details to global features, offering a finer feature representation capability compared to the Grad-CAM method, providing a multi-level interpretability perspective for the model's decision-making process.

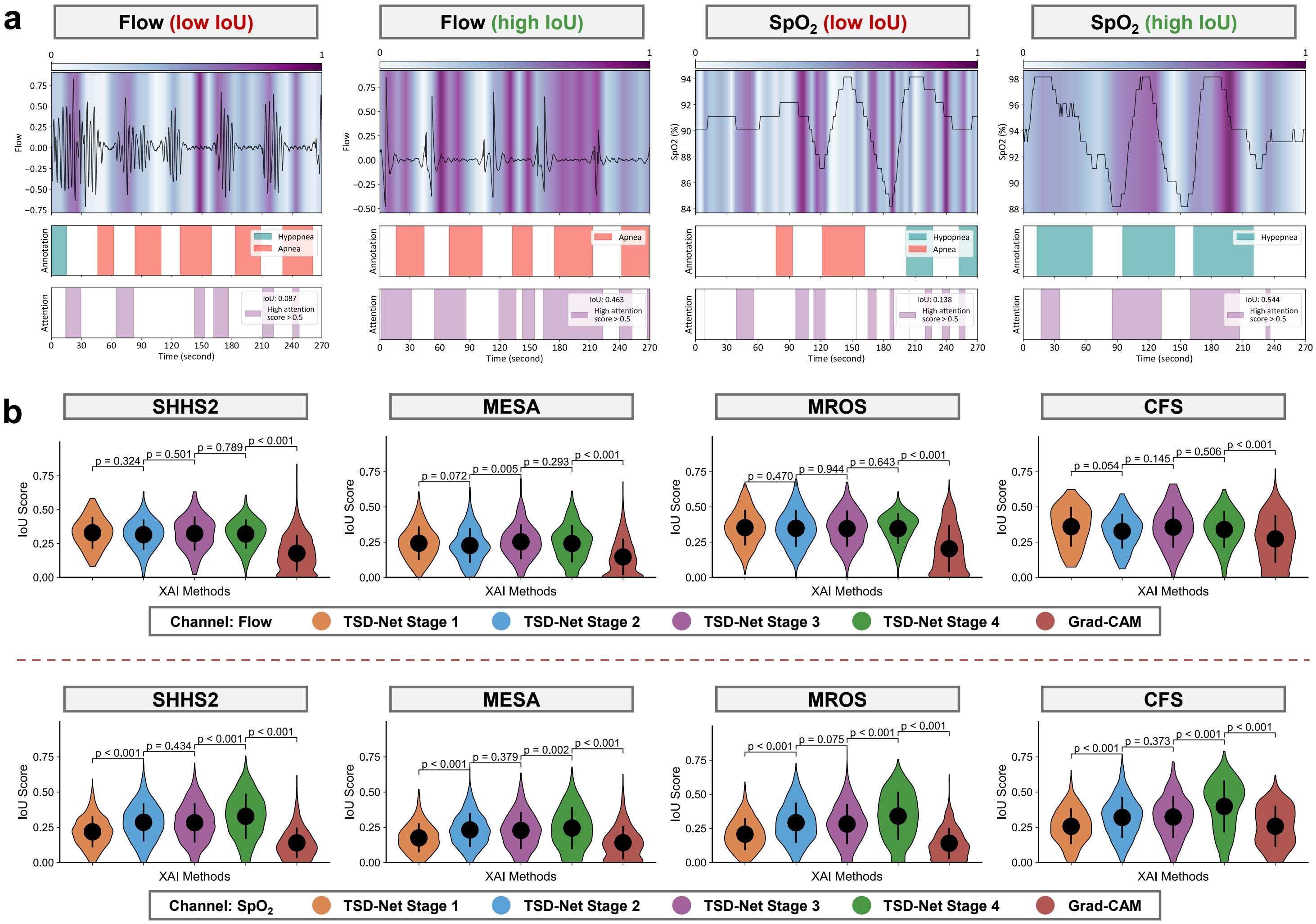

Supplementary Fig. 24 | Evaluation of XAI methods' interpretability and alignment with clinical event detection. (a) Representative examples demonstrating the alignment between attention heatmaps and expert-annotated respiratory events for Flow and SpO_2 signals. The original signals (top) are overlaid with attention heatmaps, where darker colors indicate higher attention weights. Expert annotations (middle) show the temporal locations of respiratory events (red for apnea, cyan for hypopnea). The IoU scores (bottom) quantify the overlap between model attention and expert annotations, with cases showing both low and high IoU scenarios. (b) Quantitative comparison of IoU scores across four sleep cohorts (SHHS2, MESA, MROS, and CFS). The violin plots show IoU score distributions for four TSD-Net stages and Grad-CAM on Flow (top) and SpO_2 (bottom) channel. Black dots and lines represent mean ± SD. *p*-values from two-tailed Mann-Whitney U tests indicate statistical significance between adjacent methods.

Supplementary Table 13 | Quantitative comparison of XAI methods using IoU scores and statistical significance across four sleep cohorts (SHHS2, MESA, MROS, and CFS). IoU values are presented as mean ± SD, and median. *p*-values are calculated using two-tailed Mann-Whitney U tests between pairwise TSD-Net stages and between TSD-Net stages and Grad-CAM.

**Cohort**	**Flow**	**TSD-Net**				**Grad-CAM**
		Stage 1	Stage 2	Stage 3	Stage 4	
**SHHS2**	IoU (Mean±Std; Median)	0.330±0.111; 0.333	0.315±0.106; 0.320	0.324±0.121; 0.330	0.321±0.101; 0.335	0.178±0.129; 0.168
	p-value	Stage 1 versus Stage 2: 3.236×10^{-1}; Stage 1 versus Stage 3: 7.611×10^{-1}<br>Stage 1 versus Stage 4: 5.796×10^{-1}; Stage 2 versus Stage 3: 5.011×10^{-1}<br>Stage 2 versus Stage 4: 6.083×10^{-1}; Stage 3 versus Stage 4: 7.887×10^{-1}				Grad-CAM versus Stage 1: 4.450×10^{-42}<br>Grad-CAM versus Stage 2: 4.264×10^{-38}<br>Grad-CAM versus Stage 3: 2.761×10^{-38}<br>Grad-CAM versus Stage 4: 5.192×10^{-41}
**MESA**	IoU (Mean±Std; Median)	0.243±0.114; 0.238	0.227±0.118; 0.217	0.253±0.118; 0.249	0.240±0.128; 0.245	0.145±0.124; 0.127
	p-value	Stage 1 versus Stage 2: 7.232×10^{-2}; Stage 1 versus Stage 3: 2.723×10^{-1}<br>Stage 1 versus Stage 4: 9.898×10^{-1}; Stage 2 versus Stage 3: 5.142×10^{-3}<br>Stage 2 versus Stage 4: 1.140×10^{-1}; Stage 3 versus Stage 4: 2.926×10^{-1}				Grad-CAM versus Stage 1: 3.840×10^{-43}<br>Grad-CAM versus Stage 2: 1.112×10^{-31}<br>Grad-CAM versus Stage 3: 1.926×10^{-49}<br>Grad-CAM versus Stage 4: 6.792×10^{-37}
**MROS**	IoU (Mean±Std; Median)	0.353±0.121; 0.350	0.348±0.125; 0.343	0.346±0.122; 0.354	0.346±0.103; 0.365	0.204±0.160; 0.181
	p-value	Stage 1 versus Stage 2: 4.702×10^{-1}; Stage 1 versus Stage 3: 5.322×10^{-1}<br>Stage 1 versus Stage 4: 9.803×10^{-1}; Stage 2 versus Stage 3: 9.438×10^{-1}<br>Stage 2 versus Stage 4: 5.061×10^{-1}; Stage 3 versus Stage 4: 6.433×10^{-1}				Grad-CAM versus Stage 1: 2.043×10^{-80}<br>Grad-CAM versus Stage 2: 2.663×10^{-75}<br>Grad-CAM versus Stage 3: 8.895×10^{-75}<br>Grad-CAM versus Stage 4: 1.180×10^{-79}
**CFS**	IoU (Mean±Std; Median)	0.360±0.136; 0.370	0.328±0.117; 0.336	0.356±0.140; 0.357	0.340±0.125; 0.356	0.273±0.165; 0.278
	p-value	Stage 1 versus Stage 2: 5.433×10^{-2}; Stage 1 versus Stage 3: 7.000×10^{-1}<br>Stage 1 versus Stage 4: 2.523×10^{-1}; Stage 2 versus Stage 3: 1.454×10^{-1}<br>Stage 2 versus Stage 4: 4.039×10^{-1}; Stage 3 versus Stage 4: 5.063×10^{-1}				Grad-CAM versus Stage 1: 6.310×10^{-8}<br>Grad-CAM versus Stage 2: 4.006×10^{-4}<br>Grad-CAM versus Stage 3: 6.283×10^{-7}<br>Grad-CAM versus Stage 4: 1.599×10^{-5}

**Cohort**	**SpO_2**	**TSD-Net**				**Grad-CAM**
		Stage 1	Stage 2	Stage 3	Stage 4	
**SHHS2**	IoU (Mean±Std; Median)	0.218±0.105; 0.216	0.286±0.130; 0.285	0.283±0.135; 0.277	0.328±0.154; 0.333	0.140±0.103; 0.125
	p-value	Stage 1 versus Stage 2: 1.130×10^{-59}; Stage 1 versus Stage 3: 7.005×10^{-51}<br>Stage 1 versus Stage 4: 1.106×10^{-116}; Stage 2 versus Stage 3: 4.343×10^{-1}<br>Stage 2 versus Stage 4: 2.370×10^{-19}; Stage 3 versus Stage 4: 3.825×10^{-21}				Grad-CAM versus Stage 1: 1.640×10^{-100}<br>Grad-CAM versus Stage 2: 4.667×10^{-213}<br>Grad-CAM versus Stage 3: 3.725×10^{-199}<br>Grad-CAM versus Stage 4: 3.534×10^{-252}
**MESA**	IoU (Mean±Std; Median)	0.175±0.098; 0.169	0.231±0.114; 0.226	0.228±0.126; 0.222	0.244±0.143; 0.239	0.141±0.113; 0.129
	p-value	Stage 1 versus Stage 2: 2.882×10^{-40}; Stage 1 versus Stage 3: 5.848×10^{-31}<br>Stage 1 versus Stage 4: 6.391×10^{-42}; Stage 2 versus Stage 3: 3.793×10^{-1}<br>Stage 2 versus Stage 4: 1.691×10^{-2}; Stage 3 versus Stage 4: 2.425×10^{-3}				Grad-CAM versus Stage 1: 1.452×10^{-19}<br>Grad-CAM versus Stage 2: 8.649×10^{-80}<br>Grad-CAM versus Stage 3: 1.792×10^{-66}<br>Grad-CAM versus Stage 4: 1.265×10^{-74}
**MROS**	IoU (Mean±Std; Median)	0.207±0.112; 0.199	0.292±0.142; 0.284	0.282±0.142; 0.280	0.340±0.172; 0.344	0.141±0.107; 0.125
	p-value	Stage 1 versus Stage 2: 1.351×10^{-90}; Stage 1 versus Stage 3: 7.417×10^{-76}<br>Stage 1 versus Stage 4: 2.169×10^{-161}; Stage 2 versus Stage 3: 7.476×10^{-2}<br>Stage 2 versus Stage 4: 1.079×10^{-24}; Stage 3 versus Stage 4: 4.145×10^{-33}				Grad-CAM versus Stage 1: 4.038×10^{-86}<br>Grad-CAM versus Stage 2: 7.766×10^{-260}<br>Grad-CAM versus Stage 3: 4.595×10^{-234}<br>Grad-CAM versus Stage 4: 3.944×10^{-307}
**CFS**	IoU (Mean±Std; Median)	0.257±0.121; 0.256	0.320±0.139; 0.321	0.323±0.144; 0.328	0.398±0.179; 0.431	0.258±0.140; 0.258
	p-value	Stage 1 versus Stage 2: 9.361×10^{-39}; Stage 1 versus Stage 3: 2.300×10^{-42}<br>Stage 1 versus Stage 4: 3.414×10^{-129}; Stage 2 versus Stage 3: 3.729×10^{-1}<br>Stage 2 versus Stage 4: 7.396×10^{-49}; Stage 3 versus Stage 4: 3.338×10^{-44}				Grad-CAM versus Stage 1: 8.444×10^{-1}<br>Grad-CAM versus Stage 2: 9.880×10^{-32}<br>Grad-CAM versus Stage 3: 1.031×10^{-34}<br>Grad-CAM versus Stage 4: 2.330×10^{-122}

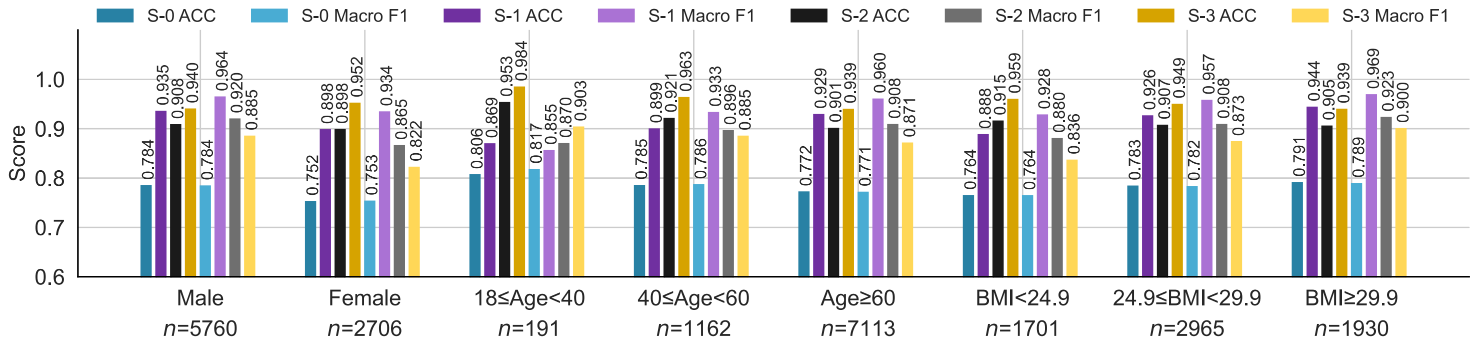

Supplementary Fig. 25 | Performance of AIX under single-channel SpO_2 configuration across different gender, age, and BMI groups. S-0, S-1, S-2, and S-3 represent four classification scenarios: S-0, S-1, S-2, and S-3 represent four classification scenarios: four-level SA classification, AHI<5 versus AHI≥5, AHI<15 versus AHI≥15, and AHI<30 versus AHI≥30.

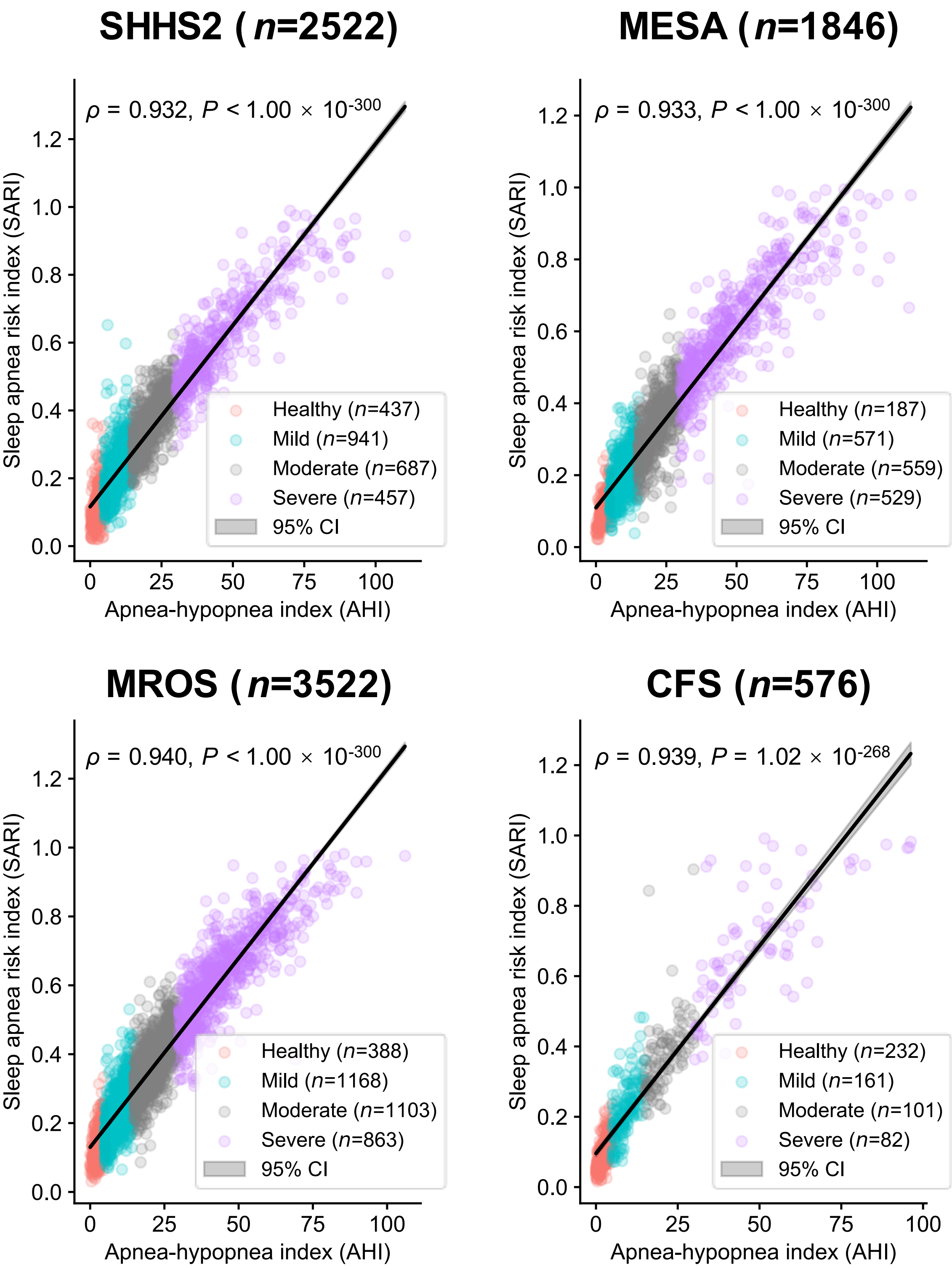

Supplementary Fig. 26 | Scatter plots of the overnight average risk coefficient and AHI based on single-channel SpO_2 signals are generated by the AIX system (SHHS2, MESA, MROS, and CFS). The Spearman correlation coefficient and *p*-value from a two-sided test are presented, with the diagonal line representing a linear regression model with a 95% CI. Four colors of scatter points are used to represent healthy individuals, mild, moderate, and severe SA patients, respectively.

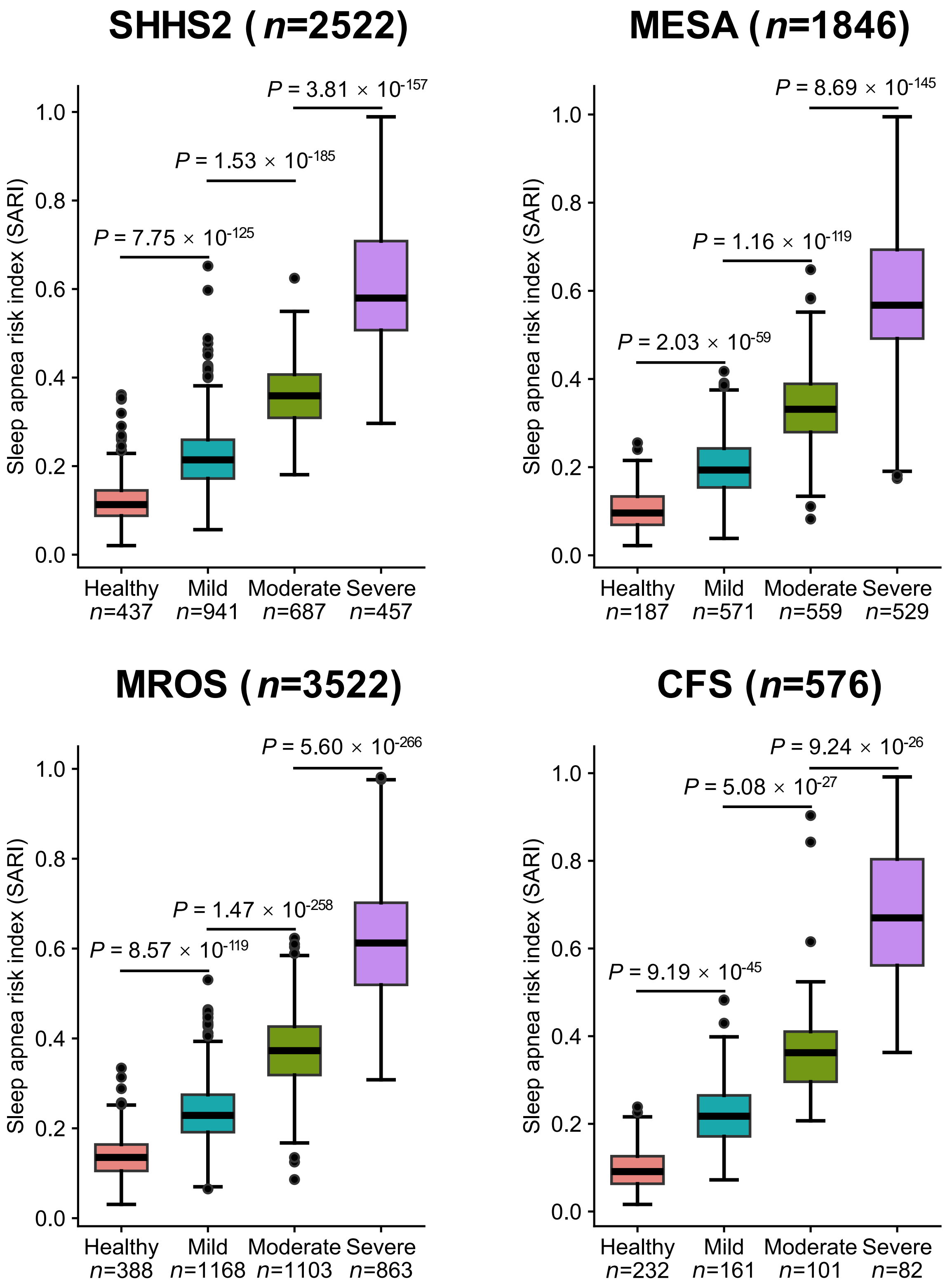

Supplementary Fig. 27 | Assessment of SA risk based on single-channel SpO_2 signals is conducted by the AIX system across four external test cohorts (SHHS2, MESA, MROS, and CFS). Performance is demonstrated in different SA severity groups, with *p*-values calculated using the two-tailed Mann-Whitney U test.

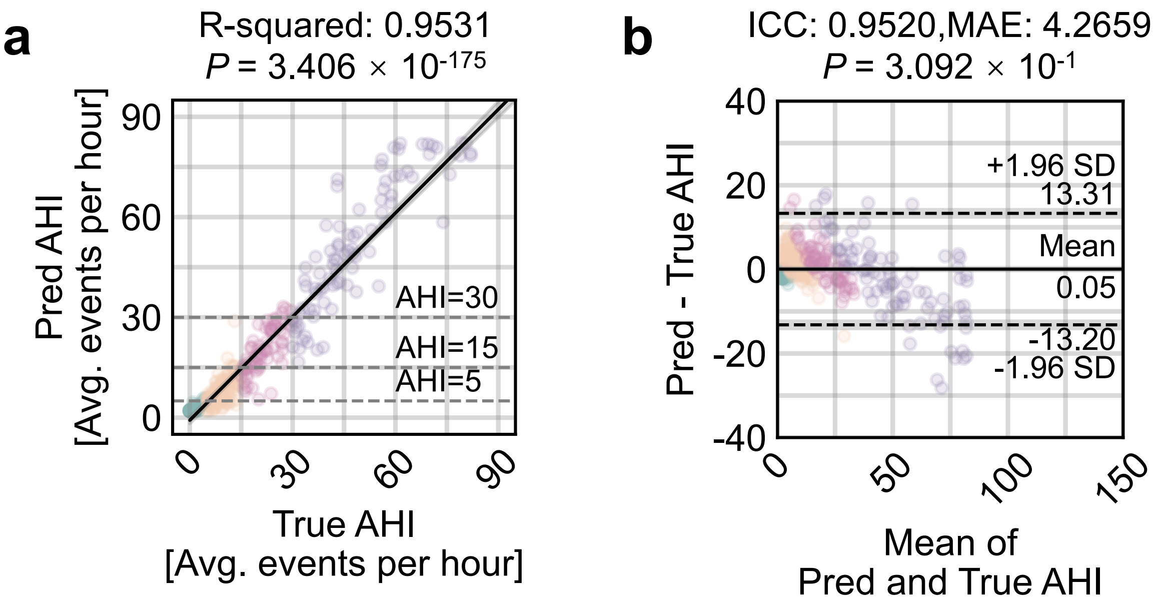

Supplementary Fig. 28 | AIX system presents the scatter plot of AHI regression correlation and the Bland-Altman plot based on single-channel SpO_2 signals in the FDU-HSH retrospective cohort (*n*=327 subjects). (a) The scatter plot illustrates the correlation between manual AHI and predicted AHI. The R^2 value is calculated, along with the diagonal representing the linear regression model and the two-sided *p*-value. (b) The Bland-Altman plot displays the comparison of manual and predicted AHI, with error lines positioned at ±1.96 SD. The *p*-value is computed using the two-sided Wilcoxon signed-rank test, and the ICC is provided, along with the MAE of predicted AHI for all subjects. Green, yellow, pink, and purple scatter points represent healthy individuals, mild SA, moderate SA, and severe SA patients, respectively.

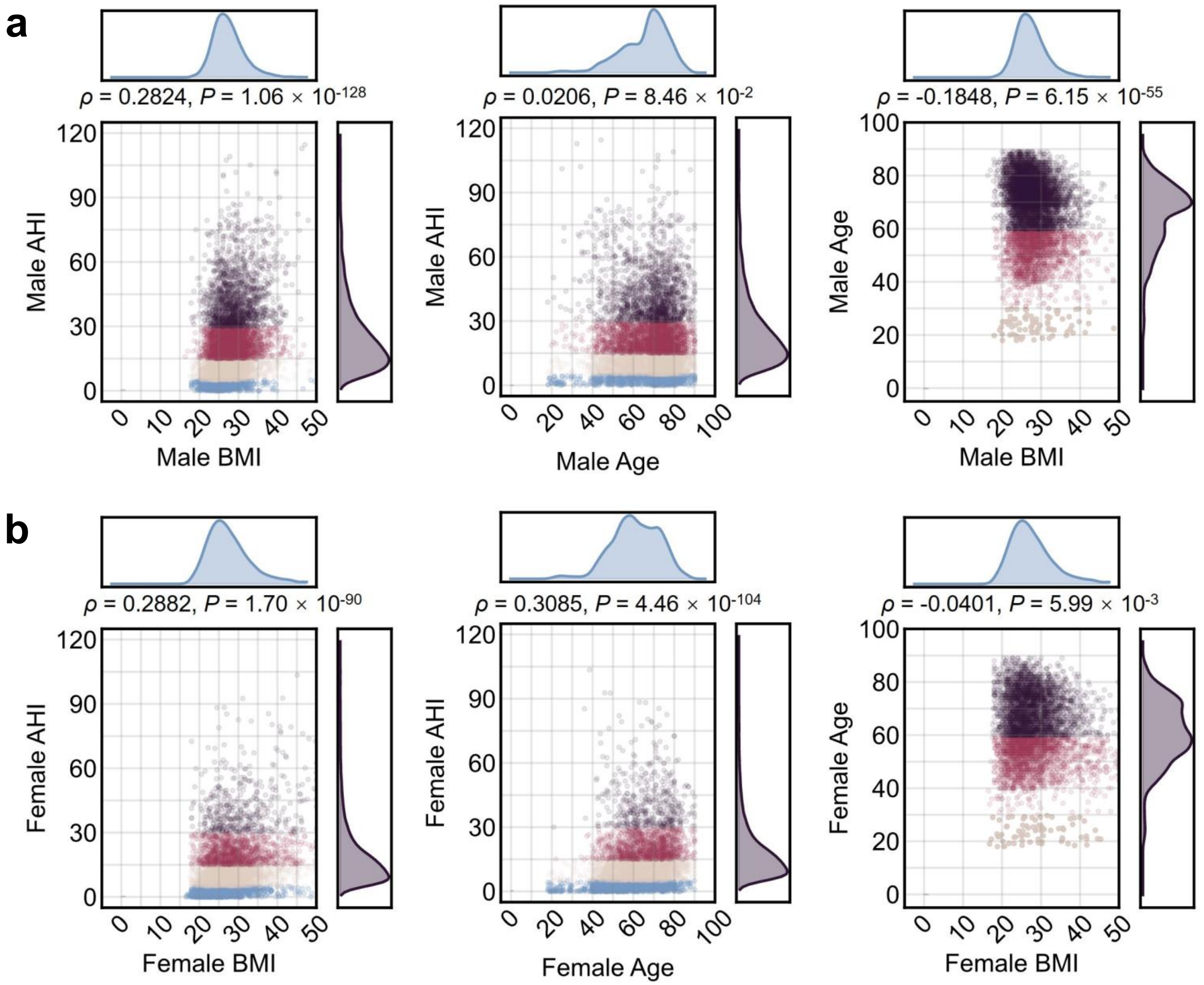

Supplementary Fig. 29 | Correlation analyses (males, *n*=7,013 subjects; females, *n*=4,694 subjects) among AHI, BMI, and age are conducted using all available measurement data from SHHS1, SHHS2, MROS, CFS, and FDU-HSH (retrospective/prospective). The Spearman's ρ and two-sided test p-values between pairs of features are presented for the male group (a) and the female group (b). The distribution of features is shown using KDE. In AHI groups, different colors (blue, light yellow, red, and black) represent healthy (AHI<5), mild (5≤AHI<15), moderate (15≤AHI<30), and severe (AHI≥30) subjects, respectively. In age groups, youth (18≤age<30, light yellow), middle-aged adults (30≤age<60, red), and older adults (age≥60, black) are distinguished by different color scatter points.

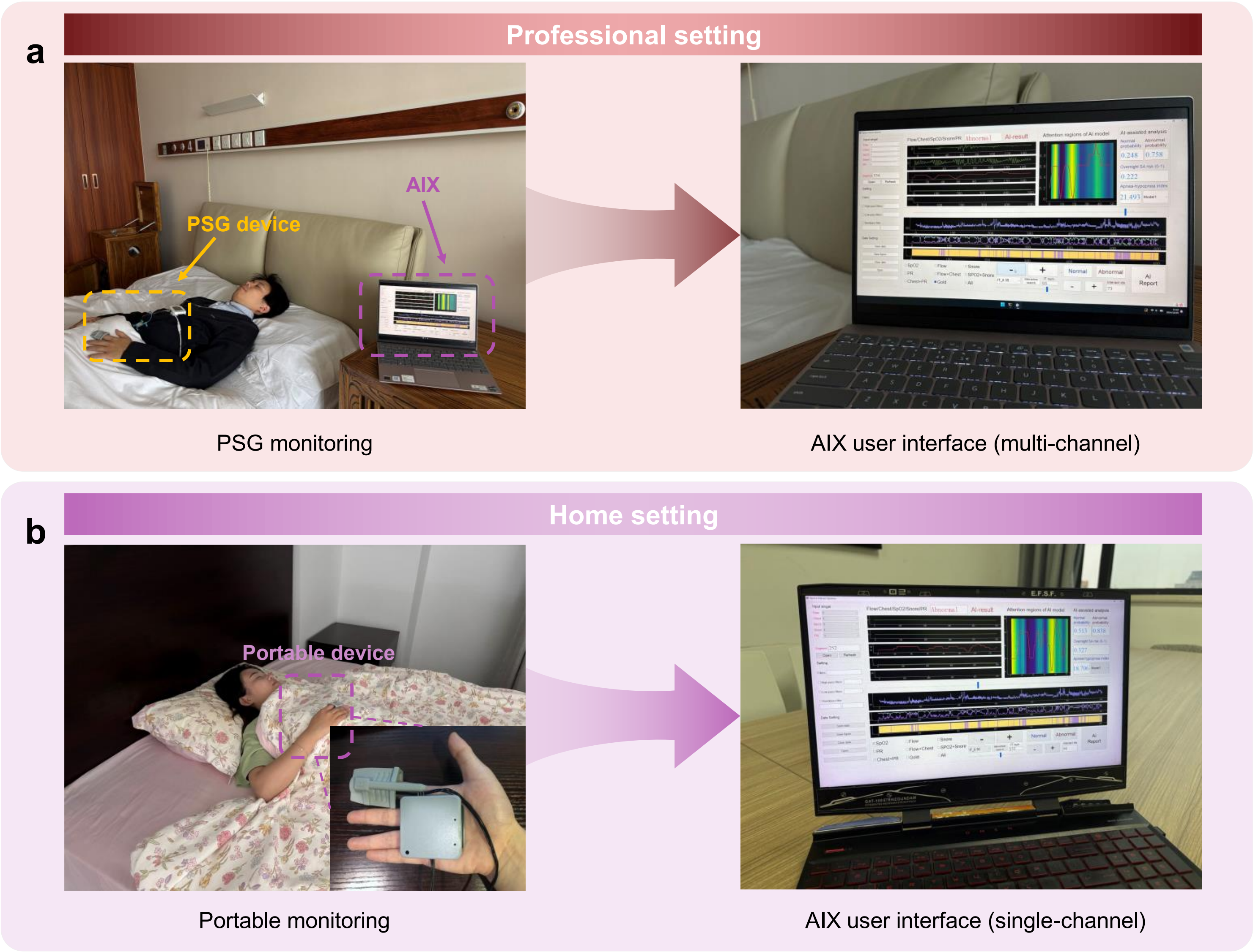

Supplementary Fig. 30 | Application deployments of the AIX system in both professional medical and home settings are presented. (a) In the professional setting, sleep monitoring is conducted through PSG, with the system supporting multi-channel signal analysis. (b) In the home setting, portable monitoring is achieved using wearable devices, and the system provides a single-channel analysis interface. Both scenarios demonstrate the workflow from data collection and AIX system deployment to the user interface, highlighting the adaptability and flexibility of AIX in different monitoring environments.

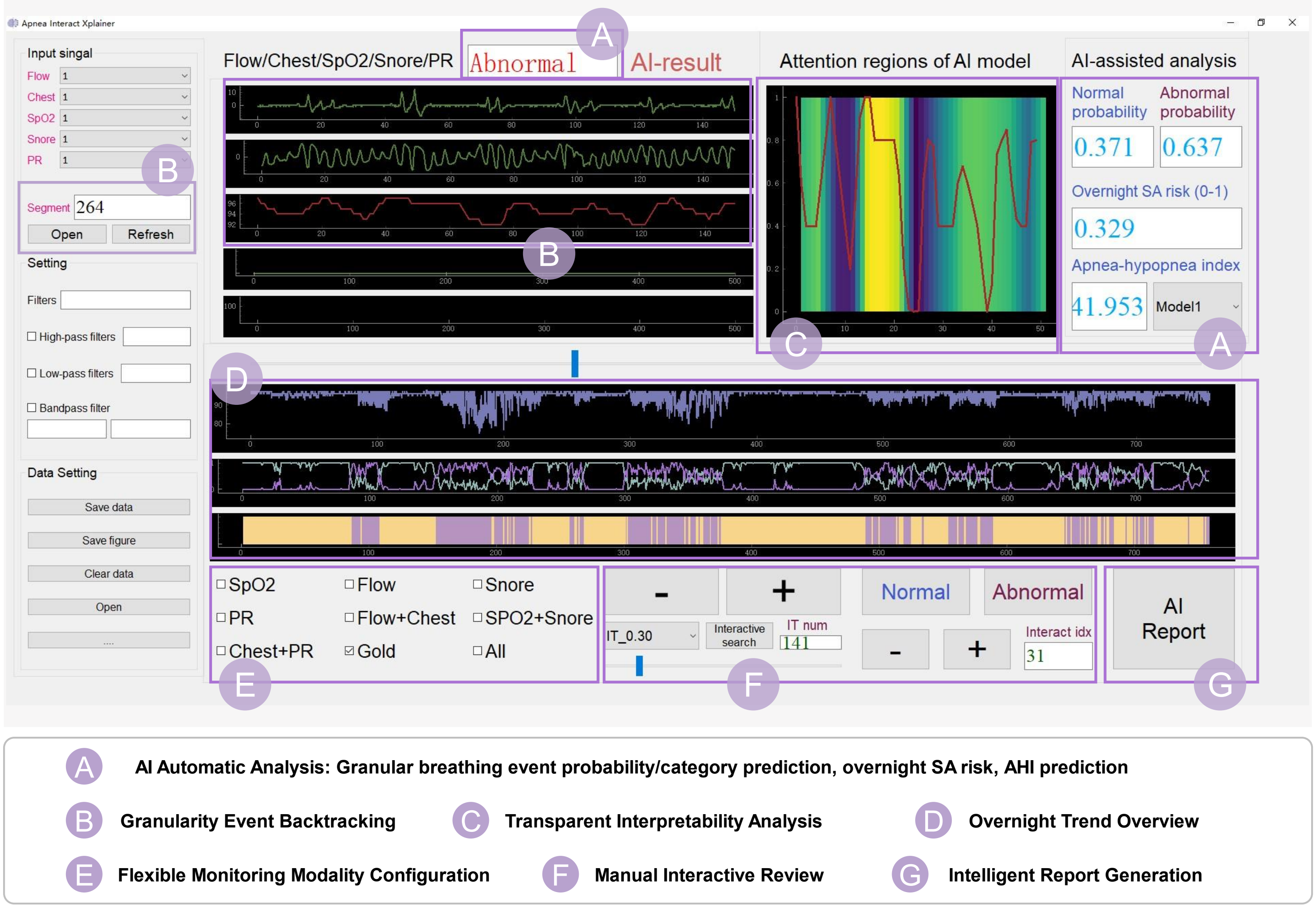

Supplementary Fig. 31 | Introduction to the AIX system operating interface and the functions of each component. (A) AI analysis results and classification display; (B) Signal input configuration and segment selection; (C) Attention visualization showing model's focus regions; (D) Overnight trend analysis including SpO_2 and respiratory event probability; (E) Multi-channel configuration options; (F) Interactive analysis controls; (G) Report generation function. This interface integrates signal visualization, AI analysis, and sleep report generation into a unified system for SA diagnosis.

Personal Information

Name:	____	Gender:	____
Age:	____	BMI:	____
Date:	____	Record ID:	____

Sleep Monitoring Results

Total Recording Time:	5.03 hours
Effective Analysis Time:	5.03 hours
Normal Sleep Time:	4.12 hours (82.1%)
Abnormal Breathing Time:	0.90 hours (17.9%)
Sleep Efficiency:	82.1%
Analysis Success Rate:	100%

Respiratory Events Analysis

Apnea Hypopnea Index (AHI):	21.5 events/hour
Longest Continuous Events:	18 segments (540 seconds)
Total Apnea Episodes:	108 events
Event Density:	21.5 events/hour
Normal Breathing Segments:	495 segments
Event Frequency:	0.6 events/100s
Average Event Duration:	30.0 seconds
Event Probability:	17.9%

Oxygen Saturation Analysis

Mean SpO2:	92.7% ± 1.3%
Maximum SpO2:	99.0%
Minimum SpO2:	89.0%
Time SpO2 < 90%:	0.0%
Time SpO2 < 85%:	0.0%
SpO2 Variability:	1.3%
Baseline SpO2:	95.0%

Diagnosis and Recommendations

Moderate Sleep Apnea Syndrome, predominantly obstructive events with moderate nocturnal hypoxemia.

Recommendations:
1. Adopt side sleeping position
2. Maintain balanced diet and weight control
3. Nasal CPAP therapy is recommended

Monitoring Charts

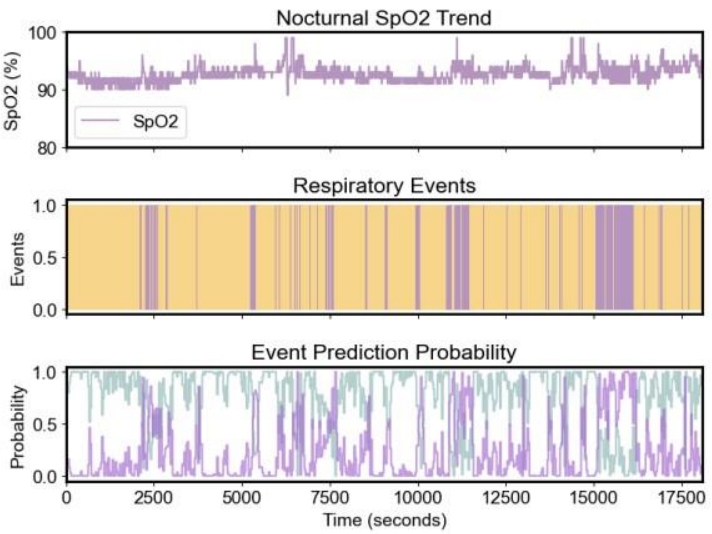

Sleep Apnea Risk Assessment

Sleep Apnea Risk Score:	0.22
Risk Level:	Low Risk (Low: <0.25, Moderate: 0.25-0.50, High: 0.50-0.75, Very High: ≥0.75)
AHI-based Severity:	Moderate (Normal: <5, Mild: 5-15, Moderate: 15-30, Severe: >30)
SpO2-based Risk:	Moderate Risk (based on minimum SpO2 and time below 90%)
Note:	Risk levels are calculated based on multiple physiological parameters

Supplementary Fig. 32 | AI-generated comprehensive SA analysis report of AIX. The report includes personal information, sleep monitoring metrics, respiratory events analysis, and oxygen saturation data. The monitoring charts display (A) nocturnal SpO_2 trend, (B) respiratory events visualization with color-coded normal (yellow) and apnea/hypopnea (purple) periods, and (C) continuous event prediction probabilities. The report also provides detailed risk assessment based on multiple physiological parameters, with diagnostic recommendations following clinical guidelines. This automated analysis demonstrates the capability of transparent AI in providing standardized, comprehensive SA evaluation reports.

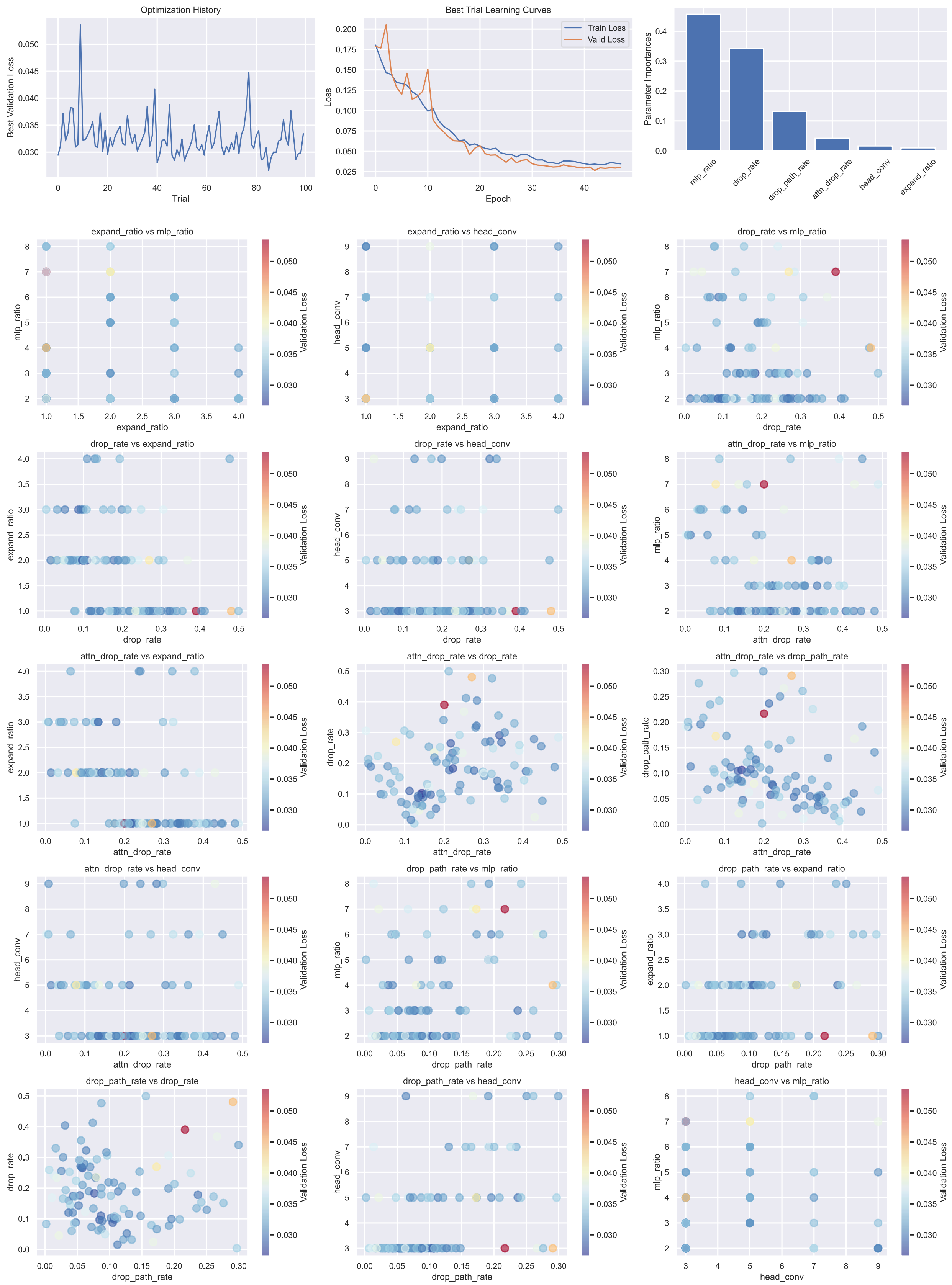

Supplementary Fig. 33 | Visualization of hyperparameter optimization process. (top row) Left: evolution of validation loss across 100 Bayesian optimization trials; Middle: learning curves of the best trial showing training and validation losses; Right: relative importance of optimized hyperparameters. (bottom rows) Pairwise relationship plots between different hyperparameters, where point colors indicate validation loss values (darker red indicates higher loss, darker blue indicates lower loss). The *mlp_ratio* demonstrated the strongest influence on model performance.

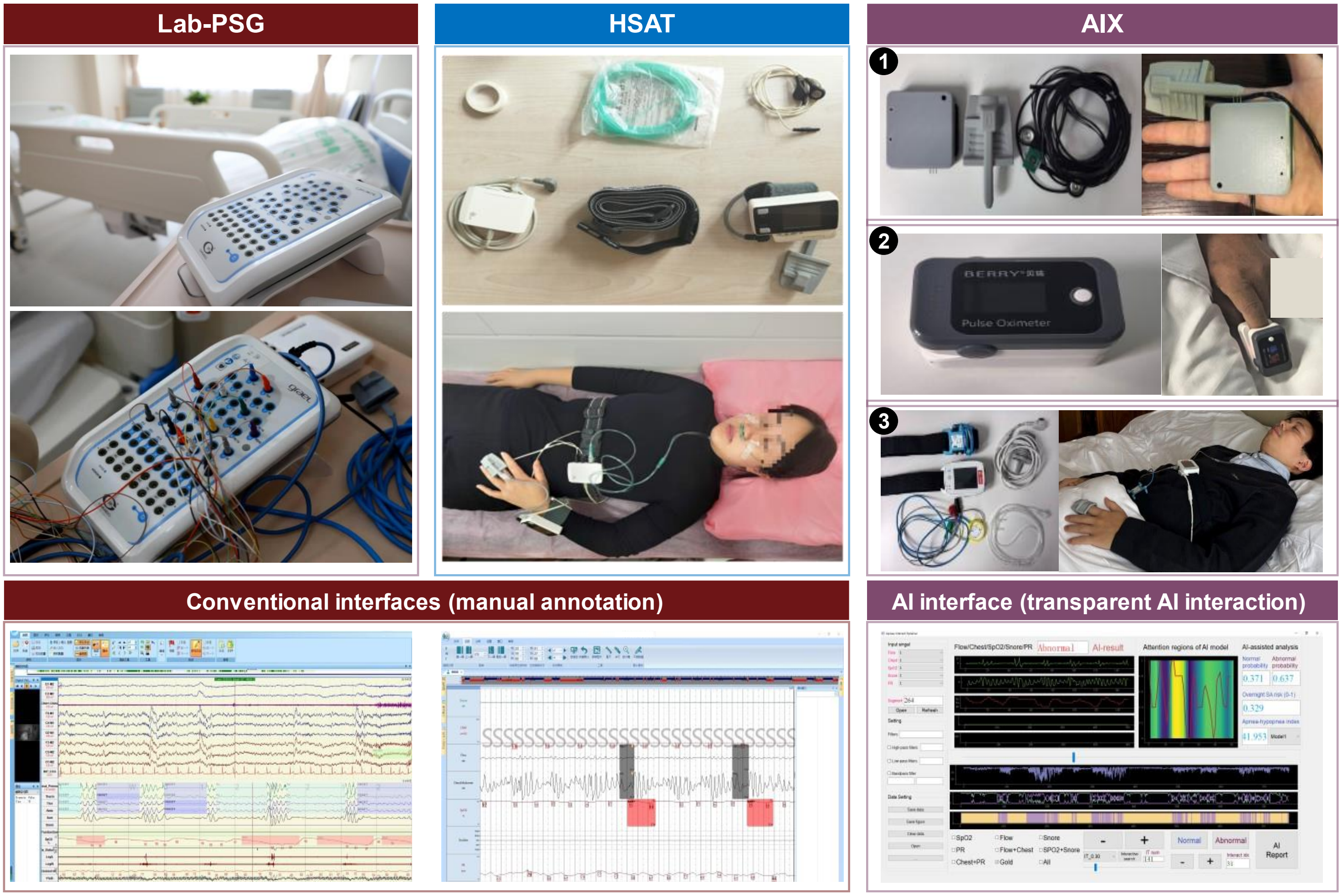

Parameters	Lab-PSG	HSAT	AIX
**Equipment**	~$68,000	$~14,000	(1)~$70 (2)~$15 (3)~$25,000
**Per-test**	$1,000-1,500	$200-400	<$10
**Technical time**	~3-4 hours	~1 hour	~1-10 minutes

Supplementary Fig. 34 | Cost and operational comparison between Lab-PSG, HSAT, and the proposed AIX system for SA diagnosis. AIX supports flexible monitoring channels while maintaining significantly lower equipment costs, per-test expenses, and technical time requirements. With transparent AI-assisted interaction logic, AIX is compatible with wearable devices, single-channel finger pulse oximeters, and portable home SA monitoring PSG configurations.

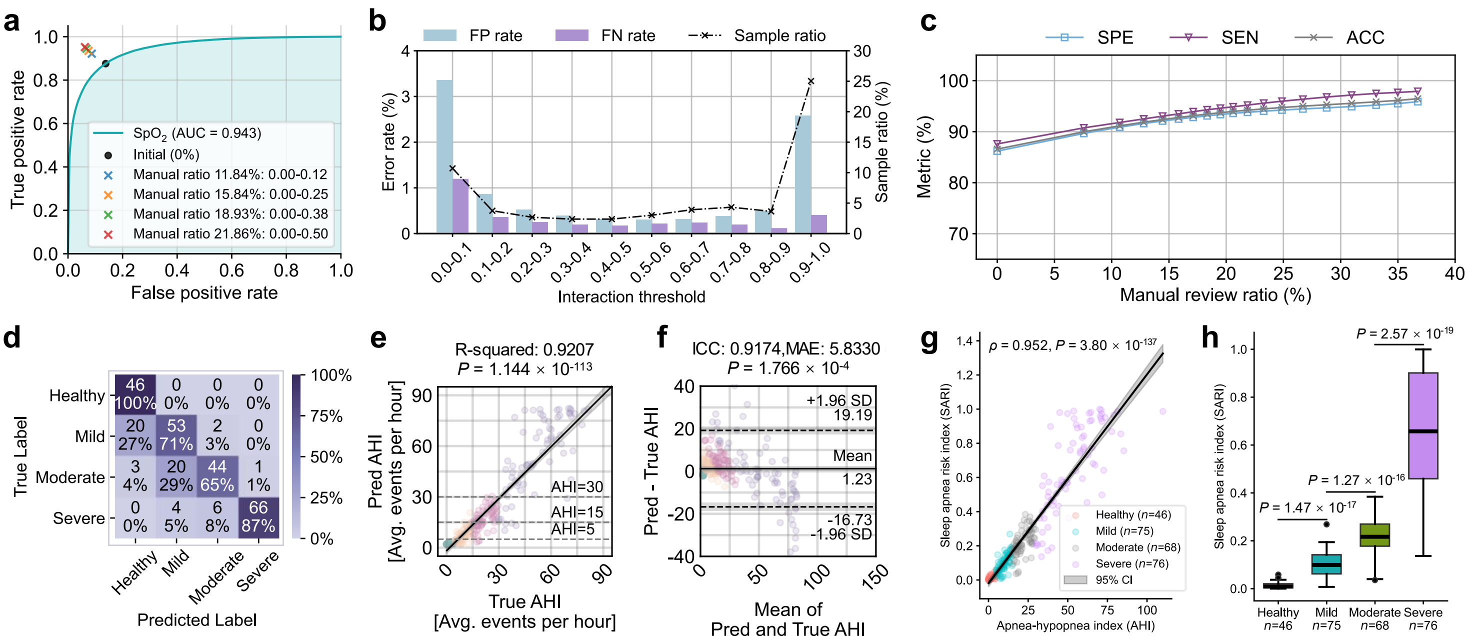

Supplementary Fig. 35 | AIX real-world validation on FDU-HSH prospective cohort (*n*=265 subjects). (a) ROC curve demonstrating single-channel SpO_2-based binary respiratory event classification performance. (b) Ternary relationship between FP rate, FN rate, and sample ratio across different interaction threshold intervals. (c) Binary classification performance metrics (SPE, SEN, ACC) under varying manual review ratios. (d) Confusion matrix for four-level SA severity classification, showing ACC=0.789 and $F1_{Macro}$=0.788 using SpO_2-only configuration. (e) Scatter plot of manual AHI versus predicted AHI with regression line (R^2=0.9207, two-sided $P=1.144\times10^{-113}$). (f) Bland-Altman plot comparing predicted and true AHI values (ICC=0.9174, MAE=5.8330, two-sided Wilcoxon signed-rank test $P=1.766\times10^{-4}$). (g) Spearman's ρ between SARI and AHI values (ρ=0.952, two-sided $P=3.80\times10^{-137}$). (h) Box plot of SARI distributions across severity groups with significant differences between adjacent categories (two-tailed Mann-Whitney U test, $P=1.47\times10^{-17}$, $P=1.27\times10^{-16}$, $P=2.57\times10^{-19}$). The model maintains consistent classification and regression performance on this independent prospective validation cohort (*n*=265 subjects) compared to the retrospective FDU-HSH cohort (*n*=327 subjects).

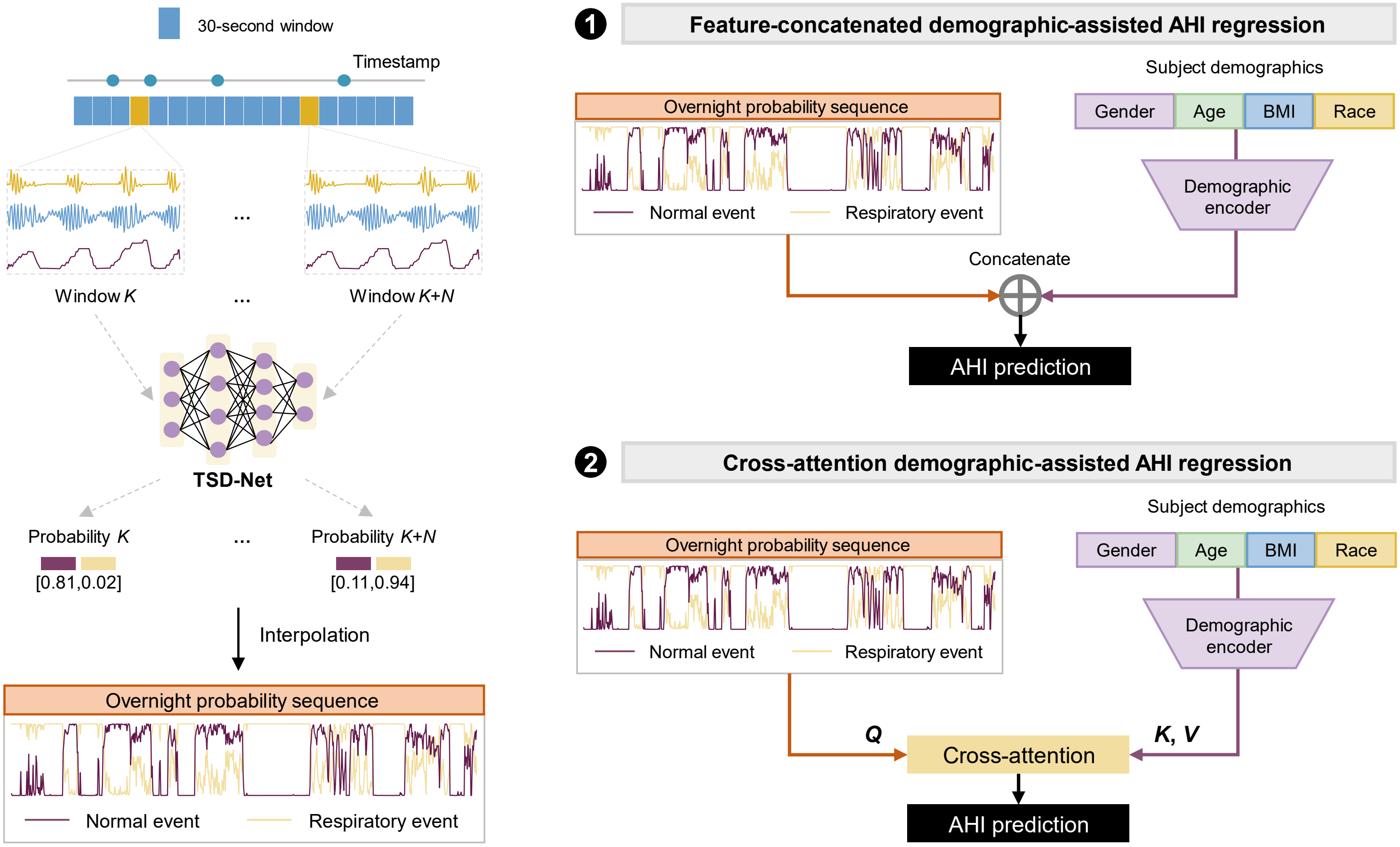

Supplementary Fig. 36 | Overview of the demographic-assisted AHI regression framework with two distinct integration approaches. Left: initial overnight probability sequence generation using a sliding window (30-second) approach. Multiple physiological signal windows are processed through a TSD-Net to generate event probabilities, which are then interpolated to form a continuous overnight probability sequence distinguishing between normal and respiratory events. Right: two proposed methods for integrating demographic information into AHI regression: (1) A feature-concatenation approach where demographic features are encoded and directly concatenated with the probability sequence features before AHI prediction. (2) A cross-attention mechanism where the probability sequence features serve as queries (*Q*) while demographic features act as keys (*K*) and values (*V*), enabling dynamic demographic-aware feature enhancement for AHI prediction. Patient demographics including gender, age, BMI, and race are processed through a dedicated demographic encoder in both approaches. The framework demonstrates two distinct strategies for leveraging subject-specific demographic information to enhance AHI regression performance.

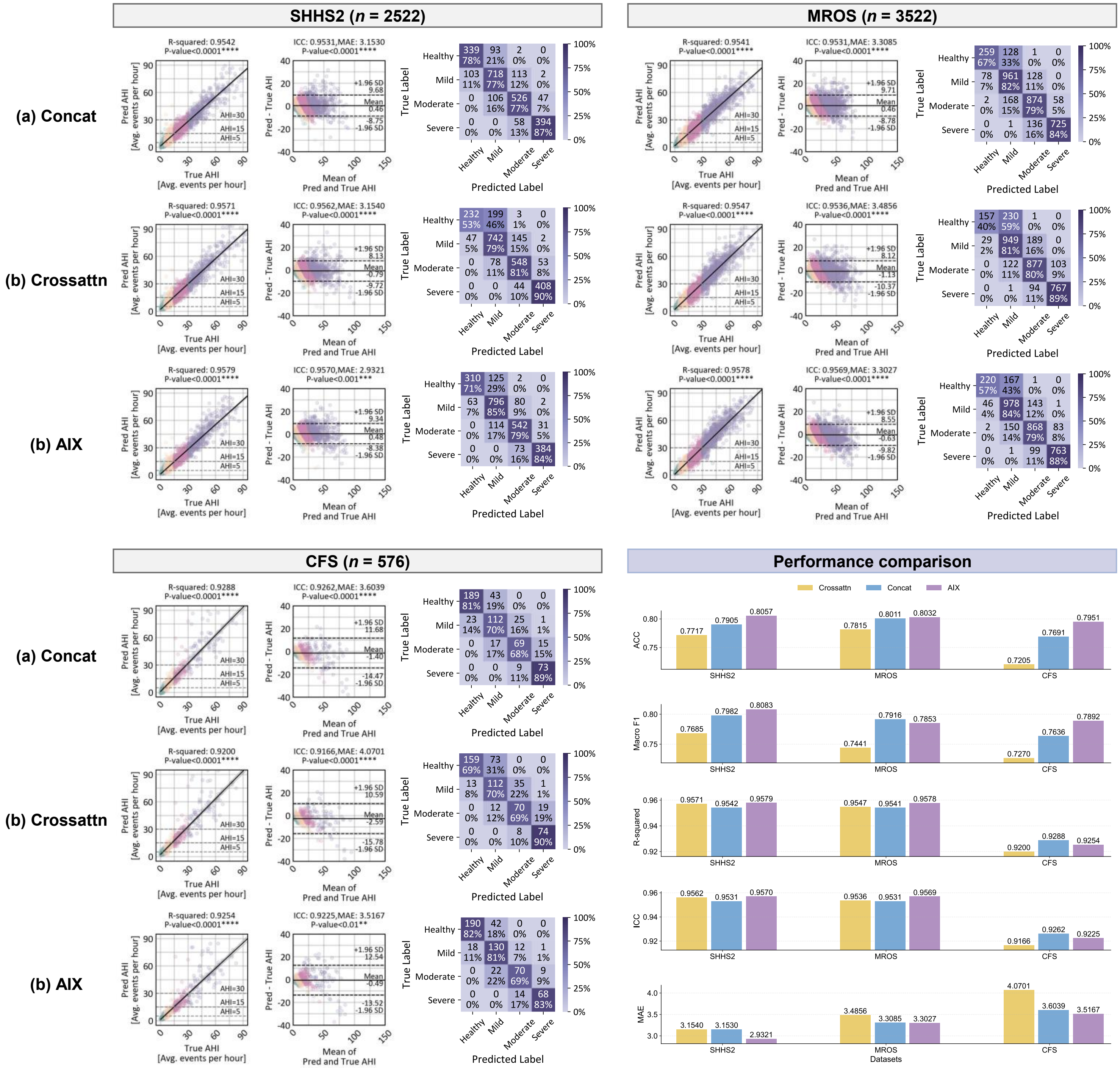

Supplementary Fig. 37 | Comparison of three AHI regression approaches using multi-channel Gold signals: concatenation (demographic-signal feature concatenation), cross-attention (demographic-signal attention-based feature fusion), and AIX (without demographic features) across SHHS2, MROS, and CFS cohorts. Performance evaluation includes regression analysis (left column, with R^2 values and two-sided p-values), Bland-Altman plots (middle column, showing mean differences with 95% limits of agreement), and confusion matrices (right column, displaying four-level severity classification). The bottom panel presents comparative metrics including ACC, $F1_{Macro}$, R^2, ICC, and MAE values across all methods and cohorts.

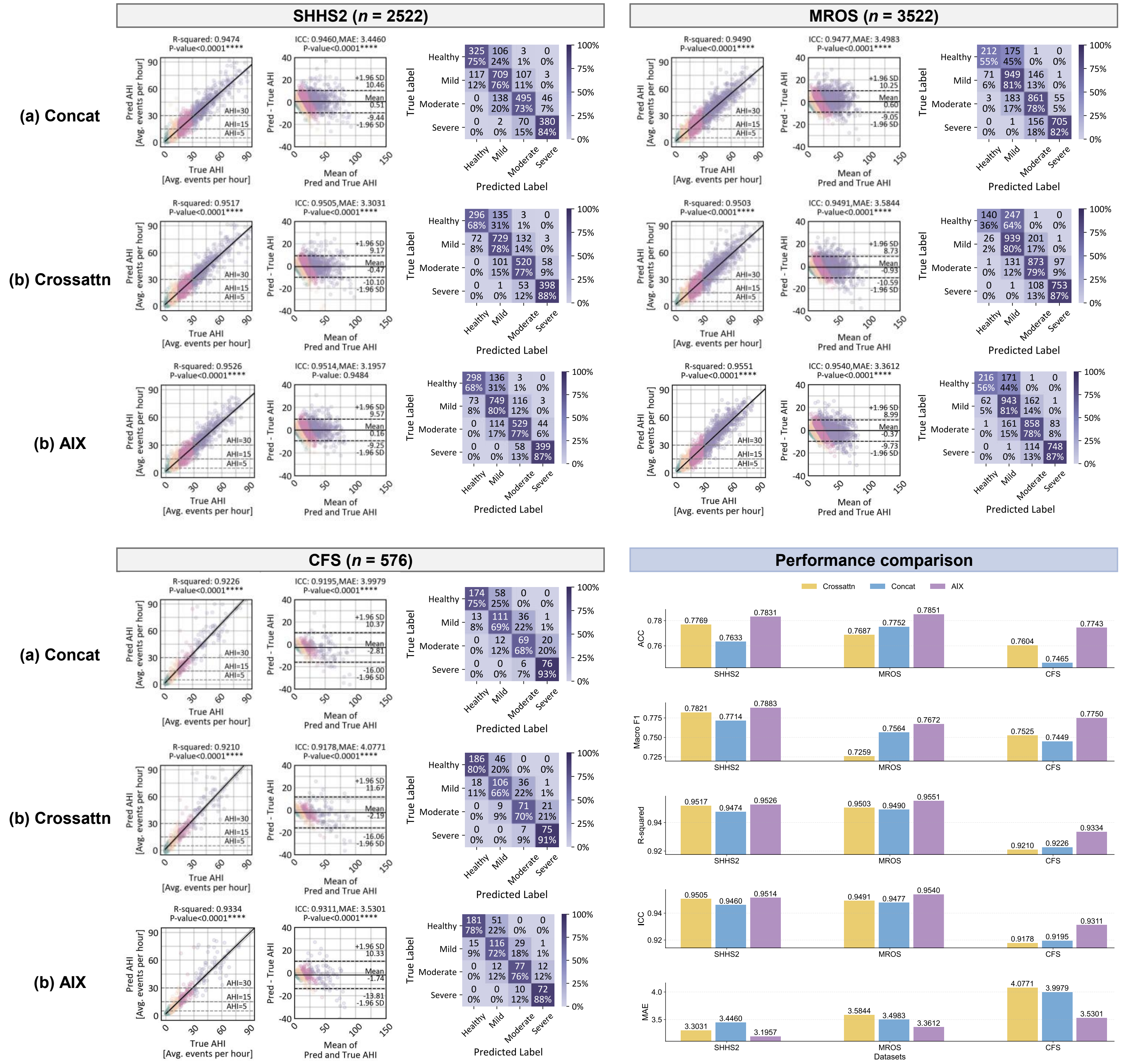

Supplementary Fig. 38 | Comparison of three AHI regression approaches using single-channel SpO_2 signals: concatenation (demographic-signal feature concatenation), cross-attention (demographic-signal attention-based feature fusion), and AIX (without demographic features) across SHHS2, MROS, and CFS cohorts. Performance evaluation includes regression analysis (left column, with R^2 values and two-sided p-values), Bland-Altman plots (middle column, showing mean differences with 95% limits of agreement), and confusion matrices (right column, displaying four-level severity classification). The bottom panel presents comparative metrics including ACC, $F1_{Macro}$, R^2, ICC, and MAE values across all methods and cohorts.

Architecture for cross-modal alignment of physiological multi-channel signals and demographics

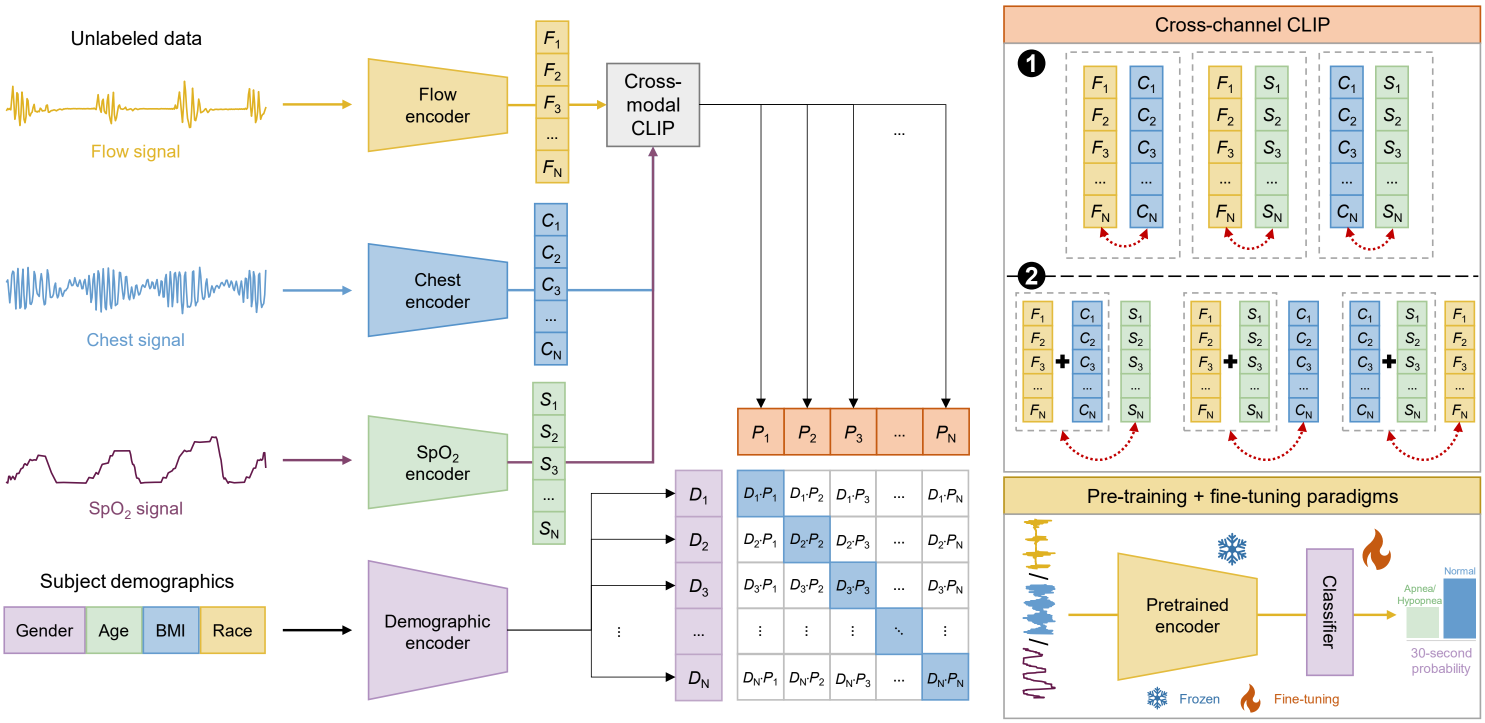

Supplementary Fig. 39 | A CLIP-inspired architecture for cross-modal alignment of physiological multi-channel signals and demographics. The architecture consists of four parallel encoding paths: three signal encoders (Flow, Chest, and SpO_2) for processing physiological signals, and a demographic encoder for subject characteristics (gender, age, BMI, race). Each signal passes through its respective encoder to generate feature embeddings (F_N, C_N, S_N), while demographics are encoded into D_N features. In the cross-modal CLIP module, P_N represents each individual physiological signal channel (F_N, C_N, or S_N) that is separately aligned with demographic features. The framework implements two types of CLIP-based learning strategies: cross-modal CLIP between signals and demographics, and cross-channel CLIP among physiological signals which includes pairwise alignment and leave-one-out alignment patterns (as shown in panel 1 and 2). The architecture enables transfer learning by freezing the pretrained encoders and fine-tuning only the classification head for downstream tasks.

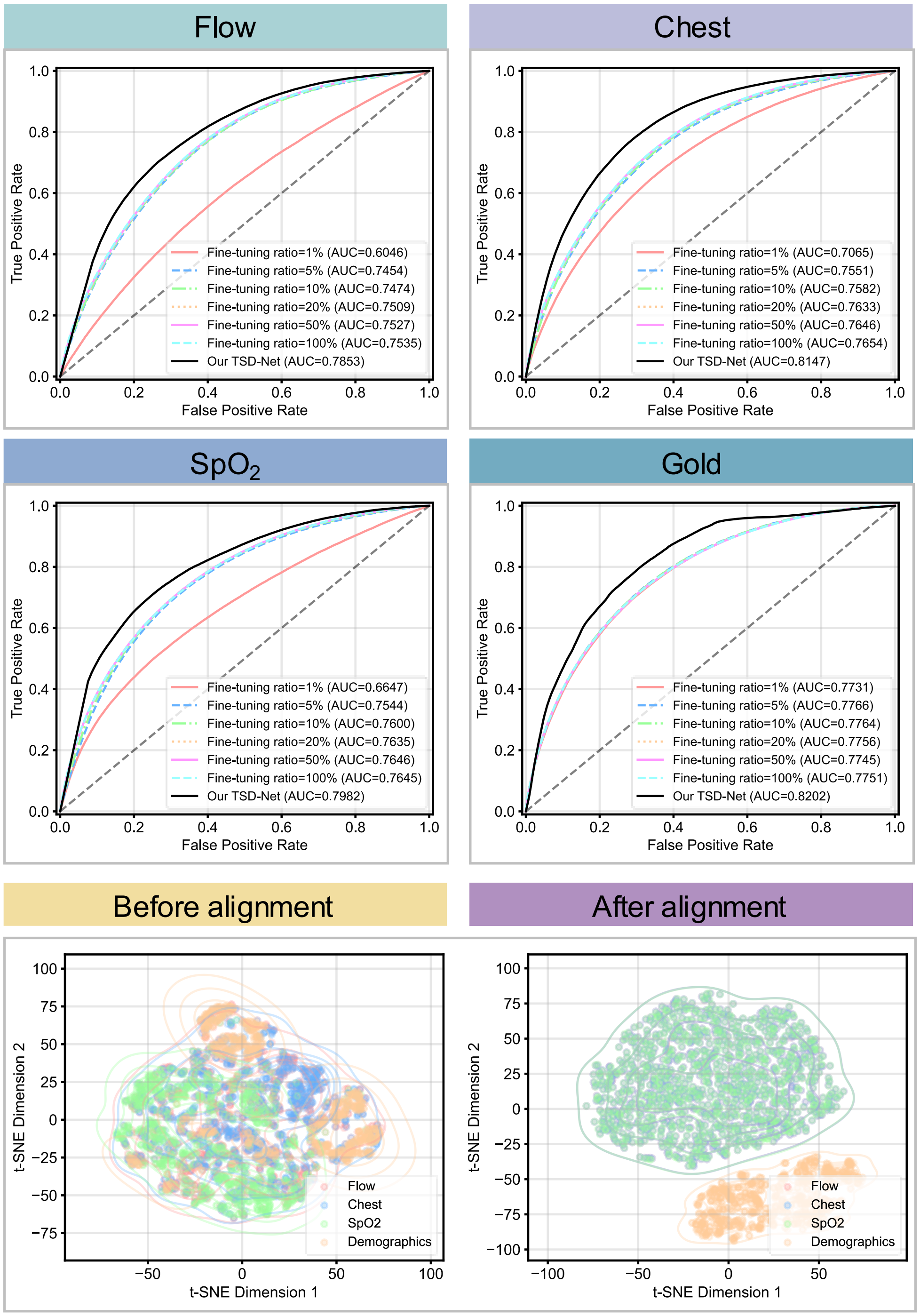

Supplementary Fig. 40 | Performance comparison of the proposed cross-modal alignment framework. (top) ROC curves comparing our CLIP-inspired framework with different fine-tuning ratios (1%, 5%, 10%, 20%, 50%, and 100%) of the complete SHHS1 contrastive learning training data across four channel configurations (Flow, Chest, SpO_2, and Gold standard combination), against the supervised TSD-Net. The evaluation is performed on 400,000 test samples (100,000 from each of SHHS2, MESA, MROS, and CFS cohorts). While our self-supervised approach shows promising results, the supervised TSD-Net achieved better performance across all configurations. (bottom) t-SNE visualization of feature embeddings before and after applying the proposed CLIP-based architecture.
