# Supplementary material for: Transparent artificial intelligence-enabled interpretable and interactive sleep apnea assessment across flexible monitoring scenarios
Source: Nat Commun. 2025 Aug 14;16:7548. doi: 10.1038/s41467-025-62864-x (PMC12354915; doi:10.1038/s41467-025-62864-x)
Supplement: Supplementary file 2 — Description of Additional Supplementary File [file 41467_2025_62864_MOESM2_ESM.pdf]

The Description of Additional Supplementary Files

Supplementary Movie 1. Background introduction and AIX operating interface demonstration. Introduction to global disease distribution, current technological bottlenecks, and complete operational workflow of the AIX system.

Supplementary Movie 2. AIX applications in professional scenarios. Analysis process of multi-channel signals from polysomnography equipment in sleep monitoring laboratories using the AIX system.

Supplementary Movie 3. AIX applications in home-based scenarios. Application potential of AIX using single-channel blood oxygen signals from portable home-based devices.
