# Supplementary material for: Transparent artificial intelligence-enabled interpretable and interactive sleep apnea assessment across flexible monitoring scenarios
Source: Nat Commun. 2025 Aug 14;16:7548. doi: 10.1038/s41467-025-62864-x (PMC12354915; doi:10.1038/s41467-025-62864-x)
Supplement: Supplementary file 6 — Reporting Summary [file 41467_2025_62864_MOESM6_ESM.pdf]

Corresponding author(s): Cuiwei Yang, Huan Yu

Last updated by author(s): Jul 12, 2025

Reporting Summary

Nature Portfolio wishes to improve the reproducibility of the work that we publish. This form provides structure for consistency and transparency in reporting. For further information on Nature Portfolio policies, see our Editorial Policies and the Editorial Policy Checklist.

Statistics

For all statistical analyses, confirm that the following items are present in the figure legend, table legend, main text, or Methods section.

n/a	Confirmed	
☐	☒	The exact sample size (n) for each experimental group/condition, given as a discrete number and unit of measurement
☐	☒	A statement on whether measurements were taken from distinct samples or whether the same sample was measured repeatedly
☐	☒	The statistical test(s) used AND whether they are one- or two-sided<br>*Only common tests should be described solely by name; describe more complex techniques in the Methods section.*
☐	☒	A description of all covariates tested
☐	☒	A description of any assumptions or corrections, such as tests of normality and adjustment for multiple comparisons
☐	☒	A full description of the statistical parameters including central tendency (e.g. means) or other basic estimates (e.g. regression coefficient) AND variation (e.g. standard deviation) or associated estimates of uncertainty (e.g. confidence intervals)
☐	☒	For null hypothesis testing, the test statistic (e.g. F, t, r) with confidence intervals, effect sizes, degrees of freedom and P value noted<br>*Give P values as exact values whenever suitable.*
☒	☐	For Bayesian analysis, information on the choice of priors and Markov chain Monte Carlo settings
☒	☐	For hierarchical and complex designs, identification of the appropriate level for tests and full reporting of outcomes
☐	☒	Estimates of effect sizes (e.g. Cohen's d, Pearson's r), indicating how they were calculated

Our web collection on statistics for biologists contains articles on many of the points above.

Software and code

Policy information about availability of computer code

Data collection	Data was collected from seven independent cohorts: five public cohorts from National Sleep Research Resource (NSRR) including SHHS1, SHHS2, MESA, MROS, and CFS, along with two private cohorts from FDU-HSH (Retrospective cohort and Prospective cohort). All polysomnography (PSG) data contains three components: multichannel digital signals in EDF format, overnight annotation files in XML format, and clinical baseline information.
Data analysis	The analysis was performed using Python 3.9 with PyTorch (Ver. 1.13) for deep learning implementation. The model training was conducted on NVIDIA A100 GPU with 80GB VRAM. Key Python packages used include: 1. PyTorch for neural network models. 2. scikit-learn for machine learning algorithms. 3. SciPy for signal processing. 4. LightGBM for gradient boosting.<br>Code for the AIX system is available at https://github.com/fdu-harry/Apnea-Interact-Xplainer.

For manuscripts utilizing custom algorithms or software that are central to the research but not yet described in published literature, software must be made available to editors and reviewers. We strongly encourage code deposition in a community repository (e.g. GitHub). See the Nature Portfolio guidelines for submitting code & software for further information.

Data

Policy information about availability of data

All manuscripts must include a data availability statement. This statement should provide the following information, where applicable:

- Accession codes, unique identifiers, or web links for publicly available datasets
- A description of any restrictions on data availability
- For clinical datasets or third party data, please ensure that the statement adheres to our policy

The SHHS, MESA, MROS, and CFS cohorts are publicly available from the National Sleep Research Resource (NSRR) with direct access links: SHHS (https://sleepdata.org/datasets/shhs), MESA (https://sleepdata.org/datasets/mesa), MROS (https://sleepdata.org/datasets/mros), and CFS (https://sleepdata.org/datasets/cfs). These datasets can be accessed after appropriate deidentification and obtaining necessary permissions via their online portal at https://www.sleepdata.org/. The FDU-HSH sleep study data cannot be made publicly available due to ethical approval requirements and participant privacy protections under institutional policies. Access restrictions exist because the data contain sensitive participant information subject to Institutional Review Board (IRB) approval. Researchers may request access to the FDU-HSH data by contacting the corresponding authors, with requests evaluated based on legitimate research purposes. Access requires completion of formal data use agreements with Huashan Hospital, Fudan University, and is limited to noncommercial academic research purposes. The source codes and trained models used in this study have been made available at https://github.com/fdu-harry/Apnea-Interact-Xplainer under an open source license for academic research purposes.

Research involving human participants, their data, or biological material

Policy information about studies with human participants or human data. See also policy information about sex, gender (identity/presentation), and sexual orientation and race, ethnicity and racism.

Reporting on sex and gender

We analyzed sex-based differences across all available measurement data from SHHS1, SHHS2, MROS, CFS, and FDU-HSH (male, n=7,013; female, n=4,694; MESA excluded due to missing BMI data). Sex information was collected through self-reporting during clinical visits. Gender-specific analyses revealed different correlations between AHI-BMI, AHI-age, and age-BMI across male and female groups, with females showing stronger age-AHI correlation ($\rho=0.3085$, $P<0.0001$) compared to males ($\rho=0.0206$, $P=0.0846$).

Reporting on race, ethnicity, or other socially relevant groupings

The study included participants from multiple ethnic backgrounds across seven cohorts:
1. SHHS1: Black (8.45%), White (84.70%), Other (6.85%).
2. SHHS2: Black (6.78%), White (86.99%), Other (6.23%).
3. MESA: Asian (12.08%), Black (26.60%), White (37.70%), Hispanic (23.62%),.
4. MROS: Asian (2.95%), Black (3.29%), White (90.60%), Hispanic (1.96%), Other (1.21%).
5. CFS: Black (55.38%), White (42.53%), Multiple (2.08%).
6. FDU-HSH (Retrospective cohort): Asian (100.00%).
7. FDU-HSH (Prospective cohort): Asian (100.00%).

Ethnic information was collected through self-reporting during enrollment. Model performance was evaluated across different ethnic groups to ensure fairness and generalizability.

Population characteristics

The study population characteristics varied across cohorts:
1. Age ranges: 39-90 years (SHHS), 54-90 years (MESA), 67-90 years (MROS), 18-88.5 years (CFS), 18-85 years (FDU-HSH).
2. BMI distribution reported for each severity group.
3. Clinical variables analyzed: age, BMI, blood pressure, sleep duration, arousal index.
4. Comprehensive demographic data presented in Supplementary Tables 1,5.

Disease severity distribution (healthy: AHI<5, mild: 5≤AHI<15, moderate: 15≤AHI<30, severe: AHI≥30) was analyzed across all populations.

Recruitment

Participants were recruited through multiple established sleep research programs:
1. SHHS1 and SHHS2: Participants from existing cardiovascular cohort studies.
2. MESA: Multi-ethnic population from six collaborating centers.
3. MROS: Male participants from osteoporotic fractures study.
4. CFS: Family-based recruitment focusing on sleep apnea.
5. FDU-HSH (Retrospective cohort): Clinical recruitment from sleep center subjects (January 2021 to December 2023).
6. FDU-HSH (Prospective cohort): Clinical recruitment from sleep center subjects (December 2023 to December 2024).

Ethics oversight

The FDU-HSH sleep research protocol was approved by the IRB of Huashan Hospital, Fudan University (Approval No. KY2021-811). The NSRR databases (SHHS, MESA, MROS, CFS) were conducted under their respective IRB approvals. All participants provided written informed consent.

Note that full information on the approval of the study protocol must also be provided in the manuscript.

Field-specific reporting

Please select the one below that is the best fit for your research. If you are not sure, read the appropriate sections before making your selection.

☒ Life sciences ☐ Behavioural & social sciences ☐ Ecological, evolutionary & environmental sciences

For a reference copy of the document with all sections, see nature.com/documents/nr-reporting-summary-flat.pdf

Life sciences study design

All studies must disclose on these points even when the disclosure is negative.

Sample size	Total sample size consists of 15,807 PSG records from seven independent cohorts with over 130,000 hours of overnight digital signals. Sample sizes were determined by data availability and quality criteria:<br>1. SHHS1: n=5,255 for model training (All-Sub SHHS model); n=2,789 for model training (Non-overlap SHHS model).<br>2. External validation cohorts: SHHS1 (n=2,466), SHHS2 (n=2,522), MESA (n=1,846), MROS (n=3,522), CFS (n=576).<br>3. Real-world validation: FDU-HSH (Retrospective cohort, n=327), FDU-HSH (Prospective cohort, n=265).<br>These sample sizes are sufficient for robust model development and validation, exceeding those of previous studies in the field (typically <100 subjects).
Data exclusions	Records were excluded based on pre-established criteria: 1. Missing required modality signals (Flow, Chest, or SpO2). 2. Sleep duration under 4 hours. 3. Missing AHI annotations. 4. Signal quality criteria: Oxygen saturation artifact segments excluded; Flow/chest signals with SD below 0.0001 (flat-line threshold) excluded; All exclusion criteria were determined prior to analysis.
Replication	Model performance was verified through multiple validation approaches:<br>1. External validation on five independent cohorts (SHHS1, SHHS2, MESA, MROS, CFS, n=10,932).<br>2. Real-world validation (Retrospective cohort n=327, Prospective cohort n=265).<br>3. Cross-population stability assessment across different demographic and ethnic groups.<br>4. Performance evaluation under different modality configurations (Gold channel vs. SpO2 channel).<br>Results showed consistent performance across all validation cohorts with R^2 values of 0.92-0.96.
Randomization	Data augmentation employed random noise perturbations: 1. Gaussian noise with randomly initialized SD (0.005-0.01). 2. Random 8:2 split of training and validation data in each epoch during TSD-Net training to expose model to more data combinations. 3. For LightGBM model training, 90% of features were randomly selected in each iteration to prevent overfitting.
Blinding	Model evaluation was conducted using blinded external test cohorts:<br>1. Model training used only SHHS1 data (All-Sub SHHS model and Non-overlap SHHS model).<br>2. Performance evaluation on independent cohorts (SHHS1, SHHS2, MESA, MROS, CFS, FDU-HSH).<br>3. Automated evaluation metrics (ACC, SEN, SPE, etc.) were calculated without human intervention.<br>This design ensures unbiased assessment of model generalization capability.

Reporting for specific materials, systems and methods

We require information from authors about some types of materials, experimental systems and methods used in many studies. Here, indicate whether each material, system or method listed is relevant to your study. If you are not sure if a list item applies to your research, read the appropriate section before selecting a response.

Materials & experimental systems

n/a	Involved in the study	
☒	☐	Antibodies
☒	☐	Eukaryotic cell lines
☒	☐	Palaeontology and archaeology
☒	☐	Animals and other organisms
☐	☒	Clinical data
☒	☐	Dual use research of concern
☒	☐	Plants

Methods

n/a	Involved in the study	
☒	☐	ChIP-seq
☒	☐	Flow cytometry
☒	☐	MRI-based neuroimaging

Clinical data

Policy information about clinical studies

All manuscripts should comply with the ICMJE guidelines for publication of clinical research and a completed CONSORT checklist must be included with all submissions.

Clinical trial registration	Not applicable. This study involves computational analysis and validation of an AI system using sleep physiological signals and does not constitute a clinical trial.
Study protocol	The FDU-HSH sleep study protocol was approved by the IRB of Huashan Hospital, Fudan University (Approval No. KY2021-811). For NSRR databases (SHHS, MESA, MROS, CFS), protocols are publicly available through the National Sleep Research Resource (sleepdata.org).
Data collection	Data collection spans multiple timeframes and settings:SHHS: Visit 1 (1995-1998), Visit 2 (2001-2003). MESA: Multi-center sleep study from six collaborating centers. MROS: Sleep study conducted between December 2003 and March 2005. CFS: Family-based sleep study collection. FDU-HSH: Data collected from January 2021 to December 2023 (Retrospective) and December 2023 to December 2024 (Prospective).

All PSG data includes: Multi-channel digital signals in EDF format. Overnight annotation files in XML format. Clinical baseline information. Signals were resampled to uniform sampling rates and standardized according to AASM guidelines.

Outcomes

Primary outcomes: 1. AHI prediction accuracy (R^2=0.92-0.96 across test cohorts). 2. SA severity classification (ACC=0.74-0.81 for four-level classification). 3. The overall sensitivity for early SA detection across all cohorts was 0.970.
Secondary outcomes: 1. Model performance across different ethnic groups. 2. Performance under simplified monitoring configurations. 3. SARI assessment. 4. Transparent decision interpretation through scale diffusion mechanism.
Outcomes were assessed using standardized metrics: ACC, SEN, SPE, PPV, F1; R^2 and ICC for regression analysis; ROC curves with 95% CIs; MAE with SD.

Plants

Seed stocks

N/A

Novel plant genotypes

N/A

Authentication

N/A
